# Body mass index and risk of over 100 cancer forms and subtypes in 4.1 million individuals in Sweden: the Obesity and Disease Development Sweden (ODDS) pooled cohort study

**DOI:** 10.1016/j.lanepe.2024.101034

**Published:** 2024-08-20

**Authors:** Ming Sun, Marisa da Silva, Tone Bjørge, Josef Fritz, Innocent B. Mboya, Mats Jerkeman, Pär Stattin, Jens Wahlström, Karl Michaëlsson, Bethany van Guelpen, Patrik K.E. Magnusson, Sven Sandin, Weiyao Yin, Ylva Trolle Lagerros, Weimin Ye, Bright Nwaru, Hannu Kankaanranta, Lena Lönnberg, Abbas Chabok, Karolin Isaksson, Nancy L. Pedersen, Sölve Elmståhl, Lars Lind, Linnea Hedman, Christel Häggström, Tanja Stocks

**Affiliations:** a Department of Translational Medicine, Lund University, Malmö, Sweden; b Department of Global Public Health and Primary Care, University of Bergen, Bergen, Norway; c Cancer Registry of Norway, Norwegian Institute of Public Health, Oslo, Norway; d Institute of Medical Statistics and Informatics, Medical University of Innsbruck, Innsbruck, Austria; e Division of Oncology, Lund University, Lund, Sweden; f Department of Surgical Sciences, Uppsala University, Uppsala, Sweden; g Section of Sustainable Health, Department of Public Health and Clinical Medicine, Umeå University, Umeå, Sweden; h Medical Epidemiology, Department of Surgical Sciences, Uppsala University, Uppsala, Sweden; i Department of Diagnostics and Intervention, Oncology, Umeå University, Umeå, Sweden; j Wallenberg Centre for Molecular Medicine, Umeå University, Umeå, Sweden; k Department of Medical Epidemiology and Biostatistics, Karolinska Institutet, Stockholm, Sweden; l Department of Psychiatry, Icahn School of Medicine at Mount Sinai, New York, USA; m Division of Clinical Epidemiology, Department of Medicine, Solna, Karolinska Institutet, Stockholm, Sweden; n Center for Obesity, Academic Specialist Center, Stockholm Health Services, Stockholm, Sweden; o Krefting Research Centre, Department of Internal Medicine and Clinical Nutrition, Institute of Medicine, Sahlgrenska Academy, University of Gothenburg, Gothenburg, Sweden; p Wallenberg Centre for Molecular and Translational Medicine, University of Gothenburg, Gothenburg, Sweden; q Department of Respiratory Medicine, Seinäjoki Central Hospital, Seinäjoki, Finland; r Faculty of Medicine and Health Technology, Tampere University, Tampere, Finland; s Center for Clinical Research, Region Västmanland, Uppsala University, Västerås, Sweden; t Department of Clinical Sciences Lund, Lund University, Lund, Sweden; u Department of Surgery, Kristianstad Hospital, Kristianstad, Sweden; v Department of Clinical Sciences Malmö, Lund University, Malmö, Sweden; w Department of Medical Sciences, Uppsala University, Uppsala, Sweden; x Northern Registry Centre, Department of Diagnostics and Intervention, Umeå University, Umeå, Sweden

**Keywords:** Obesity, Body mass index, Cancer

## Abstract

**Background:**

Obesity, assessed by body mass index (BMI), is an established risk factor for 13 cancers. We aimed to identify further potential obesity-related cancers and to quantify their association with BMI relative to that of established obesity-related cancers.

**Methods:**

Using Cox regression models on 4,142,349 individuals in Sweden (mean age 27.1 years at weight measurement), we calculated hazard ratios (HRs) for the association between BMI and the risk of 122 cancers and cancer subtypes, grouped by topography and morphology. Cancers with a positive association (i.e., HR >1) at an α-level of 0.05 for obesity (BMI ≥30 kg/m^2^) vs. normal weight (BMI 18.5–24.9 kg/m^2^) or per 5 kg/m^2^ higher BMI, for which obesity is not an established risk factor, were considered potentially obesity related.

**Findings:**

After 100.2 million person-years of follow-up, 332,501 incident cancer cases were recorded. We identified 15 cancers in men and 16 in women as potentially obesity related. These were cancers of the head and neck, gastrointestinal tract, malignant melanoma, genital organs, endocrine organs, connective tissue, and haematological malignancies. Among these, there was evidence of differential associations with BMI between subtypes of gastric cancer, small intestine cancer, cervical cancer, and lymphoid neoplasms (P values for heterogeneity in HRs <0.05). The HR (95% confidence interval) per 5 kg/m^2^ higher BMI was 1.17 (1.15–1.20) in men and 1.13 (1.11–1.15) in women for potential obesity-related cancers (51,690 cases), and 1.24 (1.22–1.26) in men and 1.12 (1.11–1.13) in women for established obesity-related cancers (84,384 cases).

**Interpretation:**

This study suggests a large number of potential obesity-related cancers could be added to already established ones. Importantly, the magnitudes of the associations were largely comparable to those of the already established obesity-related cancers. We also provide evidence of specific cancer subtypes driving some associations with BMI. Studies accounting for cancer-specific confounders are needed to confirm these findings.

**Funding:**

10.13039/501100004359Swedish Research Council, 10.13039/501100002794Swedish Cancer Society, Mrs. Berta Kamprad’s Cancer Foundation, Crafoord Foundation, 10.13039/100002002Cancer Research Foundation at the Department of Oncology, Malmö University Hospital, and China Scholarship Council.

**Research in context**

**Evidence before this study**

We searched for prospective epidemiological studies and meta-analyses on the association between body mass index (BMI) and cancer risk published in PubMed and Embase until December 22, 2023, using the search terms “obesity” OR “adiposity” OR “obese” OR “overweight” OR “body mass index” OR “BMI” OR “body fatness”, AND “cancer”, AND “risk” OR “incidence”. To date, the two largest studies on BMI and the risk of multiple cancers are a study from 2014 of 5 million UK adults and a study from 2021 of 3.5 million Spanish adults, both recording around 200,000 incident cancers during follow-up. The latest comprehensive reports summarising the evidence of the association of body size measures, primarily BMI, and cancer risk were performed by the International Agency for Research on Cancer (IARC) in 2016, the World Cancer Research Fund in 2018, and an umbrella review by Kyrgiou et al. in 2017. IARC concluded that 13 cancers were obesity related, most of these were confirmed by the other two sources. The evidence for rarer cancers and cancer subtypes is insufficient, and many of these have never been investigated in relation to BMI.

**Added value of this study**

This study of 4.1 million individuals in Sweden with over 330,000 incident cancers during follow-up adds evidence to the association between BMI and cancers not yet established obesity related. We found 15 cancers in men and 16 in women to be potentially obesity related. Some of these cancers have been indicated as potentially obesity-related with insufficient evidence in the reports or were associated with BMI in more recent meta-analyses or large studies, and for other cancers, evidence from the present study is novel. Replication of the findings is needed particularly for rare cancers.

**Implications of all the available evidence**

Previous epidemiological studies have provided strong evidence for several cancers to be obesity-related, and several more to be potentially linked to obesity but with inadequate evidence. Our study supports the positive associations between BMI and some, but not all, of these cancers, and suggests additional cancers, most of which are rare, to be potentially obesity related. This study also suggests some of the associations to be driven by specific cancer subtypes. The findings highlight the importance of obesity in risk assessments of cancer, emphasizing the necessity for public health strategies focusing on weight control for cancer prevention.

## Introduction

The high prevalence and rising incidence of overweight and obesity globally emphasises the necessity to detail the effect of obesity on morbidity.^1^ A growing number of studies suggest obesity to be a preventable cause of certain cancers. A report from the International Agency for Research on Cancer (IARC) in 2016 established the association between body mass index (BMI, kg/m^2^) and 13 cancers with sufficient evidence, including cancers of the oesophagus (adenocarcinoma), gastric cardia, colorectum, liver, gallbladder, pancreas, breast (postmenopausal), endometrium, ovary, meningioma, thyroid, multiple myeloma, and renal cell carcinoma.^2^ An umbrella review in 2017 based on 204 meta-analyses confirmed most of these as obesity-related cancers.^3^ More recently, a Mendelian randomisation study further strengthened the causality of obesity with cancers of the oesophagus (adenocarcinoma), colorectum, endometrium, ovary, kidney, and pancreas.^4^

Many large observational studies have investigated the association between BMI and cancer risk; however, these studies have mostly focused on more common site-specific cancers and rarely on morphological subtypes.^5^, ^6^, ^7^ Little evidence exists about the association between BMI and rarer cancers and cancer subtypes. The IARC report pointed out several cancers, such as diffuse large B-cell lymphoma and extrahepatic bile duct cancer, as potentially obesity related, but concluded that evidence to date was inadequate or limited.^2^

This study aimed to identify further potential obesity-related cancers and cancer subtypes in a large population in Sweden with long follow-up, therefore including a large number of incident cancer cases and detailed cancer categorisation according to topography and morphology. We also aimed to quantify the association between BMI and all potential obesity-related cancers relative to that of all established ones. We explored heterogeneity between cancer subtypes of potential and established obesity-related cancer sites with regard to their association with BMI, to identify any cancer subtypes more strongly associated with BMI than others.

## Methods

### Study population

We used data from the Obesity and Disease Development Sweden (ODDS) study, which is a pooling of large Swedish cohorts and national registers with individual-level information on height and weight, objectively measured (94%) or self-reported once or more between 1963 and 2020.^8^ Men in the Swedish Military Conscription Register (1969–2014, with mandatory conscription until 2010) and women in the Medical Birth Register (1982–2019) make up the majority (85%) of the study population. Weight of women in the Medical Birth Register was generally measured between eight and ten weeks of gestation, i.e., before any substantial pregnancy-related weight gain.^9^ Due to changes in weight recording in 1992 in the Medical Birth Register, a greater increase in body weight was observed in 1992 than expected. By estimating annual weight gain (temporal trends) in the Medical Birth Register, we corrected weight for the period prior to 1992, similar to other studies based on the Medical Birth Register.^10^ Self-reported information on smoking habits was obtained from some of the included cohorts.

The study was approved by the Swedish Ethical Review Authority (no: 2020-03846).

### Register linkages

Individuals in the study population were matched with data from several national registries by record linkages using the unique personal identity number assigned to each resident of Sweden. The Swedish Cancer Register, based on mandatory reporting of cancer diagnoses of the population since 1958 and capturing more than 95% of cancer diagnoses in Sweden, was used to identify all cancer diagnoses in the study population from 1963 to 2019.^11^ We retrieved information on tumour characteristics of prostate cancers diagnosed since 1998 from the National Prostate Cancer Register. We also retrieved date of death from the Cause of Death Register since 1958,^12^ sex, date of birth and emigration, country of birth, and marital status from the Total Population Register since 1968,^13^ and education level from the Longitudinal integrated database for health insurance and labour market studies (LISA) since 1990^14^ and from the Population and housing censuses (for early years, 1960–70). These registers capture more than 99% of the Swedish population with overall high validity, for example, the information on highest attained education in the LISA has shown an accuracy of 85%.^14^

### Cancer categorisation

**Fig. 1:**
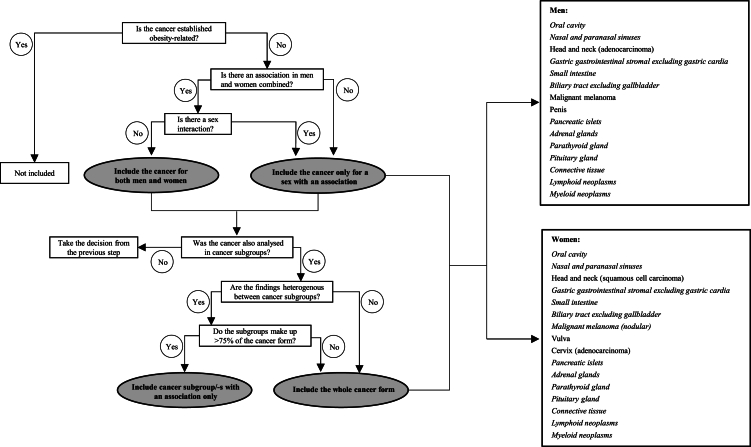
Flow-chart for decision making of inclusion of a cancer as potentially obesity related. A two-sided α-level of 0.05 for an increased cancer risk in obesity vs. normal weight or per 5 kg/m^2^ higher body mass index was used to indicate a cancer as obesity-related. In a final step amongst cancers in grey-shaded circles, any remaining cancer subtypes overlapping with an established obesity-related cancer, e.g. gastric-cardia and gallbladder cancer, were excluded. Potential obesity-related cancers are listed for men and women separately on the right side, with cancers included for both men and women in italics.

We classified cancers using International Classification of Diseases (ICD) codes, WHO/HS/CANC/24.1 (Swedish PAD codes), and ICD-O/2 and -O/3 codes (Swedish SNOMED codes). The codes used to classify each cancer are shown in the Appendix (pp 2–4). All primary malignant cancers, and a limited number of potentially harmful cancers of borderline or malignant potential or benign (e.g. of the ovaries, central nervous system, endocrine and haematological malignancies) and *in situ* urothelial cancers, were included, in accordance with the Swedish National Board of Health and Welfare (Appendix p 5). First-incident primary cancers with at least 100 cases diagnosed during follow-up were included as outcomes. We considered the 13 cancers concluded as obesity-related by the IARC, to be established obesity-related cancers.^2^ We used an explorative decision algorithm to define a cancer as potentially obesity related (Fig. 1). Associations with an increased risk for either obesity (BMI ≥30 kg/m^2^) vs. normal weight (BMI 18.5–24.9 kg/m^2^) or per 5 kg/m^2^ higher BMI at a two-sided α-level of 0.05 were considered a “potential obesity-related cancer”.

### Participant selection

The ODDS study population includes 4,295,859 individuals with 7,733,901 weight assessments (observations) at age 17 or older. We excluded observations with recalled weight, missing height, extreme values of weight, height, or BMI, mismatching dates, or with a cancer diagnosis before the weight assessment. The first observation with information on smoking status (if available) was selected as baseline examination, resulting in a study population of 4,142,349 individuals with one baseline observation each (Appendix p 29). The number of individuals in each cohort and the years of baseline examination are shown in the Appendix (p 6).

### Statistical analysis

The distribution of continuous variables was presented as mean with standard deviation (SD) if the variable had approximately a normal distribution and as median with interquartile range (IQR) otherwise. Categorical variables were summarised using absolute and relative frequencies. BMI was analysed in relation to cancer risk per 5 kg/m^2^ if the number of cases was at least 100, and in categories (underweight, normal weight, overweight, and obesity) if the number of cases was at least 250. Hazard ratios (HRs) with 95% confidence intervals (CIs) were estimated by Cox regression models with attained age as the underlying time metric and counting person-years at risk from baseline until the diagnosis of a cancer, censoring at date of another cancer, death, emigration, or until the end of follow-up on 31 December 2019, whichever came first. For cancers that could only be correctly classified by later ICD codes than ICD7, follow-up started at the year of the start of the respective ICD edition or at baseline, whichever came later (Appendix pp 2–4). We stratified the models by sex and calendar year of birth (<1950, 1950–1959, 1960–1969, 1970–1979, ≥1980), and adjusted all analyses for baseline age (continuous), weight assessment from the Medical Birth Register (yes/no), mode of weight and height assessment (objectively measured/self-reported), respectively, marital status, education level, and birth country. Missing data for marital status, education level and birth country were treated as separate categories (max 1% missing in each variable). For the 20 cancers most strongly associated with height in our data, we performed the analysis additionally adjusted for height (Appendix p 7). As the results showed no or minor changes after adjustment, height was not included in the final model. For smoking-related cancers, defined as cancers with an HR of 1.5 or higher for current vs. never smokers in our data (Appendix p 8), we repeated the Cox models in individuals with smoking information available (never/former/current smoker). In these individuals, we performed smoking-unadjusted and adjusted analyses, and analysed never-smokers separately. For cancers with at least 250 cases, we used Cox models with restricted cubic splines to visualise the shape of association with cancer risk across the BMI range. The splines were fitted using the same adjustments and strata as described above. Non-linearity was assessed, testing the null hypothesis of equal spline coefficients using the post-estimation Wald test. In all analyses, potential sex interactions were investigated using the Wald test by adding product terms of sex and BMI into the model. The heterogeneity of HR per 5 kg/m^2^ higher BMI between cancer subtypes was calculated using the Lunn and McNeil duplication method.^15^

Schoenfeld residual statistics were used to test the proportional hazards assumption of the Cox models. For cancers for which the proportional hazards assumption appeared to be violated for BMI, flexible parametric survival models were used to investigate the association between per 5 kg/m^2^ higher BMI and cancer risk as a function of attained age.

We performed sensitivity analyses restricted to individuals not in the Medical Birth Register or Military Conscription Register for potential and established obesity-related cancers. We applied the E-value method for potential obesity-related cancers to assess the robustness of observed associations in the presence of residual confounding.^16^

We also calculated sex-specific absolute risks by age, stratified by BMI categories, for potential obesity-related cancers separately and in combination with established obesity-related cancers using competing risk models.^17^ We used attained age as time metric, and death as competing event.

All analyses were performed using Stata 17.0 (StataCorp LLC, College Station, TX).

### Role of the funding source

The funders of the study had no role in study design, data collection, data analysis, data interpretation, writing of the paper, or decision to submit the paper for publication.

## Results

A total of 4,142,349 individuals, 2,129,149 men and 2,013,200 women, with a mean baseline age of 23.1 (SD = 11.8) and 31.3 (SD = 9.7) years, were eligible for analysis. The mean BMI in men was 22.5 (SD = 3.3) kg/m^2^ and in women 24.0 (SD = 4.2) kg/m^2^, and obesity was prevalent in 3% (n = 69,385) of men and 9% (n = 176,650) of women. Characteristics of the total population by BMI category are shown in Table 1, and separately in men and women in the Appendix (pp 9–12). After a total of 100.2 million person-years and a median follow-up of 24.2 years (IQR = 13.8–34.7), 332,501 cancer cases, 192,816 in men and 139,685 in women, had been recorded. The median (IQR) age at first cancer diagnosis was 63.1 (52.8–71.9) years in men and 55.7 (46.1–66.5) years in women.

**Table 1:** Characteristics of the study population according to categories of body mass index and in total.

Characteristics	Underweight (BMI <18.5 kg/m^2^)	Normal weight (BMI 18.5–24.9 kg/m^2^)	Overweight (BMI 25–29.9 kg/m^2^)	Obesity (BMI ≥30 kg/m^2^)	Total
**N (%)**^a^	205,417 (5%)	2,912,814 (70%)	778,083 (19%)	246,035 (6%)	4,142,349 (100%)
**Person-years from baseline to end of follow-up**					
Median (IQR)	30.8 (18.7–39.1)	26.0 (15.1–35.7)	19.8 (11.0–29.6)	16.0 (8.0–23.8)	24.2 (13.8–34.7)
Total person-years included (million)	5.9	73.9	16.2	4.2	100.2
**Cohort (year of baseline examination), n (%)**					
Military conscription (1969–2014)	138,068 (67%)	1,390,137 (48%)	18,481 (3%)	41,876 (17%)	1,754,896 (42%)
Medical Birth Register (1982–2019)	56,597 (28%)	1,149,861 (39%)	372,086 (61%)	149,567 (61%)	1,728,111 (42%)
Other cohorts (1963–2019)	10,752 (5%)	372,816 (13%)	221,192 (36%)	54,592 (22%)	659,342 (16%)
**Sex, n (%)**					
Men	141,163 (69%)	1,588,998 (55%)	329,603 (42%)	69,385 (28%)	2,129,149 (51%)
Women	64,254 (31%)	1,323,816 (45%)	448,480 (58%)	176,650 (72%)	2,013,200 (49%)
**Baseline year, median (IQR)**	1987 (1977–1998)	1991 (1982–2001)	1997 (1986–2006)	2001 (1992–2010)	1992 (1983–2003)
Men	1983 (1975–1992)	1985 (1976–1994)	1987 (1975–1997)	1992 (1980–1999)	1985 (1976–1995)
Women	1997 (1986–2009)	1999 (1988–2008)	2002 (1993–2010)	2005 (1997–2012)	2000 (1989–2009)
**Baseline age, years**					
Mean (SD)	21.6 (6.8)	25.6 (10.3)	32.5 (14.1)	32.5 (13.2)	27.1 (11.6)
Category, n (%)					
<20	141,525 (69%)	1,413,882 (48%)	189,809 (24%)	44,799 (18%)	1,790,015 (43%)
20–29	42,927 (21%)	773,692 (27%)	228,136 (30%)	85,640 (35%)	1,130,395 (27%)
30–39	16,508 (8%)	493,061 (17%)	180,228 (23%)	64,985 (26%)	754,782 (18%)
≥40	4457 (2%)	232,179 (8%)	179,910 (23%)	50,611 (21%)	467,157 (12%)
**Weight measurement, n (%)**					
Objectively measured	199,656 (97%)	2,765,153 (95%)	699,353 (90%)	224,310 (91%)	3,888,472 (94%)
Self-reported	5761 (3%)	147,661 (5%)	78,730 (10%)	21,725 (9%)	253,877 (6%)
**Height measurement, n (%)**					
Objectively measured	161,846 (79%)	1,931,807 (66%)	385,558 (50%)	85,968 (35%)	2,565,179 (62%)
Self-reported	43,571 (21%)	981,007 (34%)	392,525 (50%)	160,067 (65%)	1,577,170 (38%)
**Baseline smoking status, n (%)**^d^					
Never	12,865 (51%)	265,211 (51%)	93,753 (45%)	22,606 (46%)	394,435 (49%)
Former	4084 (16%)	95,349 (18%)	50,868 (24%)	13,234 (27%)	163,535 (20%)
Current	8532 (33%)	157,773 (31%)	65,070 (31%)	13,498 (27%)	244,873 (31%)
**Highest achieved education, n (%)**^b^^,^^d^					
Pre-upper secondary school education <9 years	5445 (3%)	137,414 (5%)	99,287 (13%)	27,679 (12%)	269,825 (7%)
Pre-upper secondary school 9 years	25,105 (12%)	243,812 (9%)	62,594 (8%)	23,121 (10%)	354,632 (9%)
Upper secondary school <3 years	58,114 (29%)	704,385 (24%)	182,402 (24%)	56,633 (23%)	1,001,534 (24%)
Upper secondary school 3 years	40,150 (20%)	583,854 (20%)	166,919 (22%)	63,274 (26%)	854,197 (21%)
Post-upper secondary school <3 years	29,216 (14%)	436,261 (15%)	96,484 (12%)	28,779 (12%)	590,740 (14%)
Post-upper secondary school ≥3 years	45,330 (22%)	778,540 (27%)	156,939 (21%)	42,540 (17%)	1,023,349 (25%)
**Birth country for participant and parents, n (%)**^d^					
Born in Sweden, both parents born in Sweden	163,686 (80%)	2,334,581 (80%)	595,822 (76%)	179,829 (73%)	3,273,918 (79%)
Born in Sweden, one parent born in Sweden	15,853 (8%)	203,563 (7%)	44,764 (6%)	15,741 (7%)	279,921 (7%)
Born in Sweden, both parents born abroad	5244 (2%)	76,757 (3%)	20,431 (3%)	7777 (3%)	110,209 (3%)
Born abroad	20,615 (10%)	297,597 (10%)	116,951 (15%)	42,647 (17%)	477,810 (11%)
**Baseline marital status, n (%)**^c^^,^^d^					
Unmarried	171,546 (84%)	2,094,146 (72%)	417,869 (54%)	135,169 (55%)	2,818,730 (68%)
Married	30,799 (15%)	732,607 (25%)	313,268 (40%)	94,404 (39%)	1,171,078 (28%)
Divorced	2527 (1%)	71,038 (3%)	36,151 (5%)	12,832 (5%)	122,548 (3%)
Widow/-er	328 (0%)	12,058 (0%)	9667 (1%)	3290 (1%)	25,343 (1%)

Abbreviation: IQR, interquartile range.

a The percentages shown are row percentages, the rest in the table are column percentages.

b Highest achieved education through follow-up from the Population and Housing Census in 1970, and from the Longitudinal integration database for health insurance and labour market studies (LISA) in 1990 onwards.

c From the Population and Housing Census in 1960 and 1965, and from the Register of the Total Population in 1968 onwards. “Married”, “divorced” and “widow/-er” also include registered partners, recorded as of 1998.

d Number of individuals with missing values: highest achieved education, 48,072 (1%); birth country for participant and parents, 491 (<1%); marital status, 4650 (<1%). For smoking status, there were 2,189,850 (53%) individuals with a missing value, 1,119,226 (27%) individuals were recorded as never or former smoker, 30,430 (1%) individuals were current or former smoker.

Associations between BMI and all 122 cancers and cancer subtypes analysed in the study are shown in the Appendix (pp 13–21). Obesity as compared to normal weight or per 5 kg/m^2^ higher BMI was associated with an increased risk of many cancers not previously established as obesity related, specifically 15 cancers in men and 16 in women (18 altogether), accounting for approximately 15% of all cancer cases in the study (35,688 in men and 16,002 in women). The decision algorithm, defining a cancer as potentially obesity related, including in the presence of a sex-interaction and/or cancer heterogeneity, is illustrated in Fig. 1 and is shown in detail for each cancer in the Appendix (pp 22–24). HRs of potential obesity-related cancers for obesity vs. normal weight and per 5 kg/m^2^ higher BMI are shown in Table 2. These were cancers of the oral cavity, nasal and paranasal sinuses, gastric (gastrointestinal stromal tumours), small intestine, biliary tract, pancreatic islets, adrenal glands, parathyroid gland, pituitary gland, connective tissue, lymphoid neoplasms, and myeloid neoplasms for both men and women, and also included cancers of the head and neck (adenocarcinoma), penis, and malignant melanoma for men, and cancers of the head and neck (squamous-cell carcinoma), nodular melanoma, vulva, and cervix (adenocarcinoma) for women (Fig. 1, Table 2). Sex interactions were observed for cancers of the lip, tongue, head and neck (adenocarcinoma and squamous-cell carcinoma), malignant melanoma, connective tissue, and lymphoid neoplasms (Table 2). For some potential obesity-related cancers, specific cancer subtype/-s showed a stronger association with BMI than other subtypes (P value for heterogeneity in HRs <0.05). This was found for gastric gastrointestinal stromal tumours, small intestinal neuroendocrine tumours, cervical adenocarcinoma, diffuse large B-cell lymphoma in men, and nodular lymphocyte-predominant Hodgkin lymphoma.

**Table 2:** Hazard ratios (95% confidence intervals) of potential obesity-related cancers, and subtypes of established obesity-related cancers associated with body mass index in this study (*in italics*), according to body mass index and sex.^a^

Cancer category	All			Men			Women		
	No. at risk/cases	Obesity vs. normal weight^a^	Per 5 kg/m^2^ higher BMI^a^	No. at risk/cases	Obesity vs. normal weight^a^	Per 5 kg/m^2^ higher BMI^a^	No. at risk/cases	Obesity vs. normal weight^a^	Per 5 kg/m^2^ higher BMI^a^
Head and neck									
Oral cavity^d^	4,142,349/2688	1.11 (0.94–1.32)	1.06 (1.00–1.12)	2,129,149/1890	1.06 (0.85–1.33)	1.03 (0.96–1.11)	2,013,200/798	1.20 (0.92–1.57)	1.11 (1.01-1.21)
Lip	4,142,349/760	1.17 (0.87–1.57)	1.13 (1.01-1.25)^e^	2,129,149/597	1.22 (0.88–1.71)	1.17 (1.03–1.32)	2,013,200/163	NA^c^	1.06 (0.86–1.31)
Tongue	4,142,349/925	1.45 (1.09–1.91)^e^	1.14 (1.04–1.26)	2,129,149/627	1.29 (0.86–1.92)	1.09 (0.96–1.25)	2,013,200/298	1.66 (1.12–2.46)	1.19 (1.04–1.37)
Nasal and paranasal sinuses	4,142,349/431	1.55 (1.06–2.26)	1.16 (1.01–1.33)	2,129,149/293	1.75 (1.07–2.86)	1.19 (1.00–1.43)	2,013,200/138	NA^c^	1.12 (0.90–1.39)
Adenocarcinoma	4,042,179/208	NA^c^	1.26 (1.04–1.52)^e^	2,050,206/118	NA^c^	1.53 (1.18–1.98)	1,991,973/90	NA^c^	NA^c^
Squamous-cell carcinoma	4,142,349/6410	0.96 (0.85–1.08)^e^	0.96 (0.93–1.00)^e^	2,129,149/5062	0.86 (0.74–1.01)	0.92 (0.88–0.96)	2,013,200/1348	1.21 (0.99–1.49)	1.08 (1.01-1.16)
Gastric									
Gastrointestinal stromal	4,142,349/227	NA^c^	1.29 (1.08–1.55)	2,129,149/129	NA^c^	1.19 (0.90–1.59)	2,013,200/98	NA^c^	NA^c^
Small intestine^d^	4,142,349/1431	1.55 (1.25–1.93)	1.26 (1.17–1.35)	2,129,149/960	1.62 (1.20–2.19)	1.32 (1.19–1.45)	2,013,200/471	1.42 (1.03–1.95)	1.18 (1.05–1.32)
Duodenum	4,142,349/291	1.04 (0.60–1.83)	1.16 (0.98–1.38)	2,129,149/189	NA^c^	1.31 (1.05–1.64)	2,013,200/102	NA^c^	0.99 (0.76–1.30)
Ileum	4,092,313/348	2.35 (1.59–3.47)	1.39 (1.21–1.60)	2,087,151/216	NA^c^	1.37 (1.11–1.68)	2,005,162/132	NA^c^	1.41 (1.17–1.71)
Neuroendocrine	4,142,349/778	2.04 (1.56–2.68)	1.39 (1.26–1.52)	2,129,149/514	1.94 (1.31–2.86)	1.38 (1.20–1.57)	2,013,200/264	2.05 (1.41–2.98)	1.38 (1.20–1.59)
Colon^d^									
*Proximal*	4,142,349/9542	1.33 (1.23–1.45)^e^	1.15 (1.11–1.18)^e^	2,129,149/5592	1.51 (1.35–1.69)	1.20 (1.15–1.25)	2,013,200/3950	1.19 (1.06–1.33)	1.10 (1.06–1.15)
*Distal*	4,142,349/7253	1.43 (1.30–1.57)^e^	1.16 (1.13–1.20)^e^	2,129,149/4605	1.65 (1.46–1.87)	1.26 (1.20–1.31)	2,013,200/2648	1.21 (1.05–1.39)	1.07 (1.01-1.12)
*Adenocarcinoma*	4,142,349/16,801	1.38 (1.30–1.46)^e^	1.16 (1.13–1.18)^e^	2,129,149/10,269	1.60 (1.47–1.74)	1.24 (1.20–1.27)	2,013,200/6532	1.17 (1.07–1.28)	1.08 (1.05–1.12)
*Neuroendocrine*	4,142,349/774	1.53 (1.14–2.06)	1.15 (1.05–1.27)	2,129,149/421	1.04 (0.58–1.87)	1.07 (0.92–1.26)	2,013,200/353	1.80 (1.27–2.55)	1.21 (1.07–1.37)
Rectum/anus	4,142,349/11,644	1.19 (1.10–1.29)^e^	1.08 (1.06–1.12)^e^	2,129,149/7880	1.26 (1.14–1.40)	1.11 (1.07–1.15)	2,013,200/3764	1.10 (0.97–1.24)	1.05 (1.01-1.10)
*Adenocarcinoma*	4,142,349/10,519	1.20 (1.10–1.30)	1.09 (1.06–1.13)	2,129,149/7364	1.28 (1.16–1.43)	1.11 (1.07–1.16)	2,013,200/3155	1.07 (0.94–1.23)	1.07 (1.02–1.12)
Biliary tract^b^	4,142,349/2002	1.48 (1.26–1.74)	1.24 (1.16–1.31)	2,129,149/1070	1.28 (0.98–1.67)	1.19 (1.08–1.31)	2,013,200/932	1.61 (1.31–1.98)	1.26 (1.16–1.36)
Extrahepatic bile ducts	4,142,349/589	1.49 (1.07–2.08)	1.25 (1.11–1.41)	2,129,149/412	1.11 (0.69–1.80)	1.19 (1.02–1.39)	2,013,200/177	NA^c^	1.33 (1.12–1.59)
Malignant melanoma^f^	4,142,349/22,167	0.96 (0.89–1.02)^e^	1.05 (1.03–1.07)^e^	2,129,149/12,130	1.15 (1.04–1.28)	1.15 (1.11–1.18)	2,013,200/10,037	0.84 (0.77–0.92)	0.97 (0.95–1.00)
Acral lentiginous	4,042,179/310	1.04 (0.64–1.70)	1.08 (0.93–1.27)	2,050,206/135	NA^c^	1.29 (1.01-1.67)	1,991,973/175	NA^c^	0.97 (0.80–1.19)
Superficial spreading	4,042,179/12,855	0.91 (0.83–1.00)^e^	1.04 (1.02–1.07)^e^	2,050,206/6639	1.09 (0.94–1.27)	1.15 (1.10–1.20)	1,991,973/6216	0.83 (0.74–0.92)	0.97 (0.94–1.01)
Nodular	4,042,179/2281	1.09 (0.89–1.33)	1.10 (1.04–1.17)	2,050,206/1431	1.00 (0.75–1.35)	1.13 (1.03–1.23)	1,991,973/850	1.17 (0.90–1.53)	1.08 (0.99–1.18)
Vulva	–	–	–	–	–	–	2,013,200/597	2.43 (1.88–3.14)	1.42 (1.29–1.55)
Cervix									
Adenocarcinoma							2,013,200/1082	1.34 (1.08–1.65)	1.10 (1.02–1.19)
Endometrium^d^									
*Type I*	–	–	–	–	–	–	1,991,973/2602	3.35 (2.99–3.76)	1.68 (1.61–1.74)
*Type II*	–	–	–	–	–	–	1,991,973/167	–	1.54 (1.30–1.83)
Penis	–	–	–	2,129,149/626	3.07 (2.28–4.14)	1.54 (1.38–1.73)	–	–	–
Renal cell^d^									
*Clear cell*	3,837,868/2962	2.39 (2.07–2.76)	1.53 (1.46–1.61)	1,911,072/2127	2.39 (1.96–2.90)	1.54 (1.45–1.64)	1,926,796/835	2.38 (1.92–2.97)	1.52 (1.41–1.64)
*Papillary*	4,042,179/466	1.56 (1.02-2.40)	1.25 (1.09–1.43)	2,050,206/367	1.20 (0.66–2.17)	1.19 (1.00–1.41)	1,991,973/99	NA^c^	NA^c^
*Chromophobe*	3,837,868/282	2.06 (1.31–3.24)	1.31 (1.12–1.54)	1,911,072/162	NA^c^	1.40 (1.11–1.77)	1,926,796/120	NA^c^	1.24 (1.00–1.54)
Endocrine organs	4,142,349/11,224	1.34 (1.24–1.45)	1.14 (1.11–1.17)^e^	2,129,149/4470	1.52 (1.31–1.77)	1.21 (1.16–1.27)	2,013,200/6754	1.28 (1.18–1.40)	1.11 (1.08–1.15)
Pancreatic islets	4,142,349/480	1.36 (0.90–2.05)	1.20 (1.06–1.37)	2,129,149/292	1.65 (0.90–3.00)	1.40 (1.18–1.66)	2,013,200/188	NA^c^	1.03 (0.86–1.25)
Thyroid^d^									
*Papillary*	4,042,179/2809	1.24 (1.07–1.43)	1.08 (1.03–1.14)^e^	2,050,206/703	2.16 (1.56–2.99)	1.21 (1.08–1.36)	1,991,973/2106	1.12 (0.95–1.31)	1.06 (1.00–1.12)
*Follicular*	4,042,179/408	1.20 (0.81–1.78)	1.17 (1.03–1.32)	2,050,206/107	NA^c^	1.30 (0.98–1.74)	1,991,973/301	1.25 (0.83–1.91)	1.15 (1.00–1.32)
Adrenal glands	4,142,349/479	1.50 (1.03–2.17)	1.18 (1.04–1.33)	2,129,149/249	NA^c^	1.13 (0.92–1.40)	2,013,200/230	NA^c^	1.19 (1.02–1.40)
Parathyroid gland	4,142,349/3144	1.41 (1.23–1.62)	1.16 (1.11–1.22)	2,129,149/976	1.40 (1.02–1.93)	1.21 (1.09–1.33)	2,013,200/2168	1.40 (1.20–1.63)	1.15 (1.09–1.21)
Pituitary gland	4,142,349/2936	1.57 (1.35–1.83)	1.19 (1.13–1.25)	2,129,149/1662	1.63 (1.27–2.09)	1.25 (1.16–1.35)	2,013,200/1274	1.54 (1.27–1.86)	1.16 (1.08–1.24)
Connective tissue	4,142,349/2029	1.34 (1.11–1.62)	1.20 (1.13–1.28)^e^	2,129,149/1364	1.59 (1.23–2.04)	1.28 (1.17–1.38)	2,013,200/665	1.10 (0.82–1.47)	1.11 (1.01-1.23)
Lymphoid neoplasms^d^	4,142,349/16,018	1.22 (1.14–1.31)	1.11 (1.09–1.14)^e^	2,129,149/11,225	1.30 (1.19–1.43)	1.14 (1.11–1.18)	2,013,200/4793	1.12 (1.01-1.25)	1.08 (1.04–1.12)
Hodgkin lymphoma^d^	4,142,349/2239	1.38 (1.13–1.67)	1.18 (1.11–1.25)	2,129,149/1653	1.52 (1.18–1.96)	1.20 (1.12–1.29)	2,013,200/586	1.23 (0.91–1.66)	1.15 (1.04–1.26)
Mixed cellularity	4,042,179/237	NA^c^	1.30 (1.10–1.54)	2,050,206/182	NA^c^	1.33 (1.08–1.63)	1,991,973/55	NA^c^	NA^c^
Nodular lymphocyte	4,042,179/166	NA^c^	1.72 (1.45–2.03)	2,050,206/128	NA^c^	1.53 (1.21–1.94)	1,991,973/38	NA^c^	NA^c^
Acute lymphocytic leukaemia	4,142,349/539	1.61 (1.13–2.29)	1.18 (1.05–1.33)	2,129,149/379	1.69 (1.04–2.75)	1.20 (1.03–1.40)	2,013,200/160	NA^c^	1.17 (0.97–1.41)
Chronic lymphocytic leukaemia	4,142,349/2854	1.05 (0.89–1.24)	1.06 (1.00–1.12)	2,129,149/2116	1.13 (0.93–1.39)	1.08 (1.01–1.16)	2,013,200/738	0.90 (0.66–1.21)	1.01 (0.91–1.12)
Diffuse large B-cell	4,042,179/2775	1.53 (1.31–1.79)	1.23 (1.17–1.30)	2,050,206/1818	1.70 (1.37–2.10)	1.29 (1.20–1.39)	1,991,973/957	1.37 (1.08–1.72)	1.16 (1.07–1.26)
Follicular	4,042,179/1931	1.15 (0.94–1.41)	1.09 (1.02–1.16)	2,050,206/1111	1.42 (1.07–1.90)	1.14 (1.03–1.25)	1,991,973/820	0.98 (0.74–1.30)	1.04 (0.95–1.14)
Myeloid neoplasm^d^	4,142,349/4784	1.41 (1.26–1.59)	1.15 (1.10–1.20)	2,129,149/3101	1.44 (1.22–1.70)	1.16 (1.09–1.22)	2,013,200/1683	1.39 (1.17–1.64)	1.14 (1.07–1.21)
Acute myeloid leukaemia	4,142,349/1498	1.24 (1.00–1.54)	1.13 (1.05–1.22)	2,129,149/961	1.36 (1.02–1.81)	1.14 (1.03–1.27)	2,013,200/537	1.13 (0.83–1.56)	1.12 (1.01-1.25)
Chronic myeloid leukaemia	4,142,349/1002	1.47 (1.13–1.92)	1.17 (1.07–1.28)	2,129,149/679	1.42 (0.97–2.08)	1.16 (1.03–1.31)	2,013,200/323	1.55 (1.06–2.27)	1.19 (1.04–1.36)

Abbreviations: NW, normal weight; BMI, body mass index.

a Hazard ratios from Cox regression models with age as time scale, adjusted for baseline age (continuous), weight assessment from the Medical Birth Register (yes/no), mode of weight assessment, mode of height assessment, marital status, education level, and birth country, and stratified by sex (in analysis of men and women combined) and calendar year of birth.

b Gallbladder cancer, which is established obesity-related, is a subtypes of biliary tract cancer and makes up around half of biliary tract cancer.

c The number of cancer cases was considered too low for analysis (<250 cases for categorical body mass index and <100 cases for per 5 kg/m^2^).

d The heterogeneity of hazard ratios per 5 kg/m^2^ higher BMI between cancer subtypes was calculated using the Lunn and McNeil duplication method, separately in men and women whenever a sex-interaction was identified. P = 0.0039 for the groups of gastric-adenocarcinoma, gastric-neuroendocrine, and gastric-gastrointestinal stromal; P = 0.0045 for the groups of small intestine-adenocarcinoma, small intestine-neuroendocrine, and small intestine-gastrointestinal stromal; P = 0.00030 for cervix-adenocarcinoma vs. cervix-squamous-cell carcinoma; P = 0.0019 for the groups of renal cell-clear cell, renal cell-papillary, and renal cell-chromophobe; P < 0.0001 for the groups of Hodgkin-nodular sclerosis, Hodgkin-mixed cellularity, and Hodgkin-nodular lymphocyte; P < 0.0001 for the groups of Hodgkin lymphoma, acute lymphocytic leukaemia, chronic lymphocytic leukaemia, diffuse large B-cell lymphoma, follicular lymphoma, and T-cell/natural killer-cell lymphoma in men. No heterogeneity was found between other cancer subtypes.

e P _sex-interaction_<0.05, calculated by adding a product term of sex and BMI in categories or per 5 kg/m^2^ higher BMI in the Cox model using Wald test.

f Hazard ratios for malignant melanoma and its subtypes were also calculated additionally adjusted for height (continuous) — a strong risk factor and commonly adjusted for in studies of BMI and malignant melanoma risk. The associations did not change, for example, HRs (95% CI) per 5 kg/m^2^ higher BMI for malignant melanoma, melanoma-superficial spreading, and melanoma-nodular were 1.06 (1.04–1.08), 1.06 (1.03–1.08), and 1.11 (1.05–1.18), respectively.

Among potential obesity-related cancers that we defined as smoking-related (HR for current vs. never smokers above 1.5, Appendix p 8), the association with BMI remained for cancers of the oral cavity, tongue, and head and neck (adenocarcinoma) but disappeared for nasal and paranasal sinuses cancer and Hodgkin lymphoma after adjusting for smoking status and/or among never-smokers using data from 802,843 individuals with smoking information available (Appendix pp 25 and 26). Due to the lack of data on other potential confounders than smoking, we reported E-values alongside HRs of potential obesity-related cancers (Appendix p 27). For a larger E-value, such as for cancers of the penis and pituitary gland, it is less likely that an unmeasured confounder could fully explain the observed association with obesity.

We confirmed associations between obesity and/or per 5 kg/m^2^ higher BMI and higher risks of all established obesity-related cancers; these accounted for approximately 25% of all cancer cases in the study (36,462 in men and 47,922 in women, Appendix pp 13–21). For renal cell carcinoma morphological subtypes, the strongest association was observed with clear cell carcinoma (P value for heterogeneity <0.05, Table 2). No heterogeneity of associations was observed for subtypes of cancers of the colon, rectum, thyroid, endometrium, and ovary.

**Fig. 2:**
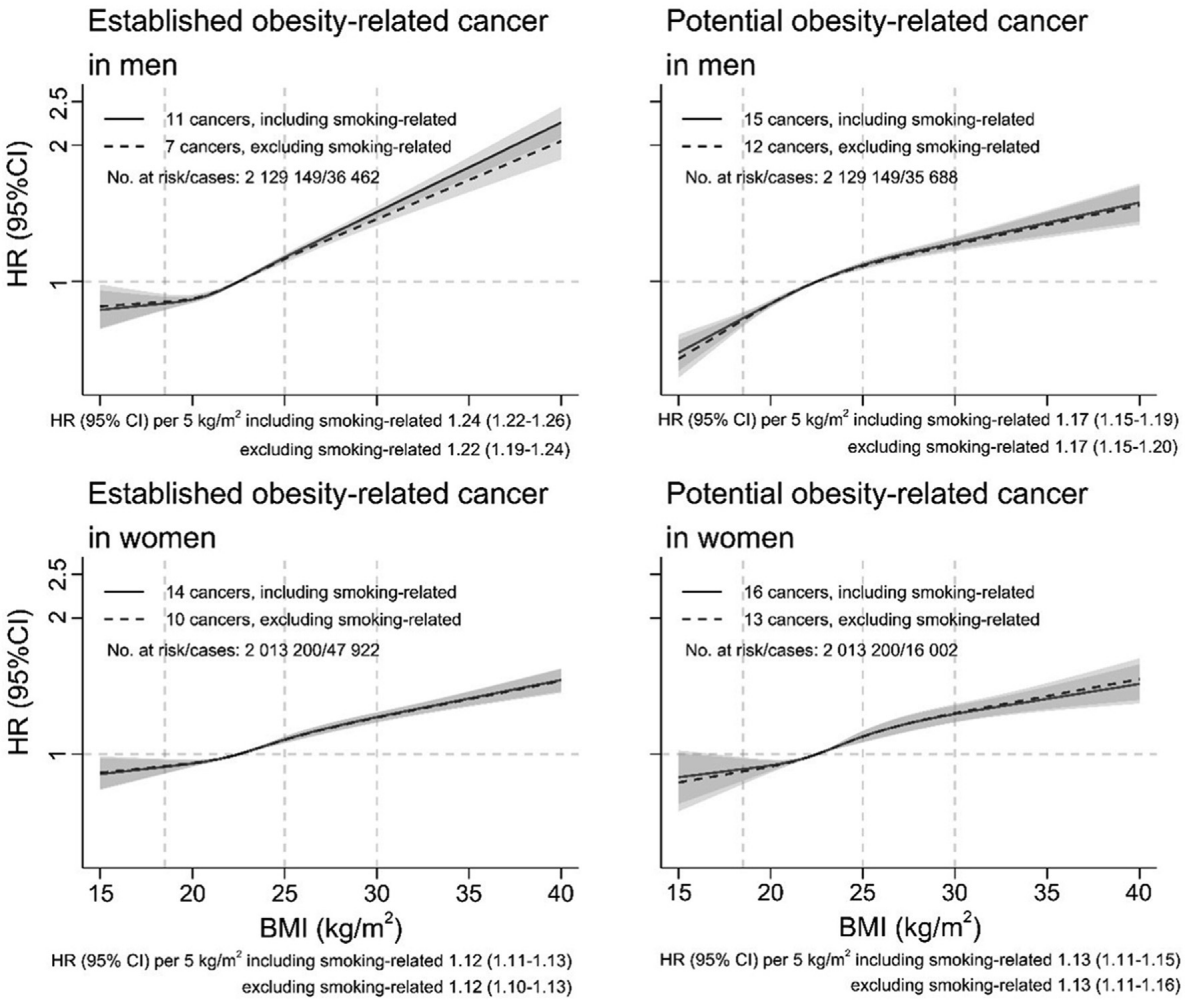
Hazard ratios of established obesity-related cancers and potential obesity-related cancers according to BMI allowing for non-linear associations, with 95% confidence intervals. The reference BMI was 22.5 kg/m^2^. Restricted cubic splines for BMI with knots placed at Harrell's recommended percentiles of BMI were fitted adjusting for baseline age (continuous), weight assessment from Medical Birth Register, mode of weight assessment, mode of height assessment, marital status, education level, and birth country, and stratified by calendar year of birth. Smoking-related cancers include cancers of the oral cavity, nasal and paranasal sinuses, head and neck (adenocarcinoma), and head and neck (squamous cell carcinoma), oesophagus (adenocarcinoma), stomach-cardia, liver/intrahepatic bile ducts, and pancreas. HR, hazard ratio; CI, confidence interval; BMI, body mass index.

The HR of all potential obesity-related cancers per 5 kg/m^2^ higher BMI was 1.17 (95% CI 1.15–1.19) in men and 1.13 (95% CI 1.11–1.15) in women (Fig. 2). In comparison, the association with all established obesity-related cancers was slightly stronger in men (HR 1.24, 95% CI 1.22–1.26), but of similar effect size in women (HR 1.12, 95% CI 1.11–1.13). After excluding individuals in the Medical Birth Register and the Military Conscription Register, which dominated the full population, effect sizes for potential and established obesity-related cancers, respectively, were similar to the results of all individuals (Appendix p 28).

For each investigated cancer in the study, the shape of the association with BMI, allowing for non-linearity, is shown in the Appendix (pp 30 and 31), and sex-specific associations are shown in the Appendix (p 32) for cancers with a sex interaction (P value < 0.05). Among potential obesity-related cancers with at least 250 cases, non-linear associations with BMI were found for cancers of the biliary tract, malignant melanoma, vulva, and pituitary gland (Appendix pp 30 and 31). The Appendix (p 33) shows HRs (per 5 kg/m^2^ higher BMI) as a function of attained age for a few cancers where BMI appeared to violate the proportional hazards assumption.

**Fig. 3:**
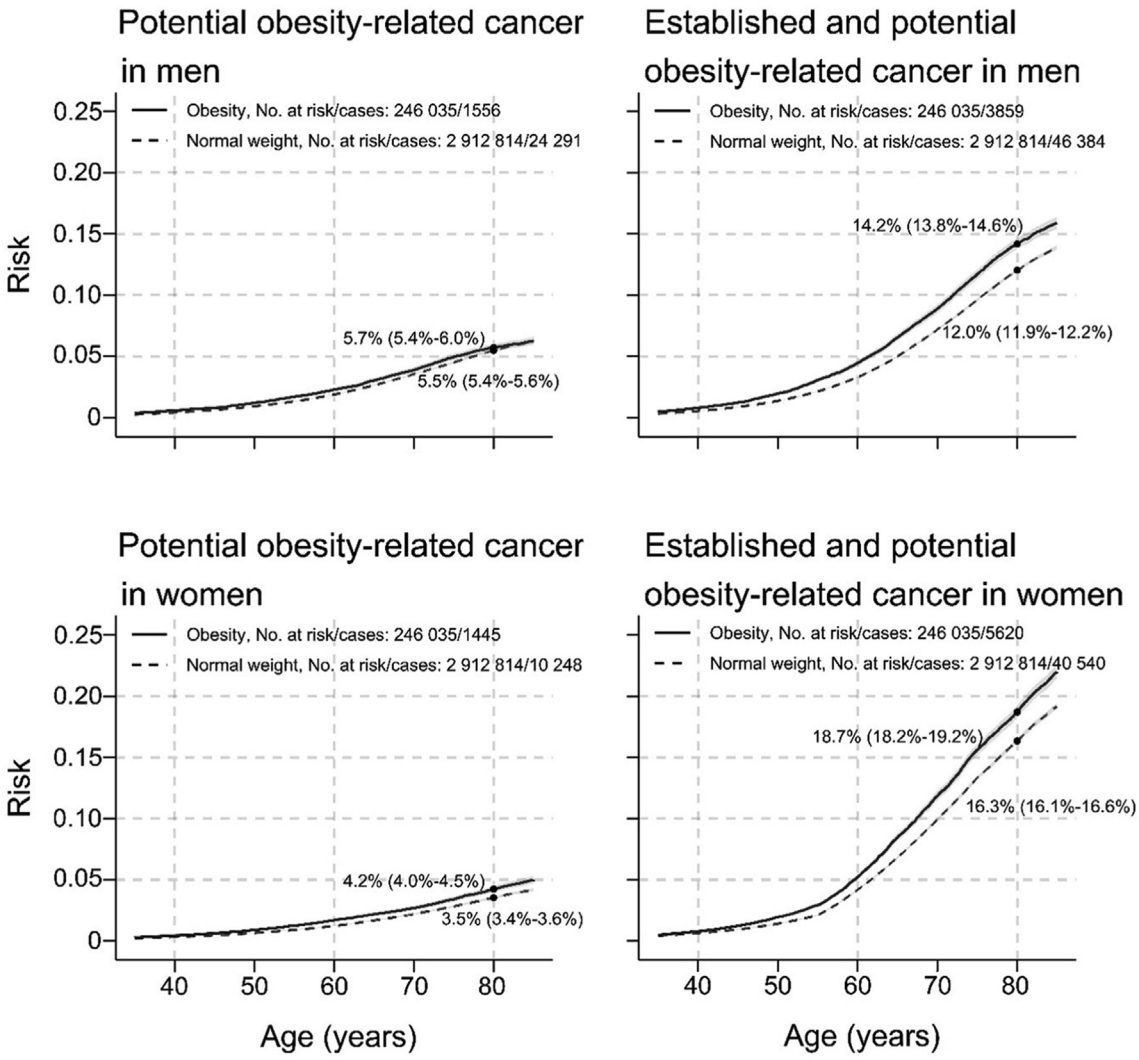
Risk of potential obesity-related cancers separately and in combination with established obesity-related cancers, in men and women with obesity and normal weight, respectively. Cumulative risks were calculated using age as time metric and death as competing event. Shaded areas are 95% confidence bands. HR, hazard ratio; CI, confidence interval; BMI, body mass index. Normal weight: 18.5 is BMI <25 kg/m^2^; obesity: BMI ≥30 kg/m^2^.

The absolute risk of potential obesity-related cancers from age 35 to 85 years, separately and in combination with established obesity-related cancers, is shown in Fig. 3 for normal weight and obesity. In men, the risk of a potential obesity-related cancer by age 80 was 5.5% (5.4%–5.6%) for normal weight and 5.7% (5.4%–6.0%) for obesity. In women, the corresponding risks were 3.5% (3.4%–3.6%) and 4.2% (4.0%–4.5%). For potential and established obesity-related cancers combined, these risks increased to 12.0% (11.9%–12.2%) and 14.2% (13.8%–14.6%) in men and 16.3% (16.1%–16.5%) and 18.7% (18.1%–19.1%) in women, respectively.

## Discussion

In this large, pooled cohort study, we identified 15 cancers in men and 16 in women (18 altogether), previously not established obesity related, as potential obesity-related cancers. These were cancers of the head and neck, gastrointestinal tract, malignant melanoma, genital organs, endocrine organs, connective tissue, and haematological malignancies. The association of BMI with the risk of potential obesity-related cancers overall was somewhat weaker than that of established obesity-related cancers in men but was similar in women.

There is a large body of observational studies on BMI and cancer risk, but these have mostly focused on more common site-specific cancers. For example, two studies were based on millions of adults in the UK and Spain, respectively, with more than 200,000 incident cancer cases, categorised into 22 and 26 of the most common cancers.^5^^,^^6^ The inconclusive evidence on obesity and the risk of many cancers remains, as they are either rare or only weakly associated with BMI. To establish an association with these, a large number of cases and a detailed cancer categorisation are needed—in our study with over 330,000 incident cancer cases, we were able to investigate more than 100 cancer outcomes. Our findings strengthen the evidence for a range of cancers previously reported by the IARC,^2^ the World Cancer Research Fund (WCRF),^18^ or an umbrella review from 2017,^3^ as having a potential association with obesity, but with insufficient evidence. These include malignant melanoma (men only), diffuse large B-cell lymphoma, other lymphoid neoplasms, myeloid neoplasms (the IARC report used non-Hodgkin lymphoma and leukaemia instead, but the included subtypes largely overlap), and cancers of the oral cavity, cervix (adenocarcinoma only), and extrahepatic bile duct. For other cancers reported as potentially obesity related in previous reports, for example, male breast cancer, testicular cancer, and glioma, we found no association. The WCRF suggested advanced prostate cancer to be obesity related,^18^ but we, another recent large observational study,^19^ and Mendelian randomisation studies,^20^ found no support.

We also found positive associations between BMI and the risk of a range of cancers for which prior investigations are few or non-existent, including cancers of the small intestine, vulva, penis, some endocrine organs, connective tissue, and gastric gastrointestinal stromal tumours. Among these, the strongest association was found for penile cancer, for which the risk doubled among men with obesity relative to normal weight. It has been hypothesised that such an association may be mediated by poor hygiene and impaired self-examination in individuals with severe obesity.^21^ A range of rare malignant endocrine neoplasms, including cancers of the pancreatic islets, adrenal glands, parathyroid gland, and pituitary gland, were found to be positively associated with BMI. As these organs secrete hormones, it is biologically plausible that excess weight causes hormonal changes promoting cancer, but confirmatory epidemiological and experimental studies are needed. The positive association found between BMI and gastrointestinal stromal tumours risk has rarely been reported, as previous studies mostly classified gastric cancer based on the cancer site rather than on morphology. For cancer of the small intestine, some studies have suggested a positive association between BMI and neuroendocrine tumours, but not with adenocarcinoma,^22^^,^^23^ which is consistent with our results. Connective and soft tissue tumours, known as soft-tissue sarcoma, were positively associated with BMI in a case–control study which is consistent with our finding^24^; but few studies have addressed this rare and heterogeneous disease. Radiation and certain chemicals are some of the proposed risk factors, and most of these chemicals accumulate in human adipose tissue.^25^^,^^26^

Our findings support a positive association between BMI and haematological malignancies in addition to multiple myeloma, which is an already established obesity-related cancer. We observed positive associations between BMI and risk of lymphoid and myeloid neoplasms, which has been observed previously for leukaemia and non-Hodgkin lymphoma,^27^, ^28^, ^29^ yet, the evidence so far has not been sufficient to conclude that these cancers are obesity related.^2^^,^^3^^,^^18^ The association between BMI and Hodgkin lymphoma has been less investigated. A recent meta-analysis of five studies showed a positive association particularly in women^30^; but a sex difference was not observed in our study. It has been hypothesised that adipocytes may modify the bone marrow microenvironment and provide substances that could enhance the growth and survival of tumour cells.^31^ For haematological malignancies as well as for several other cancers, insulin resistance, insulin-like growth factors, and systemic inflammation are further potential mechanisms linking obesity to cancer.^32^

Our results on established obesity-related cancers are consistent with current evidence and provide further evidence for specific cancer subtypes driving some of the associations. For renal cell carcinoma, we confirmed the associations between obesity and clear cell and chromophobe subtypes as previously reported,^33^ and we also found an association with papillary carcinoma. Of these, clear cell carcinoma was most strongly associated with BMI. For thyroid cancer, the papillary subtype dominates; but we observed a positive association with BMI also for the rare follicular subtype. Concerning endometrial cancer, we found associations for both type I and type II tumours, and did not confirm the particularly pronounced risk for type I tumours reported by a Norwegian study.^34^

This study has some limitations. Most importantly, we have no information on many potential confounders, and the lack of adjustment for these bears the risk of residual confounding. Smoking is a strong potential confounder for several cancers. Analyses of around 800,000 individuals with information on smoking status indicated a persistent association with BMI for some, but not other, smoking-related cancers after smoking adjustment or analyses in never-smokers only. Examples of other potential cancer-specific confounders for which we did not control are reproductive factors (e.g., parity and age at first birth), virus infection history (e.g., human papillomavirus and Epstein–Barr virus), and lifestyle (e.g., diet, alcohol, and physical activity). The investigation of 122 cancers and cancer subtypes increases the potential of false positive findings (i.e., type I error). For example, for analyses of men and women combined, we found significant positive associations for 32 out of 78 cancer endpoints (excluding established obesity-related cancers and subtypes). A simple calculation shows that around four of these are expected to be false positives, and that the likelihood of more than seven false positives is less than 5%. This means that most positive associations are likely to be true (Appendix pp 12–14). Still, all findings should be interpreted as exploratory, and replication in future studies is warranted.

This study also has several strengths. Firstly, the large sample size and long follow-up gave our investigation high statistical power, and, together with the detailed cancer categorisation, also enabled the investigation of rarer cancers and cancer subtypes. We applied a consistent methodological approach to estimate associations for each cancer, and a systematic process to define potential obesity-related cancers. Representativeness of the background population is high for the two largest cohorts in the study, i.e., military conscripts and women in their early pregnancy, and the associations with obesity-related cancers in these cohorts were similar to those of the other cohorts combined, demonstrating high robustness of our findings across populations in Sweden.

The findings of this study have important public health implications. Established obesity-related cancers accounted for 25% of all cancer cases in this study, and the proportion increased to 40% when potential obesity-related cancers were added. Therefore, a substantial proportion of cancers could potentially be prevented by implementing public health measures enabling and advocating a healthy lifestyle to keep a normal weight, or to reduce weight with the same measures or by obesity treatment. Cancer risk has been shown to reduce after bariatric surgery of individuals with obesity, which could be an effect of the resulting weight loss.^35^ Nevertheless, our findings, particularly of rarer cancers, should be verified in future studies and in updated systematic reports weighing the total epidemiological and biological mechanistic evidence to conclude which cancers are likely to be caused by obesity.

## Contributors

MS and TS were involved in the conception and design of the study. JW, KM, BVG, PM, SS, WY, YTL, WY, BN, HK, LL, AC, KI, NP, SE, LL, and LH contributed original cohort data. MdS harmonized the cohort data. JF and TS harmonized register data. MS analysed data. MS and TS drafted the manuscript. MS, MdS, TB, JF, IBM, MJ, CH, and TS revised the manuscript. All authors made significant contributions to the manuscript and have read and approved the final version.

## Data sharing statement

All data are located on Statistics Sweden's Microdata Online Access (MONA) server and may only be accessed from countries in the European Union or the European Economic Area. Data access covered by the ethical approval will be considered in agreement with the principal investigator of ODDS, Tanja Stocks, and upon approval from register holders and steering committees of ODDS cohorts.

## Declaration of interests

We declare no competing interests.
