# Supplementary material for: Body mass index and risk of over 100 cancer forms and subtypes in 4.1 million individuals in Sweden: the Obesity and Disease Development Sweden (ODDS) pooled cohort study
Source: Lancet Reg Health Eur. 2024 Aug 20;45:101034. doi: 10.1016/j.lanepe.2024.101034 (PMC11381908; doi:10.1016/j.lanepe.2024.101034)
Supplement: Supplementary appendix [file mmc1.docx]

**Supplementary Appendix Contents**

Supplementary Tables2

Table S1. The codes according to the International Classification of Diseases, the International Classification of Diseases for Oncology, and WHO/HS/CANC/24·1 (Swedish PAD codes) used to categorise cancer in the study 2

Table S2. The combination of the International Classification of Diseases, 7th edition (ICD7) or ICD10 and/or Swedish PAD codes/C24.1 (histopathology) that were exempted from the rule to define a tumour as benign if the third digit of C24.1 was not 6 5

Table S3. Number of individuals in each cohort 6

Table S4. Hazard ratios (95% confidence intervals) of height per 5 cm, and per 5 kg/m^2^ higher body mass index in height unadjusted and adjusted analyses of all individuals 7

Table S5. Hazard ratios of site-specific cancers for current vs. never smokers in individuals with smoking information available 8

Table S6. Characteristics of men in the study according to categories of body mass index and in total 9

Table S7. Characteristics of women in the study according to categories of body mass index and in total 11

Table S8. Hazard ratios (95% confidence intervals) of site-specific cancers according to body mass index level in men and women combined 13

Table S9. Hazard ratios (95% confidence intervals) of site-specific cancers according to body mass index level in men 16

Table S10. Hazard ratios (95% confidence intervals) of site-specific cancers according to body mass index level in women 19

Table S11. Summary of findings for each cancer and its inclusion or exclusion as a potential obesity-related cancer 22

Table S12. Hazard ratios (95% confidence intervals) of smoking-related cancers according to body mass index level with results shown for smoking unadjusted analysis of all individuals, smoking unadjusted and adjusted analysis of individuals with smoking information, and for never smokers 25

Table S13. Hazard ratios (95% confidence interval) and e-values for point estimates and lower confidence limits of hazard ratios of potential obesity-related cancers associated with an increased risk for obesity vs. normal weight in the study 27

Table S14. Hazard ratios (95% confidence interval) of established obesity-related cancers and potential obesity-related cancers according to body mass index level, in all individuals and with the exclusion of the Medical Birth Register and military conscription 28

Supplementary Figures29

Figure S1. Flowchart of exclusions and selections of individuals and observations in the study 29

Figure S2. Hazard ratios of site-specific cancers according to BMI, allowing for non-linear effects, with 95% confidence intervals 30

Figure S3. Hazard ratios of cancers separately for men (solid line) and women (dashed line), for cancers with a differential association by sex according to BMI, allowing for non-linear effects, with 95% confidence intervals32

Figure S4. Age-dependent hazard ratios with 95% confidence intervals for cancers for which the proportional hazards assumption was indicated to be violated per 5 kg/m² higher body mass index 33

**Table S1. The codes according to the International Classification of Diseases, the International Classification of Diseases for Oncology, and WHO/HS/CANC/24·1 (Swedish PAD codes) used to categorise cancer in the study**^*^

| Cancer category | | | ICD7  (from 1958,  100% coverage) | ICD9  (from 1987, 92% coverage) | ICD10  (from 1993,  87% coverage) | ICD-O-2  (from 1993,  87% coverage) | ICD-O-3  (from 2005, 66% coverage) | C24.1  (from 1958, 100% coverage) |
| --- | --- | --- | --- | --- | --- | --- | --- | --- |
| Head and neck | | | 140,141.0, 141.7-9, 142-144, 145.0, 145.7-9, 146-148, 160-161 |  | C00, C01.9, C02-04, C05-14, C30-C32 |  |  |  |
|  | Oral cavity | | 140, 141.7-9,143-144 |  | C00, C02-04, C05.0, C06 |  |  |  |
|  |  | Lip | 140 |  | C00 |  |  |  |
|  |  | Tongue | 141.7-9 |  | C02 |  |  |  |
|  |  | Mouth | 143-144 |  | C03-04, C05.0, C06 |  |  |  |
|  | Salivary glands | | 142 |  | C07-08 |  |  |  |
|  | Pharynx | | 141.0, 145.0, 145.7-9, 146-147 |  | C01.9, C05.1-9, C09-13 |  |  |  |
|  |  | Oropharynx | 141.0, 145.0, 145.7-9 |  | C01.9, C05.1-9, C09-10 |  |  |  |
|  |  | Nasopharynx | 146 |  | C11 |  |  |  |
|  |  | Hypopharynx | 147 |  | C12-13 |  |  |  |
|  | Nasal and paranasal sinuses | | 160 |  | C30-31 |  |  |  |
|  | Larynx | | 161 |  | C32 |  |  |  |
|  | Adenoid cystic carcinoma | |  |  |  |  |  | 056 |
|  | Mucoepidermoid carcinoma | |  |  |  |  |  | 076 |
|  | Adenocarcinoma | |  |  |  | 81403 |  |  |
|  | Squamous-cell carcinoma | |  |  |  |  |  | 146 |
| Oesophagus | | | 150 |  | C15 |  |  |  |
|  | Squamous-cell carcinoma | |  |  |  |  |  | 096 |
|  | Adenocarcinoma | |  |  |  |  |  | 146 |
| Gastric | | | 151 |  | C16 |  |  |  |
|  | Cardia | | 151.1 |  | C16.0 |  |  |  |
|  | Non-cardia | | 151.0, 151.8-9 |  | C16.1-9 |  |  |  |
|  | Adenocarcinoma | |  |  |  |  |  | 096 |
|  | Neuroendocrine | |  |  |  |  | 82463 | 086 |
|  | Gastrointestinal stromal | |  |  |  |  |  | 796 |
| Small intestine | | | 152 |  | C17 |  |  |  |
|  | Duodenum | | 152.0 |  |  |  |  |  |
|  | Jejunum | |  | 152.1 |  |  |  |  |
|  | Ileum | |  | 152.2 |  |  |  |  |
|  | Adenocarcinoma | |  |  |  |  |  | 096 |
|  | Neuroendocrine | |  |  |  |  | 82463 | 086 |
|  | Gastrointestinal stromal | |  |  |  |  |  | 796 |
| Colon | | | 153 |  | C18 |  |  |  |
|  | Proximal | | 153.0-1, 153.4 |  | C18.0-5 |  |  |  |
|  | Distal | | 153.2-3 |  | C18.6-7 |  |  |  |
|  | Adenocarcinoma | |  |  |  |  |  | 096 |
|  | Neuroendocrine | |  |  |  |  | 82463 | 086 |
| Rectum/anus | | | 154 |  | C19-21 |  |  |  |
|  | Rectum | | 154.0 |  | C19-20 |  |  |  |
|  | Anus | | 154.1, 154.8 |  | C21 |  |  |  |
|  | Adenocarcinoma | |  |  |  |  |  | 096 |
|  | Neuroendocrine | |  |  |  |  | 82463 | 086 |
| Liver/intrahepatic bile ducts | | | 155.0, 156 |  | C22 |  |  |  |
| Biliary tract | | | 155.1-9 |  | C23-24 |  |  |  |
|  | Gallbladder | | 155.1 |  | C23 |  |  |  |
|  | Extrahepatic bile ducts | | 155.2 |  | C24.0 |  |  |  |
|  | Ampulla/papilla of Vater | | 155.3 |  | C24.1 |  |  |  |
| Pancreas | | | 157 |  | C25.0-3, C25.8-9 |  |  |  |
| Lung/bronchus | | | 162.1 |  | C34 |  |  |  |
|  | Adenocarcinoma | |  |  |  | 81403, 82503, 82603, 84803 |  | 076, 096 |
|  | Squamous-cell carcinoma | |  |  |  | 80703 |  | 146 |
|  | Small cell | |  |  |  | 80413, 80423, 80433 |  | 186 |
|  | Large cell | |  |  |  | 80123, 80823, 81233, 83103 |  |  |
| Malignant melanoma | | | 190 |  | C43 |  |  |  |
|  | Acral lentiginous | |  |  |  | 87443 |  |  |
|  | Superficial spreading | |  |  |  | 87433 |  |  |
|  | Nodular | |  |  |  | 87213 |  |  |
|  | Lentigo maligna | |  |  |  | 87423 |  |  |
| Non-melanoma excluding basalioma | | | 191 |  | C44 |  |  |  |
| Breast | | | 170 |  | C50 |  |  |  |
| Vulva | | | 176.0, 176.7-8 |  | C51 |  |  |  |
| Vagina | | | 176.1 |  | C52 |  |  |  |
| Cervix | | | 171 |  | C53 |  |  |  |
|  | Squamous-cell carcinoma | |  |  |  |  |  | 146 |
|  | Adenocarcinoma | |  |  |  |  |  | 096 |
| Uterus | | | 172, 174 |  | C54.0-1, C54.3, C54.9, C55 |  |  |  |
|  | Endometrium | |  |  | C54.1 |  |  |  |
|  |  | Type I tumours |  |  |  | 83803, 81403, 85603, 85703, 84803 |  |  |
|  |  | Type II tumours |  |  |  | 84413, 84603, 83103 |  |  |
| Ovary | | | 175.0 |  | C56 |  |  |  |
|  | Serous | |  |  |  | 84413, 84603 |  |  |
|  | Mucinous | |  |  |  | 84703, 84803 |  |  |
|  | Endometrioid | |  |  |  | 83803 |  |  |
|  | Clear cell | |  |  |  | 83103, 91103 |  |  |
| Penis | | | 179.0 |  | C60 |  |  |  |
| Prostate | | | 177 |  | C61 |  |  |  |
|  | Aggressive^#^ | | T4, N1, M1, Gleason score ≥ 8, or PSA ≥ 50 ng/mL (1998-) | | | | | |
|  | Non-aggressive | |  |  | Not aggressive (1998-) |  |  |  |
| Testis | | | 178 |  | C62 |  |  |  |
| Renal cell | | | 180.0 |  | C64.0 |  |  |  |
|  | Clear cell | |  |  |  |  | 83103 |  |
|  | Papillary | |  |  |  | 82603 | 82603 |  |
|  | Chromophobe | |  |  |  |  | 83173 |  |
| Other urinary tract | | | 180.1, 181.0-2, 181.6-9 |  | C65-68 |  |  |  |
|  | Pelvis | | 180.1 |  | C65 |  |  |  |
|  | Ureter | | 181.1 |  | C66 |  |  |  |
|  | Bladder | | 181.0, 181.6 |  | C67 |  |  |  |
|  | Urothelial carcinoma | |  |  |  |  | 81202, 81203, 812031, 812032, 812033, 81301, 81302, 813021, 813022, 813023 |  |
|  | Squamous-cell carcinoma | |  |  |  |  |  | 146 |
| Brain/central nervous system | | | 192.1, 193 |  | C47, C70-72, C75.2, C75.3 |  |  |  |
|  | Meningioma | |  |  |  |  |  | 461, 463, 466 |
|  | Low-grade glioma | |  |  |  |  |  | 475 |
|  | High-grade glioma | |  |  |  |  |  | 476 |
| Endocrine organs | | | 194, 195.0-1, 195.3, 195.5, 195.7-9 |  | C25.4, C73-74, C75.0-1, C75.4-5, C75.8-9 |  |  |  |
|  | Pancreatic islets | | 195.5 |  | C25.4 |  |  |  |
|  | Thyroid | | 194 |  | C73 |  |  |  |
|  |  | Papillary |  |  |  | 82603, 83403 |  |  |
|  |  | Follicular |  |  |  | 83303 |  |  |
|  | Adrenal glands | | 195.0 |  | C74 |  |  |  |
|  | Parathyroid gland | | 195.1 |  | C75.0 |  |  |  |
|  | Pituitary gland | | 195.3 |  | C75.1 |  |  |  |
| Thymus | | | 195.2 |  | C37 |  |  |  |
| Mediastinum | | | 164 |  | C38.1-3 |  |  |  |
| Pleura | | | 162.2 |  | C38.4 |  |  |  |
| Bone/articular cartilage | | | 196 |  | C40-41 |  |  |  |
| Retroperitoneum/peritoneum | | | 158, 197.4 |  | C48 |  |  |  |
| Connective tissue | | | 197.0-3, 197.7-9 |  | C49 |  |  |  |
| Eye | | | 192.0, 192.2-3. 192.7, 192.9 |  | C69 |  |  |  |
| Lymphoid neoplasms | | | 200-202, 204 |  | C81-85, C88, C91, C96 |  |  |  |
|  | Hodgkin lymphoma | | 201 |  | C81 |  |  |  |
|  |  | Nodular sclerosis | 201 |  | C81.1 | 96633, 96643, 96653, 96663, 96673 |  |  |
|  |  | Mixed cellularity | 201 |  | C81.2 | 96523 |  |  |
|  |  | Nodular lymphocyte | 201 |  | C81.0 | 96573, 96583, 96593 |  |  |
|  | Acute lymphocytic leukaemia | | 204.0 |  | C91.0 |  |  |  |
|  | Chronic lymphocytic leukaemia | | 204.1 |  | C91.1 |  |  |  |
|  | Diffuse large B-cell | | 200.1 |  | C83 | 96723, 96833, 96843, 968436, 96853, 968536, 96863 |  |  |
|  | Follicular | | 200.1 |  | C82 | 96143, 96903, 96913, 96923, 96933, 96963, 96973 |  |  |
|  | T-cell/natural killer-cell | | 200, 202 |  | C84-85 | 959035, 959135, 968435, 968535, 97003, 97013, 97023, 97043, 97053, 97073, 97133, 97143, 971435, 971436, 982535 |  |  |
| Multiple myeloma | | | 203 |  | C90 |  |  |  |
| Myeloid neoplasms | | | 205-207.3, 209 |  | C92-95, D46, D47.1 |  |  |  |
|  | Acute myeloid leukaemia | | 205.0 |  | C92.0 |  |  |  |
|  | Chronic myeloid leukaemia | | 205.1 |  | C92.1 |  |  |  |

Abbreviations: ICD, International Classification of Diseases.

* In the Swedish Cancer Register, all codes are converted into the ICD-7 edition for recording. However, the categorisation is more detailed for later ICD editions than for ICD-7. Therefore, we used the oldest ICD edition available through follow-up for cancer for which we judged the level of detail of the ICD edition to be sufficient.

# Aggressive prostate cancer was defined according to Hurwitz et al. (Recommended Definitions of Aggressive Prostate Cancer for Etiologic Epidemiologic Research. J Natl Cancer Inst. 2021 Jun 1;113(6):727-734), with the addition also of prostate-specific antigen level (PSA) ≥ 50 ng/mL in the aggressive prostate cancer category.

**Table S2. The combination of the International Classification of Diseases, 7th edition (ICD7) or ICD10 and/or Swedish PAD codes/C24.1 (histopathology) that were included even if the third digit of C24.1 was not 6**^*^

| Cancer | ICD7 | ICD10 | C24.1 |
| --- | --- | --- | --- |
| - | - | - | 441 (primarily endocrine organs, ICD7 195.0 and 195.7) |
| Uterus | 174 | - | 875 |
| Ovary and other/UNS female genital organs | 175 | - | 051, 053, 055, 063, 875 |
| Other/UNS female genital organs | 176.9 | - | 051, 053, 055, 063 |
| Pelvis | 180.1 | - | All codes |
| Ureter, bladder and other/UNS urinary tract | 181 | - | All codes |
| Non-melanoma | 191 | - | 715 |
| Eye | 192 | - | 461 |
| Brain/central nervous system | 192.1, 193 | - | All codes |
| Endocrine organs | 195 | - | All codes |
| Bone/articular cartilage | 196 | - | 865 |
| Connective tissue | 197 | - | 715 |
| Myelodysplastic syndromes | - | D46 | 223 |
| Essential thrombocythemia | - | D47.3 | 293 |

* In accordance with the Swedish Board of Health and Welfare: “Kodning i Cancerregistret 2022”, page 230 (<https://www.socialstyrelsen.se/globalassets/sharepoint-dokument/artikelkatalog/statistik/2023-5-8512.pdf>). This inclusion of cancers is used in Sweden’s official cancer statistics.

**Table S3. Number of individuals in each cohort**

| **Cohort (year of baseline examination)** | **Total** | **Men** | **Women** |
| --- | --- | --- | --- |
| Swedish Military Conscription Register (1969-2014) | 1 754 896 | 1 739 101 | 15 795 |
| Swedish Medical Birth Register (1982-2019) | 1 728 111 | 0 | 1 728 111 |
| Construction Workers Cohort (1971-93) | 262 622 | 244 863 | 17 759 |
| SIMPLER (1987-90, 1997, 2008 and 2019)^*^ | 90 629 | 33 802 | 56 827 |
| NSHDS (1985-2019)^†^ | 69 323 | 25 219 | 44 104 |
| Swedish Twin Registry (1963-2014)^‡^ | 67 142 | 27 228 | 39 914 |
| Malmö cohorts (1974-2019)^§^ | 45 696 | 24 182 | 21 514 |
| Women’s Lifestyle and Health (1991-92 and 2003-04) | 27 257 | 0 | 27 257 |
| Swedish National March Cohort (1997) | 24 866 | 8185 | 16 681 |
| West Sweden Asthma Study (2008-16) | 18 561 | 7655 | 10 906 |
| WICTORY (1989-2000) | 14 864 | 8686 | 6178 |
| Melanoma in South Sweden (1999-2004) | 12 743 | 0 | 12 743 |
| Lifegene (2009-18) | 11 219 | 3244 | 7975 |
| EpiHealth (2011-18) | 7844 | 4116 | 3728 |
| Obstructive Lung Disease in Norrbotten (1986-2016) | 6576 | 2868 | 3708 |

Abbreviations: SIMPLER, Swedish Infrastructure for Medical Population-based Life-course and Environmental Research; NSHDS, Northern Sweden Health and Disease Study; WICTORY, Westmannia Cardiovascular Risk Factors Study.

* The SIMPLER consists of the Swedish Mammography Cohort (SMC) and the Cohort of Swedish Men (COSM).

† The NSHDS consists of the Mammography Screening Project (MSP), the Northern Sweden Monica Project (MONICA), and the Västerbotten Intervention Programme (VIP).

‡ The Swedish Twin Registry consists of Q63, 67 and 70 (same-sex twins born in 1886-1925), Q73 (same-sex twins born in 1926-58), the Screening Across the Lifespan Twin Study (SALT, twins born in 1944-58), the TwinGene, the Swedish Twin Studies of Adults: Genes and Environment (STAGE), and the Young Adult Twins in Sweden Study (YATSS).

§ The Malmö cohorts consist of the Malmö Preventive Project (MPP), the Malmö Diet Cancer Study (MDCS), and the Malmö Offspring Study (MOS).

**Table S4. Hazard ratios (95% confidence intervals) of height per 5 cm, and per 5 kg/m^2^ higher body mass index in height unadjusted and adjusted analyses of all individuals.**

| **Cancer category** | **Per 5 cm higher height** | **Per 5 kg/m^2^ higher BMI, height not adjusted** | | **Per 5 kg/m^2^ higher BMI, height adjusted** | |
| --- | --- | --- | --- | --- | --- |
| Mediastinum | 1.23 (1.06-1.44) | 0.95 (0.69-1.30) | 0.96 (0.70-1.31) | |  |
| Thymus | 1.15 (1.04-1.28) | 1.07 (0.88-1.30) | 1.08 (0.89-1.32) | |  |
| Pituitary gland | 1.15 (1.12-1.19) | 1.19 (1.13-1.25) | 1.21 (1.15-1.27) | |  |
| Parathyroid gland | 1.13 (1.06-1.22) | 1.20 (1.06-1.37) | 1.22 (1.08-1.39) | |  |
| Thyroid | 1.13 (1.10-1.16) | 1.08 (1.04-1.13) | 1.10 (1.05-1.14) | |  |
| Malignant melanoma | 1.12 (1.11-1.13) | 1.05 (1.03-1.07) | 1.06 (1.04-1.08) | |  |
| Testis | 1.12 (1.10-1.14) | 0.95 (0.91-0.99) | 0.96 (0.92-1.00) | |  |
| Bone/articular cartilage | 1.12 (1.05-1.19) | 0.99 (0.88-1.11) | 1.00 (0.89-1.12) | |  |
| Vagina | 1.11 (0.94-1.31) | 1.22 (0.97-1.55) | 1.25 (0.98-1.59) | |  |
| Breast, postmenopausal | 1.11 (1.09-1.12) | 1.06 (1.04-1.08) | 1.08 (1.06-1.10) | |  |
| Connective tissue | 1.11 (1.07-1.15) | 1.20 (1.13-1.28) | 1.21 (1.14-1.29) | |  |
| Adrenal glands | 1.11 (1.03-1.19) | 1.18 (1.04-1.33) | 1.19 (1.05-1.35) | |  |
| Non-melanoma excluding basalioma | 1.10 (1.08-1.12) | 0.94 (0.91-0.97) | 0.95 (0.92-0.98) | |  |
| Lymphoid neoplasms | 1.10 (1.08-1.11) | 1.11 (1.09-1.14) | 1.12 (1.10-1.15) | |  |
| Myeloid neoplasms | 1.09 (1.07-1.12) | 1.15 (1.10-1.20) | 1.16 (1.11-1.20) | |  |
| Renal cell | 1.09 (1.07-1.11) | 1.45 (1.40-1.50) | 1.46 (1.42-1.51) | |  |
| Multiple myeloma | 1.09 (1.06-1.12) | 1.14 (1.08-1.19) | 1.15 (1.09-1.21) | |  |
| Retroperitoneum/peritoneum | 1.09 (1.00-1.18) | 1.05 (0.90-1.21) | 1.05 (0.91-1.22) | |  |
| Salivary glands | 1.08 (1.02-1.15) | 1.07 (0.96-1.20) | 1.08 (0.97-1.21) | |  |
| Breast, premenopausal | 1.08 (1.07-1.09) | 0.90 (0.89-0.92) | 0.91 (0.90-0.93) | |  |

**Table S5. Hazard ratios of site-specific cancers for current vs. never smokers in individuals with smoking information available^*^**

| **Hazard ratio range** | **Cancer forms and subgroups** |
| --- | --- |
| <1·00 | Head and neck-mucoepidermoid carcinoma; gastric-gastrointestinal stromal; small intestine-duodenum, ileum, adenocarcinoma, neuroendocrine; malignant melanoma; melanoma-superficial spreading, nodular, lentigo maligna; non-melanoma excluding basalioma; vulva; vagina; uterus; endometrium; endometrium-type I tumours; ovary-serous, endometrioid, clear cell; prostate; prostate-aggressive, non-aggressive; brain/central nervous system; low-grade glioma; high-grade glioma; endocrine organs; thyroid; thyroid-papillary, follicular; parathyroid gland; pituitary gland; thymus; bone/articular cartilage; retroperitoneum/peritoneum; eye; chronic lymphocytic leukaemia; diffuse large B-cell lymphoma; follicular lymphoma; multiple myeloma; chronic myeloid leukaemia |
| 1·00-1·24 | Lip; small intestine; small intestine-jejunum; colon; colon-proximal, distal, adenocarcinoma; rectum/anus; rectum; rectum/anus-adenocarcinoma; gallbladder; melanoma-acral lentiginous; breast-male; premenopausal breast; postmenopausal breast; cervix-adenocarcinoma; endometrium-type II tumours; ovary; testis; renal cell-papillary, chromophobe; meningioma; pancreatic islets; pleura; connective tissue; lymphoid neoplasms; Hodgkin lymphoma-nodular sclerosis, nodular lymphocyte; acute lymphocytic leukaemia |
| 1·25-1·49 | Salivary glands; gastric-non-cardia; small intestine-gastrointestinal stromal; colon-neuroendocrine; anus; rectum/anus-neuroendocrine; biliary tract; extrahepatic bile ducts, ampulla/papilla of Vater; penis; renal cell; renal cell-clear cell; adrenal glands; T-cell/natural killer-cell lymphoma; myeloid neoplasms; acute myeloid leukaemia |
| 1·50-1·99 | Oral cavity; tongue; oropharynx; nasal and paranasal sinuses; head and neck-adenocarcinoma; gastric; gastric-adenocarcinoma; liver/intrahepatic bile ducts; pancreas; cervix; cervix-squamous-cell carcinoma; ovary-mucinous; pelvis; mediastinum; Hodgkin lymphoma; Hodgkin lymphoma-mixed cellularity |
| ≥2·00 | Head and neck; mouth; pharynx; nasopharynx; hypopharynx; larynx; head and neck-adenoid cystic carcinoma, squamous-cell carcinoma; oesophagus; oesophagus-squamous-cell carcinoma, adenocarcinoma; gastric-cardia, neuroendocrine; lung; lung-adenocarcinoma, squamous-cell carcinoma, small cell, large cell; other urinary tract; ureter; bladder; other urinary tract-urothelial carcinoma, squamous-cell carcinoma |

***** Hazard ratios from Cox regression models with age as time scale, adjusted for baseline age (continuous), body mass index in four categories, mode of weight assessment, mode of height assessment, marital status, education, and birth country, and stratified by sex and calendar year of birth.

**Table S6. Characteristics of men in the study according to categories of body mass index and in total**

| **Characteristics** | | | **Underweight (BMI<18·5 kg/m^2^)** | **Normal weight**  **(BMI 18·5-24·9 kg/m^2^)** | **Overweight**  **(BMI 25-29·9 kg/m^2^)** | **Obesity**  **(BMI≥30 kg/m^2^)** | **Total** |
| --- | --- | --- | --- | --- | --- | --- | --- |
| **N (%)^*^** | | | 141 163 (7) | 1 588 998 (75) | 329 603 (15) | 69 385 (3) | 2 129 149 (100) |
| **Person-years from baseline date to end of follow-up** | | |  |  |  |  |  |
|  | Median (IQR) | | 34·3 (24·1-43·1) | 31·2 (21·8-39·9) | 25·7 (17·2-35·9) | 22·5 (15·3-31·3) | 30·2 (20·9-39·3) |
|  | Total person-years included (million) | | 4·6 | 48·3 | 8·7 | 1·6 | 63·2 |
| **Baseline age, years** | | |  |  |  |  |  |
|  | Mean (SD) | | 18·7 (3·5) | 21·3 (9·3) | 31·7 (17·1) | 31·2 (17·5) | 23·1 (11·8) |
|  | Category, n (%) | |  |  |  |  |  |
|  |  | <20 | 136 301 (97) | 1 362 040 (86) | 176 557 (54) | 40 335 (58) | 1 715 233 (81) |
|  |  | 20-29 | 3241 (2) | 76 298 (5) | 20 368 (6) | 2918 (4) | 102 825 (5) |
|  |  | 30-39 | 567 (0) | 48 909 (3) | 27 025 (8) | 3784 (5) | 80 285 (4) |
|  |  | ≥40 | 1054 (1) | 101 751 (6) | 105 653 (32) | 22 348 (32) | 230 806 (11) |
| **Weight measurement, n (%)** | | |  |  |  |  |  |
|  | Measured | | 140 392 (99) | 1 549 234 (97) | 298 287 (90) | 63 080 (91) | 2 050 993 (96) |
|  | Self-reported | | 771 (1) | 39 764 (3) | 31 316 (10) | 6305 (9) | 78 156 (4) |
| **Height measurement, n (%)** | | |  |  |  |  |  |
|  | Measured | | 140 392 (99) | 1 549 256 (97) | 298 298 (91) | 63 079 (91) | 2 051 025 (96) |
|  | Self-reported | | 771 (1) | 39 742 (3) | 31 305 (9) | 6306 (9) | 78 124 (4) |
| **Baseline smoking status, n (%)**^¤^ | | |  |  |  |  |  |
|  | Never | | 8705 (51) | 161 629 (49) | 54 934 (39) | 9392 (37) | 234 660 (46) |
|  | Former | | 2612 (15) | 53 559 (16) | 34 600 (25) | 7566 (29) | 98 337 (19) |
|  | Current | | 5771 (34) | 114 423 (35) | 50 546 (36) | 8783 (34) | 179 523 (35) |
| **Highest achieved education, n (%)^†^** ^¤^ | | |  |  |  |  |  |
|  | Pre-upper secondary school <9 years | | 2401 (2) | 83 678 (5) | 65 037 (20) | 12 747 (19) | 163 863 (8) |
|  | Pre-upper secondary school 9 years | | 18 502 (13) | 157 147 (10) | 29 874 (9) | 7541 (11) | 213 064 (10) |
|  | Upper secondary school <3 years | | 42 813 (30) | 428 121 (27) | 82 976 (26) | 17 554 (26) | 571 464 (27) |
|  | Upper secondary school 3 years | | 27 697 (20) | 331 845 (21) | 71 359 (22) | 17 754 (26) | 448 655 (21) |
|  | Post-upper secondary school <3 years | | 20 411 (14) | 233 495 (15) | 34 352 (11) | 6606 (10) | 294 864 (14) |
|  | Post-upper secondary school ≥3 years | | 28 313 (21) | 339 741 (22) | 39 175 (12) | 6000 (8) | 413 229 (20) |
| **Birth country for participant and parents, n (%)^‡^** ^¤^ | | |  |  |  |  |  |
|  | Born in Sweden, both parents born in Sweden | | 121 756 (86) | 1 363 151 (86) | 279 399 (85) | 57 425 (83) | 1 821 731 (85) |
|  | Born in Sweden, one parent born in Sweden | | 11 712 (8) | 118 487 (7) | 17 737 (5) | 4286 (6) | 152 222 (7) |
|  | Born in Sweden, both parents born abroad | | 3353 (3) | 41 526 (3) | 8352 (3) | 2149 (3) | 55 380 (3) |
|  | Born abroad | | 4339 (3) | 65 768 (4) | 24 097 (7) | 5520 (8) | 99 724 (5) |
| **Baseline marital status, n (%)^§^** ^¤^ | | |  |  |  |  |  |
|  | Unmarried | | 139 272 (99) | 1 437 051 (90) | 207 149 (63) | 47 109 (68) | 1 830 581 (86) |
|  | Married | | 1647 (1) | 135 300 (9) | 107 435 (32) | 18 749 (27) | 263 131 (12) |
|  | Divorced | | 187 (0) | 13 694 (1) | 12 097 (4) | 2887 (4) | 28 865 (2) |
|  | Widow/-er | | 41 (0) | 2617 (0) | 2796 (1) | 626 (1) | 6080 (0) |

Abbreviations: IQR, interquartile range.

* The percentages shown are row percentages, the rest in the table are column percentages.

¤ Number of individuals with missing values: highest achieved education, 24 010 (1%); birth country for participant and parents, 92 (< 1%); marital status, 492 (< 1%). For smoking status, there were 1 345 723 (63%) individuals with a missing value, 261 146 (12%) individuals were recorded as never or former smoker, 9760 (< 1%) individuals were current or former smoker.

† Highest achieved education through follow-up from the Population and Housing Census in 1970, and from the Longitudinal integration database for health insurance and labour market studies in 1990 onwards.

‡ From the Register of the Total Population.

§ From the Population and Housing Census in 1960 and 1965, and from the Register of the Total Population in 1968 onwards. Married, divorced and widow/-er also include registered partners, recorded as of 1998.

**Table S7. Characteristics of women in the study according to categories of body mass index and in total**

| **Characteristics** | | | **Underweight (BMI<18·5 kg/m^2^)** | **Normal weight**  **(BMI 18·5-24·9 kg/m^2^)** | **Overweight**  **(BMI 25-29·9 kg/m^2^)** | **Obesity**  **(BMI≥30 kg/m^2^)** | **Total** |
| --- | --- | --- | --- | --- | --- | --- | --- |
| **N (%)^*^** | | | 64 254 (3) | 1 323 816 (66) | 448 480 (22) | 176 650 (9) | 2 013 200 (100) |
| **Person-years from baseline date to end of follow-up** | | |  |  |  |  |  |
|  | Median (IQR) | | 19·3 (9·2-32·0) | 18·5 (9·6-28·3) | 16·1 (8·0-24·6) | 13·3 (6·3-20·1) | 17·5 (8·9-27·7) |
|  | Total person-years included (million) | | 1·3 | 25·7 | 7·5 | 2·5 | 37·0 |
| **Baseline age, years** | | |  |  |  |  |  |
|  | Mean (SD) | | 28·1 (7·8) | 30·7 (8·9) | 33·1 (11·3) | 32·9 (11·1) | 31·3 (9·7) |
|  | Category, n (%) | |  |  |  |  |  |
|  |  | <20 | 5224 (8) | 51 842 (4) | 13 252 (3) | 4464 (3) | 74 782 (4) |
|  |  | 20-29 | 39 686 (62) | 697 394 (53) | 207 768 (46) | 82 722 (47) | 1 027 570 (51) |
|  |  | 30-39 | 15 941 (25) | 444 152 (34) | 153 203 (34) | 61 201 (35) | 674 497 (34) |
|  |  | ≥40 | 3403 (5) | 130 428 (10) | 74 257 (17) | 28 263 (16) | 236 351 (12) |
| **Medical birth register, n (%)** | | |  |  |  |  |  |
|  | No | | 7 657 (12) | 173 955 (13) | 76 394 (17) | 27 083 (15) | 285 089 (14) |
|  | Yes | | 56 597 (88) | 1 149 861 (87) | 372 086 (83) | 149 567 (85) | 1 728 111 (86) |
| **Weight measurement, n (%)** | | |  |  |  |  |  |
|  | Measured | | 59 264 (92) | 1 215 919 (92) | 401 066 (89) | 161 230 (91) | 1 837 479 (91) |
|  | Self-reported | | 4990 (8) | 107 897 (8) | 47 414 (11) | 15 420 (9) | 175 721 (9) |
| **Height measurement, n (%)** | | |  |  |  |  |  |
|  | Measured | | 21 454 (33) | 382 551 (29) | 87 260 (19) | 22 889 (13) | 514 154 (26) |
|  | Self-reported | | 42 800 (67) | 941 265 (71) | 361 220 (81) | 153 761 (87) | 1 499 046 (74) |
| **Baseline smoking status, n (%)**^¤^ | | |  |  |  |  |  |
|  | Never | | 4311 (50) | 108 420 (55) | 41 137 (56) | 14 062 (56) | 167 930 (55) |
|  | Former | | 1472 (17) | 41 790 (22) | 16 268 (23) | 5668 (24) | 65 198 (22) |
|  | Current | | 2761 (33) | 43 350 (23) | 14 524 (21) | 4715 (20) | 65 350 (23) |
| **Highest achieved education, n (%)^†^** ^¤^ | | |  |  |  |  |  |
|  | Pre-upper secondary school <9 years | | 3044 (5) | 53 736 (4) | 34 250 (8) | 14 932 (9) | 105 962 (5) |
|  | Pre-upper secondary school 9 years | | 6603 (10) | 86 665 (7) | 32 720 (7) | 15 580 (9) | 141 568 (7) |
|  | Upper secondary school <3 years | | 15 301 (24) | 276 264 (21) | 99 426 (22) | 39 079 (22) | 430 070 (22) |
|  | Upper secondary school 3 years | | 12 453 (20) | 252 009 (19) | 95 560 (22) | 45 520 (26) | 405 542 (20) |
|  | Post-upper secondary school <3 years | | 8805 (14) | 202 766 (16) | 62 132 (14) | 22 173 (13) | 295 876 (15) |
|  | Post-upper secondary school ≥3 years | | 17 017 (27) | 438 799 (33) | 117 764 (27) | 36 540 (21) | 610 120 (30) |
| **Birth country for participant and parents, n (%)^‡^** ^¤^ | | |  |  |  |  |  |
|  | Born in Sweden, both parents born in Sweden | | 41 930 (65) | 971 430 (73) | 316 423 (70) | 122 404 (69) | 1 452 187 (72) |
|  | Born in Sweden, one parent born in Sweden | | 4141 (7) | 85 076 (6) | 27 027 (6) | 11 455 (7) | 127 699 (6) |
|  | Born in Sweden, both parents born abroad | | 1891 (3) | 35 231 (3) | 12 079 (3) | 5628 (3) | 54 829 (3) |
|  | Born abroad | | 16 276 (25) | 231 829 (18) | 92 854 (21) | 37 127 (21) | 378 086 (19) |
| **Baseline marital status, n (%)^§^** ^¤^ | | |  |  |  |  |  |
|  | Unmarried | | 32 274 (50) | 657 095 (50) | 210 720 (47) | 88 060 (50) | 988 149 (49) |
|  | Married | | 29 152 (46) | 597 307 (45) | 205 833 (46) | 75 655 (43) | 907 947 (45) |
|  | Divorced | | 2340 (4) | 57 344 (4) | 24 054 (5) | 9945 (6) | 93 683 (5) |
|  | Widow/-er | | 287 (0) | 9441 (1) | 6871 (2) | 2664 (1) | 19 263 (1) |

Abbreviations: IQR, interquartile range.

* The percentages shown are row percentages, the rest in the table are column percentages.

¤ Number of individuals with missing values: highest achieved education, 24 062 (1%); birth country for participant and parents, 399 (< 1%); marital status, 4158 (< 1%). For smoking status, there were 835 972 (42%) individuals with a missing value, 858 080 (43%) individuals were recorded as never or former smoker, 20 670 (1%) individuals were current or former smoker.

† Highest achieved education through follow-up from the Population and Housing Census in 1970, and from the Longitudinal integration database for health insurance and labour market studies (LISA) in 1990 onwards.

‡ From the Register of the Total Population.

§ From the Population and Housing Census in 1960 and 1965, and from the Register of the Total Population in 1968 onwards. Married, divorced and widow/-er also include registered partners, recorded as of 1998.

**Table S8. Hazard ratios (95% confidence intervals) of site-specific cancers according to body mass index level in men and women combined^*^**

| **Cancer category** | | | **No· at risk/cases^†^** | **HR (95% CI), P-value** | | | | **HR (95% CI), P-value, for Per 5 kg/m^2^ higher BMI^*^** |
| --- | --- | --- | --- | --- | --- | --- | --- | --- |
|  |  |  |  | **Underweight** | **Normal weight** | **Overweight** | **Obesity** |  |
|  |  |  |  | **(BMI<18·5 kg/m^2^)^*^** | **(BMI 18·5-24·9 kg/m^2^)^*^** | **(BMI 25-29·9 kg/m^2^)^*^** | **(BMI≥30 kg/m^2^)^*^** |  |
| Head and neck | | | 4 142 349/7853 | 0·97 (0·88-1·08), 0·56 | reference | 0·95 (0·90-1·01), 0·13 | 0·94 (0·84-1·05), 0·35 | 0·97 (0·94-1·01), 0·18 |
|  | Oral cavity | | 4 142 349/2688 | 0·98 (0·80-1·19), 0·81 | reference | 1·08 (0·98-1·18), 0·12 | 1·11 (0·94-1·32), 0·20 | 1·06 (1·00-1·12), 0·048 |
|  |  | Lip | 4 142 349/760 | 0·81 (0·48-1·36), 0·42 | reference | 1·18 (1·01-1·39), 0·042 | 1·17 (0·87-1·57), 0·29 | 1·13 (1·01-1·25)^¤^, 0·033 |
|  |  | Tongue | 4 142 349/925 | 0·84 (0·61-1·15), 0·27 | reference | 0·99 (0·83-1·18), 0·90 | 1·45 (1·09-1·91)^¤^, 0·0080 | 1·14 (1·04-1·26), 0·0063 |
|  |  | Mouth | 4 142 349/1005 | 1·19 (0·89-1·58), 0·24 | reference | 1·06 (0·91-1·24), 0·44 | 0·85 (0·62-1·16), 0·33 | 0·95 (0·86-1·05), 0·35 |
|  | Salivary glands | | 4 142 349/675 | 0·63 (0·40-0·98), 0·04 | reference | 1·04 (0·86-1·27), 0·64 | 0·84 (0·58-1·23), 0·39 | 1·07 (0·96-1·20), 0·22 |
|  | Pharynx | | 4 142 349/2982 | 0·99 (0·85-1·15), 0·84 | reference | 0·85 (0·76-0·94), 0·0021 | 0·71 (0·57-0·90), 0·0041 | 0·88 (0·83-0·94), 0·00010 |
|  |  | Oropharynx | 4 142 349/2386 | 0·93 (0·79-1·10), 0·39 | reference | 0·91 (0·80-1·02), 0·13 | 0·90 (0·71-1·15), 0·44 | 0·95 (0·89-1·02), 0·20 |
|  |  | Nasopharynx | 4 142 349/300 | 0·85 (0·49-1·47), 0·57 | reference | 0·83 (0·61-1·13), 0·24 | 0·54 (0·27-1·05), 0·069 | 0·86 (0·72-1·03), 0·10 |
|  |  | Hypopharynx | 4 142 349/300 | 1·94 (1·24-3·03), 0·0039 | reference | 0·57 (0·43-0·77), 0·00025 | 0·10 (0·02-0·39), 0·0010 | 0·48 (0·39-0·59), <0·0001 |
|  | Nasal and paranasal sinuses | | 4 142 349/431 | 0·50 (0·26-0·94), 0·030 | reference | 1·09 (0·86-1·39), 0·44 | 1·55 (1·06-2·26), 0·021 | 1·16 (1·01-1·33), 0·030 |
|  | Larynx | | 4 142 349/1052 | 1·31 (0·98-1·76), 0·072 | reference | 0·83 (0·72-0·97), 0·016 | 0·90 (0·68-1·19)^¤^, 0·47 | 0·87 (0·79-0·97)^¤^, 0·0096 |
|  | Adenoid cystic carcinoma | | 4 142 349/245 | NA^§^ | NA^§^ | NA^§^ | NA^§^ | 0·94 (0·77-1·14), 0·57 |
|  | Mucoepidermoid carcinoma | | 4 142 349/204 | NA^§^ | NA^§^ | NA^§^ | NA^§^ | 0·96 (0·78-1·19), 0·71 |
|  | Adenocarcinoma | | 4 042 179/208 | NA^§^ | NA^§^ | NA^§^ | NA^§^ | 1·26 (1·04-1·52)^¤^, 0·022 |
|  | Squamous-cell carcinoma | | 4 142 349/6410 | 1·01 (0·90-1·13), 0·90 | reference | 0·96 (0·90-1·02), 0·21 | 0·96 (0·85-1·08)^¤^, 0·56 | 0·96 (0·93-1·00)^¤^, 0·092 |
| Oesophagus | | | 4 142 349/2236 | 1·25 (1·03-1·52), 0·029 | reference | 1·03 (0·93-1·14), 0·55 | 1·34 (1·12-1·59)^¤^, 0·0010 | 1·05 (0·98-1·12)^¤^, 0·13 |
|  | Squamous-cell carcinoma | | 4 142 349/927 | 1·82 (1·36-2·44), <0·0001 | reference | 0·55 (0·47-0·65), <0·0001 | 0·52 (0·37-0·73)^¤^, 0·00013 | 0·54 (0·48-0·61), <0·0001 |
|  | [Adenocarcinoma^‡^](https://en.wikipedia.org/wiki/Pilcrow) | | 4 142 349/1193 | 0·92 (0·69-1·24), 0·58 | reference | 1·72 (1·50-1·98), <0·0001 | 2·73 (2·20-3·40), <0·0001 | 1·61 (1·49-1·74), <0·0001 |
| Gastric | | | 4 142 349/5399 | 1·07 (0·91-1·25), 0·43 | reference | 0·98 (0·92-1·04), 0·48 | 0·99 (0·89-1·11), 0·92 | 1·01 (0·97-1·05), 0·72 |
|  | [Cardia^‡^](https://en.wikipedia.org/wiki/Pilcrow) | | 4 142 349/1465 | 0·82 (0·62-1·09), 0·17 | reference | 1·31 (1·15-1·48), <0·0001 | 1·64 (1·32-2·03), <0·0001 | 1·31 (1·21-1·41), <0·0001 |
|  | Non-cardia | | 4 142 349/3892 | 1·23 (1·01-1·48), 0·036 | reference | 0·90 (0·83-0·96), 0·0023 | 0·85 (0·75-0·97), 0·017 | 0·92 (0·88-0·97), 0·0069 |
|  | Adenocarcinoma | | 4 142 349/4743 | 1·08 (0·91-1·28), 0·36 | reference | 0·96 (0·89-1·02), 0·19 | 0·93 (0·83-1·05), 0·27 | 0·98 (0·94-1·03), 0·49 |
|  | Neuroendocrine | | 4 142 349/170 | NA^§^ | NA^§^ | NA^§^ | NA^§^ | 1·19 (0·97-1·46), 0·091 |
|  | Gastrointestinal stromal | | 4 142 349/227 | NA^§^ | NA^§^ | NA^§^ | NA^§^ | 1·29 (1·08-1·55), 0·0050 |
| Small intestine | | | 4 142 349/1431 | 0·84 (0·63-1·11), 0·20 | reference | 1·35 (1·19-1·54), <0·0001 | 1·55 (1·25-1·93), <0·0001 | 1·26 (1·17-1·35), <0·0001 |
|  | Duodenum | | 4 142 349/291 | 0·66 (0·32-1·34), 0·25 | reference | 1·49 (1·14-1·96), 0·0034 | 1·04 (0·60-1·83), 0·83 | 1·16 (0·98-1·38), 0·068 |
|  | Jejunum | | 4 092 313/125 | NA^§^ | NA^§^ | NA^§^ | NA^§^ | 1·28 (1·00-1·63), 0·051 |
|  | Ileum | | 4 092 313/348 | 1·17 (0·72-1·90), 0·53 | reference | 1·39 (1·06-1·82), 0·016 | 2·35 (1·59-3·47), <0·0001 | 1·39 (1·21-1·60), <0·0001 |
|  | Adenocarcinoma | | 4 142 349/418 | 0·94 (0·56-1·56), 0·81 | reference | 1·27 (1·00-1·59), 0·044 | 0·84 (0·51-1·37), 0·50 | 1·04 (0·90-1·20), 0·59 |
|  | Neuroendocrine | | 4 142 349/778 | 0·71 (0·48-1·07), 0·10 | reference | 1·37 (1·14-1·63), 0·00045 | 2·04 (1·56-2·68), <0·0001 | 1·39 (1·26-1·52), <0·0001 |
|  | Gastrointestinal stromal | | 4 142 349/127 | NA^§^ | NA^§^ | NA^§^ | NA^§^ | 1·18 (0·91-1·53), 0·22 |
| [Colon^‡^](https://en.wikipedia.org/wiki/Pilcrow) | | | 4 142 349/17 866 | 0·99 (0·91-1·08), 0·75 | reference | 1·16 (1·12-1·20), <0·0001 | 1·37 (1·29-1·45)^¤^, <0·0001 | 1·15 (1·13-1·18)^¤^, <0·0001 |
|  | *Proximal* | | 4 142 349/9542 | 1·08 (0·96-1·02), 0·21 | reference | 1·17 (1·12-1·23), <0·0001 | 1·33 (1·23-1·45)^¤^, <0·0001 | 1·15 (1·11-1·18)^¤^, <0·0001 |
|  | *Distal* | | 4 142 349/7253 | 0·91 (0·80-1·03), 0·14 | reference | 1·15 (1·09-1·22), <0·0001 | 1·43 (1·30-1·57)^¤^, <0·0001 | 1·16 (1·13-1·20)^¤^, <0·0001 |
|  | *Adenocarcinoma* | | 4 142 349/16 801 | 0·97 (0·89-1·06), 0·45 | reference | 1·16 (1·12-1·20), <0·0001 | 1·38 (1·30-1·46)^¤^, <0·0001 | 1·16 (1·13-1·18)^¤^, <0·0001 |
|  | *Neuroendocrine* | | 4 142 349/774 | 1·23 (0·92-1·65), 0·16 | reference | 1·10 (0·90-1·34), 0·34 | 1·53 (1·14-2·06), 0·0051 | 1·15 (1·05-1·27), 0·0044 |
| Rectum/anus | | | 4 142 349/11 644 | 0·99 (0·90-1·10), 0·85 | reference | 1·07 (1·02-1·12), 0·0036 | 1·19 (1·10-1·29)^¤^, <0·0001 | 1·08 (1·06-1·12)^¤^, <0·0001 |
|  | [Rectum^‡^](https://en.wikipedia.org/wiki/Pilcrow) | | 4 142 349/10 846 | 1·00 (0·90-1·10), 0·90 | reference | 1·08 (1·03-1·13), 0·0012 | 1·20 (1·11-1·30), <0·0001 | 1·09 (1·06-1·12)^¤^, <0·0001 |
|  | Anus | | 4 142 349/801 | 0·95 (0·67-1·34), 0·75 | reference | 0·91 (0·75-1·10), 0·35 | 1·06 (0·77-1·46), 0·66 | 1·01 (0·91-1·12)^¤^, 0·78 |
|  | Adenocarcinoma | | 4 142 349/10 519 | 1·00 (0·91-1·11), 0·94 | reference | 1·08 (1·04-1·14), 0·00068 | 1·20 (1·10-1·30), <0·0001 | 1·09 (1·06-1·13), <0·0001 |
|  | Neuroendocrine | | 4 142 349/230 | NA^§^ | NA^§^ | NA^§^ | NA^§^ | 1·09 (0·90-1·32), 0·39 |
| [Liver/intrahepatic bile ducts^‡^](https://en.wikipedia.org/wiki/Pilcrow) | | | 4 142 349/3284 | 0·94 (0·79-1·13), 0·51 | reference | 1·34 (1·23-1·46), <0·0001 | 2·35 (2·08-2·67)^¤^, <0·0001 | 1·40 (1·33-1·47)^¤^, <0·0001 |
| Biliary tract | | | 4 142 349/2002 | 0·79 (0·59-1·06), 0·12 | reference | 1·24 (1·12-1·38), <0·0001 | 1·48 (1·26-1·74), <0·0001 | 1·24 (1·16-1·31), <0·0001 |
|  | [Gallbladder^‡^](https://en.wikipedia.org/wiki/Pilcrow) | | 4 142 349/917 | 0·78 (0·48-1·25), 0·29 | reference | 1·30 (1·12-1·51), 0·00038 | 1·52 (1·21-1·91), 0·00019 | 1·25 (1·15-1·36), <0·0001 |
|  | Extrahepatic bile ducts | | 4 142 349/589 | 0·66 (0·39-1·10), 0·11 | reference | 1·28 (1·05-1·55), 0·014 | 1·49 (1·07-2·08), 0·016 | 1·25 (1·11-1·41), <0·0001 |
|  | Ampulla/papilla of Vater | | 4 142 349/236 | NA^§^ | NA^§^ | NA^§^ | NA^§^ | 1·04 (0·86-1·27), 0·72 |
| [Pancreas^‡^](https://en.wikipedia.org/wiki/Pilcrow) | | | 4 142 349/5509 | 1·02 (0·89-1·18), 0·78 | reference | 1·03 (0·97-1·10), 0·29 | 1·26 (1·13-1·40), <0·0001 | 1·11 (1·07-1·16), <0·0001 |
| Lung/bronchus | | | 4 142 349/16 856 | 1·55 (1·44-1·67), <0·0001 | reference | 0·79 (0·76-0·81), <0·0001 | 0·72 (0·67-0·78)^¤^, <0·0001 | 0·78 (0·76-0·79)^¤^, <0·0001 |
|  | Adenocarcinoma | | 4 142 349/6609 | 1·61 (1·45-1·78), <0·0001 | reference | 0·76 (0·72-0·81), <0·0001 | 0·74 (0·65-0·83)^¤^, <0·0001 | 0·75 (0·72-0·78)^¤^, <0·0001 |
|  | Squamous-cell carcinoma | | 4 142 349/2692 | 1·41 (1·14-1·74), 0·0013 | reference | 0·91 (0·84-1·00), 0·044 | 0·84 (0·70-0·99), 0·045 | 0·90 (0·84-0·96), 0·00067 |
|  | Small cell | | 4 142 349/2002 | 1·32 (1·06-1·65), 0·015 | reference | 0·95 (0·85-1·05), 0·32 | 0·94 (0·77-1·14), 0·58 | 0·94 (0·88-1·01), 0·11 |
|  | Large cell | | 4 042 179/479 | 1·48 (0·96-2·28), 0·077 | reference | 0·69 (0·55-0·87), 0·0020 | 0·66 (0·43-1·01)^¤^, 0·057 | 0·71 (0·61-0·83), <0·0001 |
| Malignant melanoma | | | 4 142 349/22 167 | 0·79 (0·74-0·85), <0·0001 | reference | 1·05 (1·01-1·08), 0·075 | 0·96 (0·89-1·02)^¤^, 0·29 | 1·05 (1·03-1·07)^¤^, <0·0001 |
|  | Acral lentiginous | | 4 042 179/310 | 0·58 (0·30-1·14), 0·12 | reference | 0·81 (0·59-1·11), 0·17 | 1·04 (0·64-1·70), 0·84 | 1·08 (0·93-1·27), 0·34 |
|  | Superficial spreading | | 4 042 179/12 855 | 0·74 (0·67-0·81), <0·0001 | reference | 1·03 (0·98-1·08), 0·28 | 0·91 (0·83-1·00)^¤^, 0·039 | 1·04 (1·02-1·07)^¤^, 0·0011 |
|  | Nodular | | 4 042 179/2281 | 0·79 (0·63-0·98), 0·034 | reference | 1·14 (1·02-1·27), 0·017 | 1·09 (0·89-1·33), 0·40 | 1·10 (1·04-1·17), 0·0018 |
|  | Lentigo maligna | | 4 042 179/621 | 1·06 (0·70-1·60), 0·78 | reference | 1·06 (0·87-1·29), 0·57 | 0·79 (0·53-1·18), 0·25 | 0·96 (0·84-1·08), 0·49 |
| Non-melanoma excluding basalioma | | | 4 142 349/11 437 | 0·95 (0·85-1·07), 0·40 | reference | 0·95 (0·90-0·99), 0·013 | 0·89 (0·81-0·97)^¤^, 0·011 | 0·94 (0·91-0·97)^¤^, <0·0001 |
| [Renal cell^‡^](https://en.wikipedia.org/wiki/Pilcrow) | | | 4 142 349/6193 | 0·81 (0·71-0·93), 0·0030 | reference | 1·47 (1·39-1·57), <0·0001 | 2·16 (1·96-2·37), <0·0001 | 1·45 (1·40-1·50), <0·0001 |
|  | *Clear cell* | | 3 837 868/2962 | 0·77 (0·64-0·92), 0·0050 | reference | 1·69 (1·55-1·86), <0·0001 | 2·39 (2·07-2·76), <0·0001 | 1·53 (1·46-1·61), <0·0001 |
|  | *Papillary* | | 4 042 179/466 | 1·02 (0·68-1·53), 0·91 | reference | 1·38 (1·09-1·74), 0·082 | 1·56 (1·02-2·40), 0·045 | 1·25 (1·09-1·43), 0·0020 |
|  | *Chromophobe* | | 3 837 868/282 | 1·01 (0·60-1·72), 0·95 | reference | 1·18 (0·86-1·63), 0·31 | 2·06 (1·31-3·24), 0·0019 | 1·31 (1·12-1·54), 0·00076 |
| Other urinary tract | | | 4 142 349/13 135 | 1·09 (0·99-1·20), 0·10 | reference | 0·95 (0·91-0·99), 0·020 | 1·02 (0·95-1·10), 0·47 | 0·98 (0·95-1·01), 0·11 |
|  | Pelvis | | 4 142 349/629 | 1·35 (0·90-2·01), 0·14 | reference | 0·95 (0·78-1·14), 0·57 | 0·75 (0·51-1·11), 0·17 | 0·93 (0·82-1·06), 0·28 |
|  | Ureter | | 4 142 349/250 | 1·35 (0·70-2·61), 0·37 | reference | 1·02 (0·76-1·37), 0·95 | 1·28 (0·79-2·08), 0·34 | 0·98 (0·76-1·25), 0·76 |
|  | Bladder | | 4 142 349/12 105 | 1·06 (0·96-1·17), 0·28 | reference | 0·95 (0·91-0·99), 0·023 | 1·03 (0·95-1·11), 0·37 | 0·98 (0·95-1·01), 0·38 |
|  | Urothelial carcinoma | | 3 837 868/6122 | 1·03 (0·91-1·17), 0·63 | reference | 1·01 (0·95-1·07), 0·86 | 1·08 (0·96-1·21), 0·22 | 1·02 (0·98-1·06), 0·38 |
|  | Squamous-cell carcinoma | | 4 142 349/118 | NA^§^ | NA^§^ | NA^§^ | NA^§^ | 1·02 (0·77-1·34), 0·88 |
| Brain/central nervous system** | | | 4 142 349/12 609 | 0·93 (0·86-1·02), 0·10 | reference | 1·04 (0·99-1·09), 0·091 | 1·01 (0·93-1·10), 0·75 | 1·03 (1·01-1·06), 0·0094 |
|  | [Meningioma^‡^](https://en.wikipedia.org/wiki/Pilcrow) | | 4 142 349/3699 | 0·97 (0·82-1·15), 0·72 | reference | 1·13 (1·04-1·22), 0·0040 | 1·21 (1·06-1·39)^¤^, 0·0041 | 1·11 (1·06-1·16), <0·0001 |
|  | Low-grade glioma | | 4 142 349/1583 | 1·03 (0·83-1·26), 0·82 | reference | 1·07 (0·94-1·23), 0·30 | 0·72 (0·53-0·97), 0·03 | 0·99 (0·92-1·07), 0·79 |
|  | High-grade glioma | | 4 142 349/3990 | 0·88 (0·76-1·03), 0·10 | reference | 1·02 (0·94-1·11), 0·54 | 0·97 (0·83-1·14), 0·76 | 1·03 (0·98-1·08), 0·18 |
| Endocrine organs | | | 4 142 349/11 224 | 0·88 (0·80-0·97), 0·0066 | reference | 1·17 (1·12-1·23), <0·0001 | 1·34 (1·24-1·45), <0·0001 | 1·14 (1·11-1·17)^¤^, <0·0001 |
|  | Pancreatic islets | | 4 142 349/480 | 1·03 (0·69-1·55), 0·89 | reference | 1·37 (1·09-1·73), 0·0070 | 1·36 (0·90-2·05), 0·13 | 1·20 (1·06-1·37), 0·0032 |
|  | [Thyroid^‡^](https://en.wikipedia.org/wiki/Pilcrow) | | 4 142 349/4106 | 0·92 (0·79-1·08), 0·33 | reference | 1·11 (1·02-1·20), 0·0084 | 1·18 (1·05-1·34)^¤^, 0·0055 | 1·08 (1·04-1·13)^¤^, <0·0001 |
|  |  | *Papillary* | 4 042 179/2809 | 0·93 (0·76-1·13), 0·46 | reference | 1·10 (1·00-1·21), 0·053 | 1·24 (1·07-1·43)^¤^, 0·0035 | 1·08 (1·03-1·14)^¤^, 0·013 |
|  |  | *Follicular* | 4 042 179/408 | 0·98 (0·58-1·65), 0·93 | reference | 1·31 (1·03-1·66), 0·026 | 1·20 (0·81-1·78), 0·36 | 1·17 (1·03-1·32), 0·015 |
|  | Adrenal glands | | 4 142 349/479 | 0·73 (0·44-1·19), 0·20 | reference | 1·17 (0·93-1·48), 0·19 | 1·50 (1·03-2·17), 0·033 | 1·18 (1·04-1·33), 0·011 |
|  | Parathyroid gland | | 4 142 349/3144 | 0·81 (0·66-0·99), 0·034 | reference | 1·13 (1·03-1·24), 0·0036 | 1·41 (1·23-1·62), <0·0001 | 1·16 (1·11-1·22), <0·0001 |
|  | Pituitary gland | | 4 142 349/2936 | 0·92 (0·77-1·09), 0·32 | reference | 1·30 (1·18-1·43), <0·0001 | 1·57 (1·35-1·83), <0·0001 | 1·19 (1·13-1·25), <0·0001 |
| Thymus | | | 4 142 349/225 | NA^§^ | NA^§^ | NA^§^ | NA^§^ | 1·07 (0·88-1·30), 0·48 |
| Mediastinum | | | 4 142 349/100 | NA^§^ | NA^§^ | NA^§^ | NA^§^ | 0·95 (0·69-1·30), 0·74 |
| Pleura | | | 4 142 349/864 | 1·11 (0·71-1·73), 0·67 | reference | 0·81 (0·69-0·94), 0·0070 | 0·66 (0·47-0·93), 0·021 | 0·82 (0·73-0·92), 0·00072 |
| Bone/articular cartilage | | | 4 142 349/687 | 1·15 (0·86-1·55), 0·35 | reference | 1·10 (0·89-1·36), 0·36 | 1·13 (0·77-1·65), 0·52 | 0·99 (0·88-1·11), 0·89 |
| Retroperitoneum/peritoneum | | | 4 142 349/392 | 0·90 (0·53-1·55), 0·73 | reference | 1·05 (0·82-1·35), 0·71 | 0·97 (0·61-1·53), 0·87 | 1·05 (0·90-1·21), 0·57 |
| Connective tissue | | | 4 142 349/2029 | 0·80 (0·63-1·01), 0·057 | reference | 1·26 (1·13-1·40), <0·0001 | 1·34 (1·11-1·62), 0·0020 | 1·20 (1·13-1·28)^¤^, <0·0001 |
| Eye | | | 4 142 349/910 | 0·89 (0·64-1·24), 0·49 | reference | 1·00 (0·84-1·18), 0·98 | 1·28 (0·96-1·71), 0·070 | 1·07 (0·97-1·19), 0·13 |
| Lymphoid neoplasms | | | 4 142 349/16 018 | 0·88 (0·81-0·96), 0·20 | reference | 1·09 (1·05-1·14), <0·0001 | 1·22 (1·14-1·31), <0·0001 | 1·11 (1·09-1·14)^¤^, <0·0001 |
|  | Hodgkin lymphoma | | 4 142 349/2239 | 1·05 (0·88-1·25), 0·61 | reference | 1·23 (1·10-1·38), 0·00031 | 1·38 (1·13-1·67), 0·0011 | 1·18 (1·11-1·25), <0·0001 |
|  |  | Nodular sclerosis | 4 042 179/880 | 1·15 (0·87-1·52), 0·32 | reference | 1·10 (0·92-1·32), 0·30 | 1·04 (0·76-1·43), 0·80 | 1·07 (0·98-1·18), 0·14 |
|  |  | Mixed cellularity | 4 042 179/237 | NA^§^ | NA^§^ | NA^§^ | NA^§^ | 1·30 (1·10-1·54), 0·0025 |
|  |  | Nodular lymphocyte | 4 042 179/166 | NA^§^ | NA^§^ | NA^§^ | NA^§^ | 1·72 (1·45-2·03), <0·0001 |
|  | Acute lymphocytic leukaemia | | 4 142 349/539 | 0·67 (0·43-1·03), 0·065 | reference | 1·01 (0·79-1·28), 0·95 | 1·61 (1·13-2·29), 0·0076 | 1·18 (1·05-1·33), 0·0049 |
|  | Chronic lymphocytic leukaemia | | 4 142 349/2854 | 0·85 (0·69-1·05), 0·14 | reference | 1·03 (0·94-1·13), 0·51 | 1·05 (0·89-1·24), 0·53 | 1·06 (1·00-1·12), 0·045 |
|  | Diffuse large B-cell | | 4 042 179/2775 | 0·91 (0·75-1·10), 0·31 | reference | 1·26 (1·14-1·38), <0·0001 | 1·53 (1·31-1·79), <0·0001 | 1·23 (1·17-1·30), <0·0001 |
|  | Follicular | | 4 042 179/1931 | 0·91 (0·73-1·14), 0·42 | reference | 1·04 (0·93-1·17), 0·49 | 1·15 (0·94-1·41), 0·17 | 1·09 (1·02-1·16), 0·015 |
|  | T-cell/natural killer-cell | | 4 042 179/978 | 0·93 (0·68-1·27), 0·65 | reference | 0·89 (0·75-1·05), 0·15 | 1·01 (0·75-1·35), 0·99 | 0·99 (0·90-1·10)^¤^, 0·83 |
| [Multiple myeloma^‡^](https://en.wikipedia.org/wiki/Pilcrow) | | | 4 142 349/3720 | 0·74 (0·61-0·91), 0·0030 | reference | 1·17 (1·08-1·27), <0·0001 | 1·12 (0·97-1·29), 0·10 | 1·14 (1·08-1·19), <0·0001 |
| Myeloid neoplasms | | | 4 142 349/4784 | 0·87 (0·75-1·01), 0·063 | reference | 1·05 (0·98-1·13), 0·18 | 1·41 (1·26-1·59), <0·0001 | 1·15 (1·10-1·20), <0·0001 |
|  | Acute myeloid leukaemia | | 4 142 349/1498 | 0·81 (0·61-1·07), 0·13 | reference | 1·01 (0·88-1·15), 0·87 | 1·24 (1·00-1·54), 0·037 | 1·13 (1·05-1·22), 0·00071 |
|  | Chronic myeloid leukaemia | | 4 142 349/1002 | 0·72 (0·52-0·99), 0·040 | reference | 0·99 (0·83-1·18), 0·92 | 1·47 (1·13-1·92), 0·0042 | 1·17 (1·07-1·28), 0·00037 |

Abbreviations: BMI, body mass index.

* Hazard ratios from Cox regression models with age as time scale, adjusted for baseline age (continuous), weight assessment from the Medical Birth Register (yes/no), mode of weight assessment, mode of height assessment, marital status, education level, and birth country, and stratified by sex and calendar year of birth.

† For cancers that could only be identified by later international classification of disease (ICD) codes than ICD-7, follow-up started at the year of the start of the respective ICD edition or at baseline, whichever came later.

‡ Established obesity-related cancer.

§ The number of cancer cases was considered too low for analysis (<250 cases for categorical body mass index and <100 cases for per 5 kg/m^2^).

¤ P _sex-interaction_<0**·**05, calculated by adding product terms of sex and BMI categories or per 5 kg/m^2^ higher body mass index level in the Cox model using Wald test.

** P=0**·**018 for heterogeneity in hazard ratio per 5 kg/m^2^ of BMI between meningioma, low-grade glioma, and high-grade glioma.

**Table S9. Hazard ratios (95% confidence intervals) of site-specific cancers according to body mass index level in men^*^**

| **Cancer category** | | | **No. at risk/cases^†^** | **HR (95% CI), P-value** | | | | **HR (95% CI), P-value, for Per 5 kg/m^2^ higher BMI^*^** |
| --- | --- | --- | --- | --- | --- | --- | --- | --- |
|  |  |  |  | **Underweight** | **Normal weight** | **Overweight** | **Obesity** |  |
|  |  |  |  | **(BMI<18·5 kg/m^2^)^*^** | **(BMI 18·5-24·9 kg/m^2^)^*^** | **(BMI 25-29·9 kg/m^2^)^*^** | **(BMI≥30 kg/m^2^)^*^** |  |
| Head and neck | | | 2 129 149/5943 | 1·00 (0·89-1·12), 0·97 | reference | 0·92 (0·86-0·99), 0·021 | 0·91 (0·79-1·05), 0·22 | 0·95 (0·91-0·99), 0·027 |
|  | Oral cavity | | 2 129 149/1890 | 1·00 (0·80-1·24), 0·96 | reference | 1·03 (0·91-1·15), 0·65 | 1·06 (0·85-1·33), 0·54 | 1·03 (0·96-1·11), 0·38 |
|  |  | Lip | 2 129 149/597 | 0·92 (0·52-1·62), 0·78 | reference | 1·20 (1·00-1·44), 0·045 | 1·22 (0·88-1·71), 0·22 | 1·17 (1·03-1·32), 0·017 |
|  |  | Tongue | 2 129 149/627 | 0·68 (0·47-1·00), 0·051 | reference | 0·86 (0·69-1·08), 0·21 | 1·29 (0·86-1·92), 0·20 | 1·09 (0·96-1·25), 0·17 |
|  |  | Mouth | 2 129 149/667 | 1·38 (1·01-1·87), 0·041 | reference | 0·98 (0·80-1·19), 0·84 | 0·75 (0·48-1·17), 0·22 | 0·87 (0·76-0·99), 0·048 |
|  | Salivary glands | | 2 129 149/399 | 0·78 (0·48-1·26), 0·31 | reference | 1·20 (0·93-1·55), 0·16 | 1·04 (0·60-1·80), 0·87 | 1·16 (0·99-1·37), 0·059 |
|  | Pharynx | | 2 129 149/2396 | 1·02 (0·87-1·19), 0·88 | reference | 0·82 (0·73-0·93), 0·0018 | 0·70 (0·53-0·93), 0·016 | 0·88 (0·82-0·95), 0·00082 |
|  |  | Oropharynx | 2 129 149/1920 | 0·96 (0·80-1·14), 0·57 | reference | 0·89 (0·78-1·03), 0·12 | 0·93 (0·69-1·25), 0·68 | 0·96 (0·88-1·04), 0·36 |
|  |  | Nasopharynx | 2 129 149/215 | NA**^§^** | NA**^§^** | NA**^§^** | NA**^§^** | 0·91 (0·72-1·15), 0·43 |
|  |  | Hypopharynx | 2 129 149/265 | 1·93 (1·20-3·09), 0·0063 | reference | 0·54 (0·40-0·75), 0·0002 | 0·06 (0·01-0·40), 0·0042 | 0·45 (0·36-0·57), <0·0001 |
|  | Nasal and paranasal sinuses | | 2 129 149/293 | 0·45 (0·21-0·96), 0·038 | reference | 1·27 (0·95-1·69), 0·11 | 1·75 (1·07-2·86), 0·024 | 1·19 (1·00-1·43), 0·051 |
|  | Larynx | | 2 129 149/944 | 1·25 (0·91-1·70), 0·17 | reference | 0·77 (0·66-0·91), 0·0015 | 0·79 (0·58-1·08), 0·16 | 0·82 (0·73-0·91), 0·00049 |
|  | Adenoid cystic carcinoma | | 2 129 149/128 | NA**^§^** | NA**^§^** | NA**^§^** | NA**^§^** | 1·17 (0·88-1·55), 0·27 |
|  | Mucoepidermoid carcinoma | | 2 129 149/109 | NA**^§^** | NA**^§^** | NA**^§^** | NA**^§^** | 1·21 (0·90-1·63), 0·21 |
|  | Adenocarcinoma | | 2 050 206/118 | NA**^§^** | NA**^§^** | NA**^§^** | NA**^§^** | 1·53 (1·18-1·98), 0·0013 |
|  | Squamous-cell carcinoma | | 2 129 149/5062 | 1·01 (0·90-1·14), 0·89 | reference | 0·89 (0·83-0·96), 0·0034 | 0·86 (0·74-1·01), 0·074 | 0·92 (0·88-0·96), 0·001 |
| Oesophagus | | | 2 129 149/1891 | 1·09 (0·87-1·36), 0·49 | reference | 1·01 (0·90-1·13), 0·82 | 1·36 (1·11-1·66), 0·0018 | 1·08 (1·00-1·16), 0·044 |
|  | Squamous-cell carcinoma | | 2 129 149/725 | 1·62 (1·15-2·27), 0·0062 | reference | 0·48 (0·40-0·59), <0·0001 | 0·52 (0·35-0·76), 0.00091 | 0·50 (0·44-0·58), <0·0001 |
|  | Adenocarcinoma^‡^ | | 2 129 149/1067 | 0·88 (0·65-1·19), 0·39 | reference | 1·69 (1·46-1·96), <0·0001 | 2·58 (2·02-3·29), <0·0001 | 1·60 (1·47-1·75), <0·0001 |
| Gastric | | | 2 129 149/3992 | 0·99 (0·82-1·19), 0·88 | reference | 0·98 (0·91-1·06), 0·65 | 0·98 (0·85-1·13), 0·85 | 1·01 (0·96-1·06), 0·75 |
|  | Cardia^‡^ | | 2 129 149/1271 | 0·83 (0·62-1·12), 0·21 | reference | 1·39 (1·21-1·59), <0·0001 | 1·62 (1·27-2·06), <0·0001 | 1·33 (1·22-1·45), <0·0001 |
|  | Non-cardia | | 2 129 149/2697 | 1·14 (0·90-1·45), 0·28 | reference | 0·86 (0·79-0·94), 0·00093 | 0·81 (0·69-0·96), 0·020 | 0·88 (0·83-0·94), 0·00019 |
|  | Adenocarcinoma | | 2 129 149/3594 | 1·01 (0·83-1·24), 0·89 | reference | 0·97 (0·90-1·05), 0·47 | 0·93 (0·80-1·08), 0·35 | 0·98 (0·93-1·04), 0·58 |
|  | Gastrointestinal stromal | | 2 129 149/129 | NA**^§^** | NA**^§^** | NA**^§^** | NA**^§^** | 1·19 (0·90-1·59), 0·23 |
| Small intestine | | | 2 129 149/960 | 0·93 (0·69-1·26), 0·64 | reference | 1·46 (1·24-1·71), <0·0001 | 1·62 (1·20-2·19), 0·0013 | 1·32 (1·19-1·45), <0·0001 |
|  | Duodenum | | 2 129 149/189 | NA**^§^** | NA**^§^** | NA**^§^** | NA**^§^** | 1·31 (1·05-1·64), 0·019 |
|  | Ileum | | 2 087 151/216 | NA**^§^** | NA**^§^** | NA**^§^** | NA**^§^** | 1·37 (1·11-1·68), 0·0033 |
|  | Adenocarcinoma | | 2 129 149/280 | 1·13 (0·66-1·93), 0·65 | reference | 1·36 (1·01-1·81), 0·040 | 1·05 (0·56-1·96), 0·87 | 1·14 (0·94-1·38), 0·17 |
|  | Neuroendocrine | | 2 129 149/514 | 0·87 (0·57-1·32), 0·50 | reference | 1·47 (1·18-1·83), 0·00062 | 1·94 (1·31-2·86), 0·00080 | 1·38 (1·20-1·57), <0·0001 |
| Colon^‡^ | | | 2 129 149/10 873 | 0·99 (0·90-1·09), 0·81 | reference | 1·21 (1·16-1·27), <0·0001 | 1·57 (1·44-1·70), <0·0001 | 1·22 (1·19-1·26), <0·0001 |
|  | *Proximal* | | 2 129 149/5592 | 1·11 (0·97-1·26), 0·14 | reference | 1·21 (1·13-1·29), <0·0001 | 1·51 (1·35-1·69), <0·0001 | 1·20 (1·15-1·25), <0·0001 |
|  | *Distal* | | 2 129 149/4605 | 0·90 (0·77-1·05), 0·18 | reference | 1·24 (1·15-1·33), <0·0001 | 1·65 (1·46-1·87), <0·0001 | 1·26 (1·20-1·31), <0·0001 |
|  | *Adenocarcinoma* | | 2 129 149/10 269 | 0·97 (0·87-1·07), 0·52 | reference | 1·22 (1·16-1·28), <0·0001 | 1·60 (1·47-1·74), <0·0001 | 1·24 (1·20-1·27), <0·0001 |
|  | *Neuroendocrine* | | 2 129 149/421 | 1·15 (0·80-1·65), 0·46 | reference | 1·11 (0·84-1·47), 0·46 | 1·04 (0·58-1·87), 0·89 | 1·07 (0·92-1·26), 0·37 |
| Rectum/anus | | | 2 129 149/7880 | 0·96 (0·85-1·07), 0·41 | reference | 1·08 (1·02-1·14), 0·0038 | 1·26 (1·14-1·40), <0·0001 | 1·11 (1·07-1·15), <0·0001 |
|  | Rectum^‡^ | | 2 129 149/7549 | 0·97 (0·86-1·09), 0·56 | reference | 1·09 (1·03-1·15), 0·0032 | 1·27 (1·14-1·40), <0·0001 | 1·11 (1·07-1·15), <0·0001 |
|  | Anus | | 2 129 149/332 | 0·76 (0·46-1·27), 0·29 | reference | 1·02 (0·75-1·37), 0·91 | 1·15 (0·64-2·04), 0·62 | 1·18 (0·98-1·41), 0·070 |
|  | Adenocarcinoma | | 2 129 149/7364 | 0·97 (0·87-1·10), 0·65 | reference | 1·09 (1·03-1·15), 0·0035 | 1·28 (1·16-1·43), <0·0001 | 1·11 (1·07-1·16), <0·0001 |
|  | Neuroendocrine | | 2 129 149/129 | NA**^§^** | NA**^§^** | NA**^§^** | NA**^§^** | 0·94 (0·69-1·27), 0·67 |
| Liver/intrahepatic bile ducts^‡^ | | | 2 129 149/2496 | 0·96 (0·79-1·16), 0·66 | reference | 1·41 (1·28-1·56), <0·0001 | 2·85 (2·46-3·30), <0·0001 | 1·50 (1·42-1·59), <0·0001 |
| Biliary tract | | | 2 129 149/1070 | 0·93 (0·67-1·30), 0·66 | reference | 1·16 (1·00-1·34), 0·047 | 1·28 (0·98-1·67), 0·065 | 1·19 (1·08-1·31), 0·00030 |
|  | Gallbladder^‡^ | | 2 129 149/346 | 1·09 (0·60-1·98), 0·78 | reference | 1·18 (0·92-1·52), 0·18 | 1·58 (1·04-2·40), 0·030 | 1·23 (1·05-1·45), 0·012 |
|  | Extrahepatic bile ducts | | 2 129 149/412 | 0·70 (0·40-1·24), 0·22 | reference | 1·14 (0·90-1·45), 0·27 | 1·11 (0·69-1·80), 0·63 | 1·19 (1·02-1·39), 0·025 |
|  | Ampulla/papilla of Vater | | 2 129 149/163 | NA**^§^** | NA**^§^** | NA**^§^** | NA**^§^** | 1·13 (0·88-1·45), 0·33 |
| Pancreas^‡^ | | | 2 129 149/3467 | 1·01 (0·85-1·20), 0·95 | reference | 1·00 (0·92-1·08), 0·95 | 1·34 (1·16-1·54), <0·0001 | 1·12 (1·06-1·19), <0·0001 |
| Lung/bronchus | | | 2 129 149/10 906 | 1·43 (1·30-1·57), <0·0001 | reference | 0·77 (0·74-0·80), <0·0001 | 0·71 (0·65-0·78), <0·0001 | 0·76 (0·73-0·78), <0·0001 |
|  | Adenocarcinoma | | 2 129 149/3547 | 1·48 (1·28-1·70), <0·0001 | reference | 0·74 (0·68-0·80), <0·0001 | 0·77 (0·65-0·92), 0·0058 | 0·74 (0·69-0·78), <0·0001 |
|  | Squamous-cell carcinoma | | 2 129 149/2043 | 1·38 (1·08-1·76), 0·011 | reference | 0·93 (0·84-1·03), 0·17 | 0·74 (0·60-0·93), 0·010 | 0·89 (0·83-0·96), 0·0040 |
|  | Small cell | | 2 129 149/1216 | 1·25 (0·93-1·67), 0·14 | reference | 0·92 (0·80-1·05), 0·24 | 0·98 (0·75-1·27), 0·94 | 0·94 (0·86-1·04), 0·27 |
|  | Large cell | | 2 050 206/290 | 1·31 (0·73-2·34), 0·36 | reference | 0·72 (0·54-0·96), 0·024 | 0·71 (0·40-1·27), 0·25 | 0·77 (0·63-0·95), 0·012 |
| Malignant melanoma | | | 2 129 149/12 130 | 0·81 (0·75-0·88), <0·0001 | reference | 1·13 (1·08-1·19), <0·0001 | 1·15 (1·04-1·28), 0·0048 | 1·15 (1·11-1·18), <0·0001 |
|  | Acral lentiginous | | 2 050 206/135 | NA**^§^** | NA**^§^** | NA**^§^** | NA**^§^** | 1·29 (1·01-1·67), 0·047 |
|  | Superficial spreading | | 2 050 206/6639 | 0·75 (0·68-0·84), <0·0001 | reference | 1·09 (1·01-1·17), 0·019 | 1·09 (0·94-1·27), 0·26 | 1·15 (1·10-1·20), <0·0001 |
|  | Nodular | | 2 050 206/1431 | 0·81 (0·63-1·04), 0·093 | reference | 1·18 (1·03-1·35), 0·018 | 1·00 (0·75-1·35), 0·98 | 1·13 (1·03-1·23), 0·0061 |
|  | Lentigo maligna | | 2 050 206/344 | 0·90 (0·53-1·53), 0·69 | reference | 1·09 (0·83-1·42), 0·54 | 0·94 (0·52-1·71), 0·85 | 1·08 (0·90-1·30), 0·40 |
| Non-melanoma excluding basalioma | | | 2 129 149/7485 | 0·89 (0·78-1·02), 0·088 | reference | 1·00 (0·95-1·05), 0·98 | 0·96 (0·86-1·08), 0·62 | 1·00 (0·96-1·04), 0·97 |
| Breast | | | 2 129 149/253 | 0·87 (0·47-1·62), 0·66 | reference | 0·95 (0·69-1·32), 0·77 | 1·63 (0·97-2·75), 0·061 | 1·16 (0·95-1·42), 0·14 |
| Penis | | | 2 129 149/626 | 0·99 (0·69-1·44), 0·97 | reference | 1·64 (1·34-2·00), <0·0001 | 3·07 (2·28-4·14), <0·0001 | 1·54 (1·38-1·73), <0·0001 |
| Prostate | | | 2 129 149/59 118 | 0·93 (0·89-0·97), 0·00031 | reference | 0·98 (0·96-1·00), 0·072 | 0·89 (0·85-0·93), <0·0001 | 0·98 (0·96-0·99), 0·0022 |
|  | Non-aggressive | | 2 003 162/37 366 | 0·93 (0·89-0·97), 0·0021 | reference | 0·93 (0·90-0·95), <0·0001 | 0·76 (0·71-0·80), <0·0001 | 0·93 (0·91-0·94), <0·0001 |
|  | Aggressive | | 2 003 162/13 493 | 0·91 (0·83-1·01), 0·068 | reference | 1·01 (0·97-1·05), 0.65 | 0·95 (0·87-1·03), 0·20 | 1·01 (0·98-1·04), 0·51 |
| Testis | | | 2 129 149/6248 | 1·08 (0·98-1·18), 0·12 | reference | 0·97 (0·89-1·05), 0·52 | 0·93 (0·79-1·10), 0·44 | 0·95 (0·91-0·99), 0·037 |
| Renal cell^‡^ | | | 2 129 149/4554 | 0·79 (0·68-0·92), 0·0020 | reference | 1·44 (1·34-1·55), <0·0001 | 2·06 (1·82-2·32), <0·0001 | 1·44 (1·38-1·50), <0·0001 |
|  | *Clear cell* | | 1 911 072/2127 | 0·78 (0·64-0·95), 0·012 | reference | 1·72 (1·54-1·93), <0·0001 | 2·39 (1·96-2·90), <0·0001 | 1·54 (1·45-1·64), <0·0001 |
|  | *Papillary* | | 2 050 206/367 | 0·92 (0·59-1·45), 0·72 | reference | 1·32 (1·01-1·73), 0·044 | 1·20 (0·66-2·17), 0·55 | 1·19 (1·00-1·41), 0·047 |
|  | *Chromophobe* | | 1 911 072/162 | NA**^§^** | NA**^§^** | NA**^§^** | NA**^§^** | 1·40 (1·11-1·77), 0·0044 |
| Other urinary tract | | | 2 129 149/10 864 | 1·11 (1·00-1·23), 0·056 | reference | 0·94 (0·90-0·98), 0·0084 | 1·04 (0·95-1·13), 0·31 | 0·97 (0·94-1·01), 0·11 |
|  | Pelvis | | 2 129 149/461 | 1·21 (0·75-1·96), 0·43 | reference | 1·04 (0·84-1·29), 0·70 | 0·76 (0·46-1·25), 0·30 | 0·98 (0·84-1·15), 0·82 |
|  | Ureter | | 2 129 149/180 | NA**^§^** | NA**^§^** | NA**^§^** | NA**^§^** | 0·98 (0·76-1·25), 0·86 |
|  | Bladder | | 2 129 149/10 106 | 1·09 (0·98-1·21), 0·14 | reference | 0·93 (0·89-0·98), 0·0059 | 1·03 (0·95-1·13), 0·34 | 0·97 (0·94-1·01), 0·13 |
|  | Urothelial carcinoma | | 1 911 072/4873 | 1·04 (0·90-1·19), 0·62 | reference | 1·00 (0·93-1·07), 0·97 | 1·11 (0·97-1·27), 0·13 | 1·02 (0·97-1·07), 0·47 |
| Brain/central nervous system | | | 2 129 149/7143 | 0·88 (0·80-0·98), 0·015 | reference | 1·03 (0·96-1·10), 0·42 | 1·00 (0·87-1·14), 0·98 | 1·04 (1·00-1·08), 0·039 |
|  | Meningioma^‡^ | | 2 129 149/1139 | 0·74 (0·56-0·98), 0·036 | reference | 1·06 (0·91-1·25), 0·43 | 1·38 (1·03-1·83), 0·027 | 1·13 (1·03-1·24), 0·010 |
|  | Low-grade glioma | | 2 129 149/1063 | 1·02 (0·81-1·29), 0·88 | reference | 1·21 (1·02-1·44), 0·03 | 0·60 (0·36-0·99), 0·046 | 1·02 (0·92-1·12), 0·74 |
|  | High-grade glioma | | 2 129 149/2771 | 0·90 (0·77-1·06), 0·21 | reference | 1·00 (0·90-1·11), 0·99 | 1·02 (0·83-1·27), 0·79 | 1·04 (0·98-1·11), 0·16 |
| Endocrine organs | | | 2 129 149/4470 | 0·86 (0·75-0·98), 0·020 | reference | 1·25 (1·15-1·35), <0·0001 | 1·52 (1·31-1·77), <0·0001 | 1·21 (1·16-1·27), <0·0001 |
|  | Pancreatic islets | | 2 129 149/292 | 1·04 (0·65-1·65), 0·89 | reference | 1·51 (1·11-2·06), 0·0084 | 1·65 (0·90-3·00), 0·10 | 1·40 (1·18-1·66), 0·00011 |
|  | Thyroid^‡^ | | 2 129 149/1239 | 0·83 (0·65-1·07), 0·15 | reference | 1·12 (0·96-1·31), 0·16 | 1·59 (1·21-2·07), 0·00061 | 1·17 (1·07-1·28), 0·00036 |
|  |  | *Papillary* | 2 050 206/703 | 0·81 (0·58-1·12), 0·20 | reference | 1·14 (0·92-1·42), 0·23 | 2·16 (1·56-2·99), <0·0001 | 1·21 (1·08-1·36), 0·00096 |
|  |  | *Follicular* | 2 050 206/107 | NA**^§^** | NA**^§^** | NA**^§^** | NA**^§^** | 1·30 (0·98-1·74), 0·073 |
|  | Adrenal glands | | 2 129 149/249 | NA**^§^** | NA**^§^** | NA**^§^** | NA**^§^** | 1·13 (0·92-1·40), 0·23 |
|  | Parathyroid gland | | 2 129 149/976 | 0·81 (0·60-1·08), 0·15 | reference | 1·27 (1·07-1·50), 0·0048 | 1·40 (1·02-1·93), 0·035 | 1·21 (1·09-1·33), 0·0002 |
|  | Pituitary gland | | 2 129 149/1662 | 0·90 (0·73-1·11), 0·31 | reference | 1·36 (1·19-1·55), <0·0001 | 1·63 (1·27-2·09), 0·00010 | 1·25 (1·16-1·35), <0·0001 |
| Thymus | | | 2 129 149/136 | NA**^§^** | NA**^§^** | NA**^§^** | NA**^§^** | 1·10 (0·83-1·46), 0·49 |
| Pleura | | | 2 129 149/793 | 0·99 (0·60-1·63), 0·98 | reference | 0·83 (0·71-0·97), 0·022 | 0·67 (0·47-0·97), 0·038 | 0·82 (0·73-0·93), 0·0021 |
| Bone/articular cartilage | | | 2 129 149/506 | 1·20 (0·87-1·65), 0·26 | reference | 0·98 (0·75-1·28), 0·89 | 1·34 (0·83-2·17), 0·23 | 0·95 (0·82-1·11), 0·53 |
| Retroperitoneum/peritoneum | | | 2 129 149/161 | NA**^§^** | NA**^§^** | NA**^§^** | NA**^§^** | 0·97 (0·74-1·27), 0·81 |
| Connective tissue | | | 2 129 149/1364 | 0·86 (0·66-1·10), 0·22 | reference | 1·30 (1·13-1·49), 0·00018 | 1·59 (1·23-2·04), 0·00025 | 1·28 (1·17-1·38), <0·0001 |
| Eye | | | 2 129 149/560 | 0·99 (0·69-1·42), 0·94 | reference | 1·08 (0·86-1·35), 0·49 | 1·19 (0·77-1·86), 0·42 | 1·09 (0·95-1·25), 0·21 |
| Lymphoid neoplasms | | | 2 129 149/11 225 | 0·86 (0·78-0·94), 0·0010 | reference | 1·10 (1·05-1·16), <0·0001 | 1·30 (1·19-1·43), <0·0001 | 1·14 (1·11-1·18), <0·0001 |
|  | Hodkin lymphoma | | 2 129 149/1653 | 1·07 (0·89-1·29), 0·48 | reference | 1·26 (1·10-1·45), 0·00086 | 1·52 (1·18-1·96), 0·0010 | 1·20 (1·12-1·29), <0·0001 |
|  |  | Nodular sclerosis | 2 050 206/562 | 1·22 (0·90-1·67), 0·20 | reference | 1·14 (0·89-1·45), 0·31 | 1·39 (0·90-2·14), 0·14 | 1·13 (0·99-1·28), 0·064 |
|  |  | Mixed cellularity | 2 050 206/182 | NA**^§^** | NA**^§^** | NA**^§^** | NA**^§^** | 1·33 (1·08-1·63), 0·0075 |
|  |  | Nodular lymphocyte | 2 050 206/128 | NA**^§^** | NA**^§^** | NA**^§^** | NA**^§^** | 1·53 (1·21-1·94), 0·00041 |
|  | Acute lymphocytic leukaemia | | 2 129 149/379 | 0·65 (0·40-1·05), 0·077 | reference | 0·98 (0·72-1·34), 0·93 | 1·69 (1·04-2·75), 0·034 | 1·20 (1·03-1·40), 0·021 |
|  | Chronic lymphocytic leukaemia | | 2 129 149/2116 | 0·78 (0·61-1·00), 0·044 | reference | 1·02 (0·92-1·14), 0·66 | 1·13 (0·93-1·39), 0·20 | 1·08 (1·01-1·16), 0·025 |
|  | Diffuse large B-cell | | 2 050 206/1818 | 0·86 (0·69-1·08), 0·19 | reference | 1·27 (1·12-1·43), 0·00012 | 1·70 (1·37-2·10), <0·0001 | 1·29 (1·20-1·39), <0·0001 |
|  | Follicular | | 2 050 206/1111 | 0·88 (0·68-1·16), 0·37 | reference | 1·06 (0·90-1·25), 0·46 | 1·42 (1·07-1·90), 0·016 | 1·14 (1·03-1·25), 0·0084 |
|  | T-cell/natural killer-cell | | 2 050 206/623 | 0·86 (0·59-1·24), 0·41 | reference | 0·83 (0·67-1·03), 0·10 | 0·87 (0·55-1·36), 0·54 | 0·90 (0·78-1·04), 0·15 |
| Multiple myeloma^‡^ | | | 2 129 149/2573 | 0·69 (0·55-0·87), 0·0016 | reference | 1·14 (1·04-1·25), 0·0069 | 1·05 (0·87-1·28), 0·54 | 1·14 (1·07-1·22), <0·0001 |
| Myeloid neoplasms | | | 2 129 149/3101 | 0·87 (0·73-1·03), 0·10 | reference | 1·06 (0·96-1·16), 0·25 | 1·44 (1·22-1·70), <0·0001 | 1·16 (1·09-1·22), <0·0001 |
|  | Acute myeloid leukaemia | | 2 129 149/961 | 0·85 (0·62-1·17), 0·31 | reference | 1·00 (0·85-1·18), 0·99 | 1·36 (1·02-1·81), 0·035 | 1·14 (1·03-1·27), 0·0085 |
|  | Chronic myeloid leukaemia | | 2 129 149/679 | 0·64 (0·44-0·93), 0·018 | reference | 0·95 (0·76-1·18), 0·65 | 1·42 (0·97-2·08), 0·063 | 1·16 (1·03-1·31), 0·012 |

Abbreviations: BMI, body mass index.

* Hazard ratios from Cox regression models with age as time scale, adjusted for baseline age (continuous), mode of weight assessment, mode of height assessment, marital status, education level, and birth country, and stratified by calendar year of birth.

† For cancers that could only be identified by later international classification of disease (ICD) codes than ICD-7, follow-up started at the year of the start of the respective ICD edition or at baseline, whichever came later.

‡ Established obesity-related cancer.

§ The number of cancer cases was considered too low for analysis (<250 cases for categorical body mass index and <100 cases for per 5 kg/m^2^).

**Table S10. Hazard ratios (95% confidence intervals) of site-specific cancers according to body mass index level in women^*^**

| **Cancer category** | | | **No. at risk/cases^†^** | **HR (95% CI), P-value** | | | | **HR (95% CI), P-value, for Per 5 kg/m^2^ higher BMI^*^** |
| --- | --- | --- | --- | --- | --- | --- | --- | --- |
|  |  |  |  | **Underweight** | **Normal weight** | **Overweight** | **Obesity** |  |
|  |  |  |  | **(BMI<18·5 kg/m^2^)^*^** | **(BMI 18·5-24·9 kg/m^2^)^*^** | **(BMI 25-29·9 kg/m^2^)^*^** | **(BMI≥30 kg/m^2^)^*^** |  |
| Head and neck | | | 2 013 200/1910 | 0·82 (0·61-1·09), 0·16 | reference | 1·05 (0·94-1·17), 0·37 | 1·01 (0·85-1·21), 0·85 | 1·03 (0·97-1·09), 0·32 |
|  | Oral cavity | | 2 013 200/798 | 0·90 (0·58-1·41), 0·63 | reference | 1·20 (1·02-1·42), 0·030 | 1·20 (0·92-1·57), 0·16 | 1·11 (1·01-1·21), 0·025 |
|  |  | Lip | 2 013 200/163 | NA**^§^** | NA**^§^** | NA**^§^** | NA**^§^** | 1·06 (0·86-1·31), 0·57 |
|  |  | Tongue | 2 013 200/298 | 1·57 (0·89-2·76), 0·12 | reference | 1·23 (0·93-1·63), 0·14 | 1·66 (1·12-2·46), 0·010 | 1·19 (1·04-1·37), 0·012 |
|  |  | Mouth | 2 013 200/338 | 0·53 (0·22-1·30), 0·16 | reference | 1·20 (0·93-1·54), 0·16 | 0·96 (0·62-1·47), 0·85 | 1·06 (0·92-1·22), 0·44 |
|  | Salivary glands | | 2 013 200/276 | 0·23 (0·06-0·94), 0·041 | reference | 0·88 (0·66-1·18), 0·42 | 0·69 (0·41-1·16), 0·18 | 0·99 (0·84-1·16), 0·93 |
|  | Pharynx | | 2 013 200/586 | 0·77 (0·46-1·26), 0·30 | reference | 0·92 (0·75-1·14), 0·45 | 0·73 (0·50-1·07), 0·11 | 0·88 (0·78-1·00), 0·043 |
|  |  | Oropharynx | 2 013 200/466 | 0·77 (0·44-1·35), 0·36 | reference | 0·95 (0·75-1·21), 0·72 | 0·85 (0·56-1·29), 0·45 | 0·93 (0·81-1·06), 0·30 |
|  | Nasal and paranasal sinuses | | 2 013 200/138 | NA**^§^** | NA**^§^** | NA**^§^** | NA**^§^** | 1·12 (0·90-1·39), 0·26 |
|  | Larynx | | 2 013 200/108 | NA**^§^** | NA**^§^** | NA**^§^** | NA**^§^** | 1·25 (0·99-1·57), 0·062 |
|  | Adenoid cystic carcinoma | | 2 013 200/117 | NA**^§^** | NA**^§^** | NA**^§^** | NA**^§^** | 0·78 (0·60-1·03), 0·097 |
|  | Squamous-cell carcinoma | | 2 013 200/1348 | 1·00 (0·73-1·37), 0·97 | reference | 1·20 (1·05-1·36), 0·0065 | 1·21 (0·99-1·49), 0·065 | 1·08 (1·01-1·16), 0·030 |
| Oesophagus | | | 2 013 200/345 | 2·75 (1·78-4·24), <0·0001 | reference | 1·15 (0·89-1·49), 0·28 | 1·29 (0·88-1·89), 0·20 | 0·95 (0·82-1·11), 0·53 |
|  | Squamous-cell carcinoma | | 2 013 200/202 | NA**^§^** | NA**^§^** | NA**^§^** | NA**^§^** | 0·64 (0·51-0·80), <0·0001 |
|  | [Adenocarcinoma^‡^](https://en.wikipedia.org/wiki/Pilcrow) | | 2 013 200/126 | NA**^§^** | NA**^§^** | NA**^§^** | NA**^§^** | 1·64 (1·37-1·97), <0·0001 |
| Gastric | | | 2 013 200/1407 | 1·31 (0·97-1·77), 0·071 | reference | 0·96 (0·84-1·08), 0·49 | 1·02 (0·84-1·23), 0·85 | 1·01 (0·94-1·08), 0·82 |
|  | [Cardia^‡^](https://en.wikipedia.org/wiki/Pilcrow) | | 2 013 200/194 | NA**^§^** | NA**^§^** | NA**^§^** | NA**^§^** | 1·21 (1·02-1·44), 0·024 |
|  | Non-cardia | | 2 013 200/1195 | 1·38 (1·00-1·89), 0·045 | reference | 0·97 (0·84-1·11), 0·61 | 0·93 (0·75-1·15), 0·49 | 0·98 (0·91-1·06), 0·57 |
|  | Adenocarcinoma | | 2 013 200/1149 | 1·30 (0·93-1·81), 0·12 | reference | 0·90 (0·78-1·03), 0·12 | 0·94 (0·76-1·16), 0·57 | 0·98 (0·91-1·06), 0·67 |
| Small intestine | | | 2 013 200/471 | 0·47 (0·21-1·07), 0·068 | reference | 1·17 (0·94-1·45), 0·15 | 1·42 (1·03-1·95), 0·025 | 1·18 (1·05-1·32), 0·0034 |
|  | Duodenum | | 2 013 200/102 | NA**^§^** | NA**^§^** | NA**^§^** | NA**^§^** | 0·99 (0·76-1·30), 0·98 |
|  | Ileum | | 2 005 162/132 | NA**^§^** | NA**^§^** | NA**^§^** | NA**^§^** | 1·41 (1·17-1·71), 0·00037 |
|  | Adenocarcinoma | | 2 013 200/138 | NA**^§^** | NA**^§^** | NA**^§^** | NA**^§^** | 0·90 (0·71-1·14), 0·40 |
|  | Neuroendocrine | | 2 013 200/264 | 0·14 (0·02-1·01), 0·050 | reference | 1·18 (0·87-1·58), 0·26 | 2·05 (1·41-2·98), 0·00012 | 1·38 (1·20-1·59), <0·0001 |
| [Colon^‡^](https://en.wikipedia.org/wiki/Pilcrow) | | | 2 013 200/6993 | 0·99 (0·85-1·15), 0·87 | reference | 1·08 (1·02-1·14), 0·0048 | 1·19 (1·09-1·29), <0·0001 | 1·09 (1·05-1·12), <0·0001 |
|  | *Proximal* | | 2 013 200/3950 | 1·02 (0·83-1·24), 0·88 | reference | 1·12 (1·04-1·21), 0·0020 | 1·19 (1·06-1·33), 0·0027 | 1·10 (1·06-1·15), <0·0001 |
|  | *Distal* | | 2 013 200/2648 | 0·92 (0·72-1·17), 0·50 | reference | 1·03 (0·94-1·13), 0·48 | 1·21 (1·05-1·39), 0·0060 | 1·07 (1·01-1·12), 0·0081 |
|  | *Adenocarcinoma* | | 2 013 200/6532 | 0·97 (0·83-1·14), 0·73 | reference | 1·09 (1·02-1·15), 0·0056 | 1·17 (1·07-1·28), 0·00064 | 1·08 (1·05-1·12), <0·0001 |
|  | *Neuroendocrine* | | 2 013 200/353 | 1·40 (0·85-2·29), 0·18 | reference | 1·09 (0·83-1·43), 0·56 | 1·80 (1·27-2·55), 0·0010 | 1·21 (1·07-1·37), 0·0028 |
| Rectum/anus | | | 2 013 200/3764 | 1·11 (0·92-1·34), 0·28 | reference | 1·04 (0·96-1·12), 0·30 | 1·10 (0·97-1·24), 0·11 | 1·05 (1·01-1·10), 0·012 |
|  | [Rectum^‡^](https://en.wikipedia.org/wiki/Pilcrow) | | 2 013 200/3297 | 1·10 (0·90-1·34), 0·39 | reference | 1·06 (0·98-1·15), 0·14 | 1·11 (0·97-1·26), 0·10 | 1·07 (1·02-1·12), 0·0028 |
|  | Anus | | 2 013 200/469 | 1·18 (0·74-1·87), 0·50 | reference | 0·85 (0·67-1·09), 0·23 | 1·03 (0·70-1·51), 0·81 | 0·94 (0·82-1·07), 0·38 |
|  | Adenocarcinoma | | 2 013 200/3155 | 1·12 (0·91-1·37), 0·29 | reference | 1·08 (1·00-1·18), 0·064 | 1·07 (0·94-1·23), 0·31 | 1·07 (1·02-1·12), 0·0075 |
|  | Neuroendocrine | | 2 013 200/101 | NA**^§^** | NA**^§^** | NA**^§^** | NA**^§^** | 1·21 (0·94-1·56), 0·13 |
| [Liver/intrahepatic bile ducts^‡^](https://en.wikipedia.org/wiki/Pilcrow) | | | 2 013 200/788 | 0·87 (0·54-1·39), 0·56 | reference | 1·17 (0·99-1·38), 0·068 | 1·52 (1·20-1·92), 0·00041 | 1·20 (1·10-1·31), <0·0001 |
| Biliary tract | | | 2 013 200/932 | 0·54 (0·30-0·99), 0·044 | reference | 1·33 (1·14-1·54), 0·00013 | 1·61 (1·31-1·98), <0·0001 | 1·26 (1·16-1·36), <0·0001 |
|  | [Gallbladder^‡^](https://en.wikipedia.org/wiki/Pilcrow) | | 2 013 200/571 | 0·49 (0·22-1·11), 0·085 | reference | 1·37 (1·14-1·66), 0·00058 | 1·49 (1·14-1·97), 0·0022 | 1·26 (1·14-1·39), <0·0001 |
|  | Extrahepatic bile ducts | | 2 013 200/177 | NA**^§^** | NA**^§^** | NA**^§^** | NA**^§^** | 1·33 (1·12-1·59), 0·0012 |
| Pancreas^‡^ | | | 2 013 200/2042 | 1·06 (0·81-1·39), 0·68 | reference | 1·10 (0·99-1·22), 0·063 | 1·17 (1·00-1·38), 0·046 | 1·10 (1·03-1·16), 0·0013 |
| Lung/bronchus | | | 2 013 200/5950 | 1·72 (1·53-1·93), <0·0001 | reference | 0·82 (0·77-0·88), <0·0001 | 0·76 (0·68-0·85), <0·0001 | 0·81 (0·78-0·84), <0·0001 |
|  | Adenocarcinoma | | 2 013 200/3062 | 1·81 (1·55-2·12), <0·0001 | reference | 0·80 (0·73-0·88), <0·0001 | 0·72 (0·61-0·84), 0·00012 | 0·77 (0·72-0·81), <0·0001 |
|  | Squamous-cell carcinoma | | 2 013 200/649 | 1·44 (0·97-2·15), 0·069 | reference | 0·84 (0·69-1·02), 0·077 | 1·04 (0·79-1·38), 0·81 | 0·91 (0·82-1·02), 0·10 |
|  | Small cell | | 2 013 200/786 | 1·45 (1·03-2·04), 0·038 | reference | 0·98 (0·83-1·17), 0·88 | 0·90 (0·68-1·19), 0·50 | 0·94 (0·85-1·03), 0·23 |
|  | Large cell | | 1 991 973/189 | NA**^§^** | NA**^§^** | NA**^§^** | NA**^§^** | 0·64 (0·51-0·81), <0·0001 |
| Malignant melanoma | | | 2 013 200/10 037 | 0·78 (0·69-0·89), 0·00011 | reference | 0·97 (0·92-1·02), 0·26 | 0·84 (0·77-0·92), 0·00026 | 0·97 (0·95-1·00), 0·12 |
|  | Acral lentiginous | | 1 991 973/175 | NA**^§^** | NA**^§^** | NA**^§^** | NA**^§^** | 0·97 (0·80-1·19), 0·76 |
|  | Superficial spreading | | 1 991 973/6216 | 0·71 (0·60-0·84), <0·0001 | reference | 0·98 (0·92-1·04), 0·46 | 0·83 (0·74-0·92), 0·00075 | 0·97 (0·94-1·01), 0·13 |
|  | Nodular | | 1 991 973/850 | 0·74 (0·48-1·16), 0·19 | reference | 1·08 (0·91-1·28), 0·40 | 1·17 (0·90-1·53), 0·25 | 1·08 (0·99-1·18), 0·090 |
|  | Lentigo maligna | | 1 991 973/277 | 1·45 (0·77-2·76), 0·25 | reference | 1·04 (0·78-1·38), 0·80 | 0·68 (0·39-1·18), 0·17 | 0·85 (0·72-1·02), 0·078 |
| Non-melanoma excluding basalioma | | | 2 013 200/3952 | 1·10 (0·92-1·33), 0·28 | reference | 0·85 (0·79-0·92), <0·0001 | 0·80 (0·70-0·92), 0·0012 | 0·86 (0·82-0·90), <0·0001 |
| [Premenopausal breast^¶^](https://en.wikipedia.org/wiki/Pilcrow) | | | 1 925 962/24 885 | 0·99 (0·92-1·06), 0·77 | reference | 0·92 (0·89-0·95), <0·0001 | 0·72 (0·68-0·76), <0·0001 | 0·90 (0·89-0·92), <0·0001 |
| [Postmenopausal breast^‡#^](https://en.wikipedia.org/wiki/Pilcrow) | | | 687 332/19 296 | 0·98 (0·90-1·07), 0·56 | reference | 1·06 (1·03-1·10), 0·00016 | 1·09 (1·03-1·15), 0·00046 | 1·06 (1·04-1·08), <0·0001 |
| Vulva | | | 2 013 200/597 | 1·03 (0·61-1·73), 0·96 | reference | 1·75 (1·45-2·11), <0·0001 | 2·43 (1·88-3·14), <0·0001 | 1·42 (1·29-1·55), <0·0001 |
| Vagina | | | 2 013 200/101 | NA**^§^** | NA**^§^** | NA**^§^** | NA**^§^** | 1·22 (0·97-1·55), 0·087 |
| Cervix | | | 2 013 200/4650 | 1·09 (0·94-1·27), 0·28 | reference | 1·01 (0·94-1·09), 0·73 | 0·91 (0·81-1·02), 0·13 | 0·98 (0·94-1·01), 0·29 |
|  | Squamous-cell carcinoma | | 2 013 200/3336 | 1·05 (0·88-1·26), 0·61 | reference | 0·97 (0·89-1·06), 0·52 | 0·79 (0·68-0·91), 0·0022 | 0·94 (0·90-0·98), 0·013 |
|  | Adenocarcinoma | | 2 013 200/1082 | 1·15 (0·83-1·59), 0·41 | reference | 1·11 (0·95-1·29), 0·17 | 1·34 (1·08-1·65), 0·0060 | 1·10 (1·02-1·19), 0·0070 |
| Uterus | | | 2 013 200/5748 | 0·67 (0·54-0·83), 0·00015 | reference | 1·61 (1·52-1·72), <0·0001 | 3·17 (2·94-3·41), <0·0001 | 1·61 (1·57-1·65), <0·0001 |
|  | [Endometrium^‡^](https://en.wikipedia.org/wiki/Pilcrow) | | 1 991 973/2817 | 0·65 (0·49-0·87), 0·0048 | reference | 1·73 (1·59-1·90), <0·0001 | 3·33 (2·98-3·72), <0·0001 | 1·67 (1·61-1·73), <0·0001 |
|  |  | *Type I tumours* | 1 991 973/2602 | 0·68 (0·51-0·92), 0·014 | reference | 1·77 (1·61-1·94), <0·0001 | 3·35 (2·99-3·76), <0·0001 | 1·68 (1·61-1·74), <0·0001 |
|  |  | *Type II tumours* | 1 991 973/167 | NA**^§^** | NA**^§^** | NA**^§^** | NA**^§^** | 1·54 (1·30-1·83), <0·0001 |
| [Ovary^‡^](https://en.wikipedia.org/wiki/Pilcrow) | | | 2 013 200/3662 | 1·08 (0·90-1·31), 0·42 | reference | 1·09 (1·00-1·18), 0·043 | 1·09 (0·96-1·24), 0·17 | 1·05 (1·00-1·09), 0·042 |
|  | *Serous* | | 1 991 973/1566 | 1·21 (0·93-1·59), 0·16 | reference | 1·01 (0·89-1·15), 0·82 | 0·95 (0·77-1·17), 0·65 | 1·00 (0·93-1·07), 0·97 |
|  | *Mucinous* | | 1 991 973/320 | 0·91 (0·47-1·79), 0·79 | reference | 1·32 (1·01-1·71), 0·043 | 1·13 (0·73-1·76), 0·63 | 1·05 (0·91-1·22), 0·52 |
|  | *Endometrioid* | | 1 991 973/340 | 1·04 (0·55-1·97), 0·90 | reference | 1·34 (1·04-1·73), 0·024 | 1·26 (0·83-1·90), 0·27 | 1·10 (0·96-1·27), 0·17 |
|  | *Clear cell* | | 1 991 973/178 | NA**^§^** | NA**^§^** | NA**^§^** | NA**^§^** | 0·81 (0·65-1·01), 0·053 |
| [Renal cell^‡^](https://en.wikipedia.org/wiki/Pilcrow) | | | 2 013 200/1639 | 0·94 (0·68-1·29), 0·65 | reference | 1·56 (1·39-1·75), <0·0001 | 2·32 (1·99-2·70), <0·0001 | 1·46 (1·38-1·54), <0·0001 |
|  | *Clear cell* | | 1 926 796/835 | 0·73 (0·45-1·18), 0·20 | reference | 1·64 (1·40-1·92), <0·0001 | 2·38 (1·92-2·97), <0·0001 | 1·52 (1·41-1·64), <0·0001 |
|  | *Chromophobe* | | 1 926 796/120 | NA**^§^** | NA**^§^** | NA**^§^** | NA**^§^** | 1·24 (1·00-1·54), 0·050 |
| Other urinary tract | | | 2 013 200/2271 | 0·97 (0·70-1·33), 0·85 | reference | 1·01 (1·1-1·43), 0·89 | 0·98 (1·27-1·86), 0·83 | 0·99 (0·93-1·05), 0·70 |
|  | Pelvis | | 2 013 200/168 | NA**^§^** | NA**^§^** | NA**^§^** | NA**^§^** | 0·85 (0·68-1·06), 0·16 |
|  | Bladder | | 2 013 200/1999 | 0·92 (0·69-1·22), 0·54 | reference | 1·03 (0·92-1·14), 0·57 | 1·02 (0·86-1·21), 0·79 | 1·00 (0·94-1·06), 0·91 |
|  | Urothelial carcinoma | | 1 926 796/1249 | 1·01 (0·72-1·42), 0·89 | reference | 1·02 (0·89-1·17), 0·81 | 1·00 (0·80-1·25), 0·94 | 1·02 (0·94-1·10), 0·66 |
| Brain/central nervous system | | | 2 013 200/5466 | 1·06 (0·91-1·23), 0·46 | reference | 1·06 (0·99-1·13), 0·078 | 1·02 (0·92-1·14), 0·59 | 1·03 (0·99-1·07), 0·077 |
|  | [Meningioma^‡^](https://en.wikipedia.org/wiki/Pilcrow) | | 2 013 200/2560 | 1·16 (0·94-1·43), 0·18 | reference | 1·15 (1·05-1·27), 0·0030 | 1·18 (1·01-1·37), 0·031 | 1·10 (1·05-1·16), 0·00015 |
|  | Low-grade glioma | | 2 013 200/520 | 1·07 (0·68-1·68), 0·78 | reference | 0·90 (0·72-1·13), 0·39 | 0·80 (0·55-1·16), 0·25 | 0·96 (0·85-1·08), 0·50 |
|  | High-grade glioma | | 2 013 200/1219 | 0·81 (0·56-1·16), 0·23 | reference | 1·08 (0·94-1·24), 0·28 | 0·91 (0·72-1·16), 0·49 | 1·01 (0·94-1·10), 0·65 |
| Endocrine organs | | | 2 013 200/6754 | 0·90 (0·78-1·04), 0·14 | reference | 1·14 (1·07-1·21), <0·0001 | 1·28 (1·18-1·40), <0·0001 | 1·11 (1·08-1·15), <0·0001 |
|  | Pancreatic islets | | 2 013 200/188 | NA**^§^** | NA**^§^** | NA**^§^** | NA**^§^** | 1·03 (0·86-1·25), 0·69 |
|  | [Thyroid^‡^](https://en.wikipedia.org/wiki/Pilcrow) | | 2 013 200/2867 | 0·99 (0·80-1·22), 0·90 | reference | 1·11 (1·01-1·21), 0·025 | 1·11 (0·97-1·28), 0·12 | 1·06 (1·02-1·11), 0·0055 |
|  |  | *Papillary* | 1 991 973/2106 | 1·01 (0·79-1·28), 0·94 | reference | 1·09 (0·98-1·21), 0·12 | 1·12 (0·95-1·31), 0·17 | 1·06 (1·00-1·12), 0·040 |
|  |  | *Follicular* | 1 991 973/301 | 1·00 (0·51-1·96), 0.99 | reference | 1·28 (0·98-1·68), 0·073 | 1·25 (0·83-1·91), 0·29 | 1·15 (1·00-1·32), 0·053 |
|  | Adrenal glands | | 2 013 200/230 | NA**^§^** | NA**^§^** | NA**^§^** | NA**^§^** | 1·19 (1·02-1·40), 0·030 |
|  | Parathyroid gland | | 2 013 200/2168 | 0·82 (0·62-1·07), 0·13 | reference | 1·09 (0·98-1·21), 0·084 | 1·40 (1·20-1·63), <0·0001 | 1·15 (1·09-1·21), <0·0001 |
|  | Pituitary gland | | 2 013 200/1274 | 0·96 (0·70-1·33), 0·78 | reference | 1·25 (1·10-1·43), 0·00072 | 1·54 (1·27-1·86), <0·0001 | 1·16 (1·08-1·24), <0·0001 |
| Bone/articular cartilage | | | 2 013 200/181 | NA**^§^** | NA**^§^** | NA**^§^** | NA**^§^** | 1·07 (0·88-1·29), 0·48 |
| Retroperitoneum/peritoneum | | | 2 013 200/231 | NA**^§^** | NA**^§^** | NA**^§^** | NA**^§^** | 1·09 (0·92-1·30), 0·33 |
| Connective tissue | | | 2 013 200/665 | 0·60 (0·34-1·07), 0·084 | reference | 1·19 (1·00-1·43), 0·054 | 1·10 (0·82-1·47), 0·51 | 1·11 (1·01-1·23), 0·027 |
| Eye | | | 2 013 200/350 | 0·64 (0·30-1·37), 0·24 | reference | 0·88 (0·67-1·16), 0·41 | 1·34 (0·91-1·95), 0·10 | 1·05 (0·91-1·21), 0·39 |
| Lymphoid neoplasms | | | 2 013 200/4793 | 0·98 (0·83-1·17), 0·85 | reference | 1·08 (1·00-1·15), 0·037 | 1·12 (1·01-1·25), 0·039 | 1·08 (1·04-1·12), <0·0001 |
|  | Hodgkin lymphoma | | 2 013 200/586 | 0·96 (0·60-1·52), 0·85 | reference | 1·17 (0·96-1·43), 0·12 | 1·23 (0·91-1·66), 0·18 | 1·15 (1·04-1·26), 0·0061 |
|  |  | Nodular sclerosis | 1 991 973/318 | 0·95 (0·50-1·79), 0·89 | reference | 1·06 (0·81-1·40), 0·70 | 0·81 (0·52-1·28), 0·38 | 1·02 (0·89-1·17), 0·83 |
|  | Acute lymphocytic leukaemia | | 2 013 200/160 | NA**^§^** | NA**^§^** | NA**^§^** | NA**^§^** | 1·17 (0·97-1·41), 0·092 |
|  | Chronic lymphocytic leukaemia | | 2 013 200/738 | 1·17 (0·77-1·78), 0·47 | reference | 1·05 (0·88-1·25), 0·52 | 0·90 (0·66-1·21), 0·52 | 1·01 (0·91-1·12), 0·75 |
|  | Diffuse large B-cell | | 1 991 973/957 | 1·07 (0·73-1·56), 0·77 | reference | 1·25 (1·08-1·46), 0·0027 | 1·37 (1·08-1·72), 0·0055 | 1·16 (1·07-1·26), 0·00013 |
|  | Follicular | | 1 991 973/820 | 1·00 (0·67-1·50), 0·99 | reference | 1·03 (0·87-1·22), 0·76 | 0·98 (0·74-1·30), 0·83 | 1·04 (0·95-1·14), 0·41 |
|  | T-cell/natural killer-cell | | 1 991 973/355 | 1·20 (0·67-2·14), 0·54 | reference | 0·98 (0·75-1·27), 0·83 | 1·15 (0·78-1·68), 0·54 | 1·09 (0·95-1·24), 0·26 |
| [Multiple myeloma^‡^](https://en.wikipedia.org/wiki/Pilcrow) | | | 2 013 200/1147 | 0·95 (0·64-1·39), 0·75 | reference | 1·25 (1·09-1·43), 0·0012 | 1·20 (0·97-1·49), 0·076 | 1·12 (1·04-1·21), 0·0014 |
| Myeloid neoplasms | | | 2 013 200/1683 | 0·88 (0·65-1·20), 0·43 | reference | 1·05 (0·93-1·18), 0·42 | 1·39 (1·17-1·64), <0·0001 | 1·14 (1·07-1·21), <0·0001 |
|  | Acute myeloid leukaemia | | 2 013 200/537 | 0·69 (0·38-1·26), 0·21 | reference | 1·03 (0·83-1·56), 0·73 | 1·13 (0·83-1·56), 0·35 | 1·12 (1·01-1·25), 0·022 |
|  | Chronic myeloid leukaemia | | 2 013 200/323 | 1·08 (0·59-1·99), 0·82 | reference | 1·06 (1·80-1·40), 0·67 | 1·55 (1·06-2·27), 0·022 | 1·19 (1·04-1·36), 0·011 |

Abbreviations: BMI, body mass index.

* Hazard ratios from Cox regression models with age as time scale, adjusted for baseline age (continuous), weight assessment from the Medical Birth Register (yes/no), mode of weight assessment, mode of height assessment, marital status, education level, and birth country, and stratified by calendar year of birth.

† For cancers that could only be identified by later international classification of disease (ICD) codes than ICD-7, follow-up started at the year of the start of the respective ICD edition or at baseline, whichever came later.

‡ Established obesity-related cancer.

§ The number of cancer cases was considered too low for analysis (<250 cases for categorical body mass index and <100 cases for per 5 kg/m^2^).

[¶](https://en.wikipedia.org/wiki/Pilcrow) Person-years were counted from the date of health examination until the diagnosis of breast cancer, or until censoring due to another cancer, death, emigration, or until the 55^th^ birthday, whichever came first.

# Person-years were counted from age 55 years onwards or from the date of health examination if this occurred after the 55^th^ birthday.

**Table S11. Summary of findings for each cancer and its inclusion or exclusion as a potential obesity-related cancer**

| Cancer category | | | X for a significant finding (P-value < 0·05)^#^ | | | | |  | X for an established obesity-related cancer |  | X for inclusion as potential obesity-related  (excludes established obesity-related cancers) | | | | |
| --- | --- | --- | --- | --- | --- | --- | --- | --- | --- | --- | --- | --- | --- | --- | --- |
|  |  |  | **HR for obesity vs. normal weight or Per 5 kg/m^2^ higher BMI** | | | **Sex interaction** | **Heterogeneity** |  |  |  | **Both sex** | | **Men** | **Women** | |
|  |  |  | **Both sex** | **Men** | **Women** |  |  |  |  |  |  |  |  |  |  |
| Head and neck | | |  |  |  |  |  |  |  |  |  |  | | |  |
|  | Oral cavity | | X |  | X |  |  |  |  |  | X |  | | |  |
|  |  | Lip | X | X |  | X |  |  |  |  |  |  | | |  |
|  |  | Tongue | X |  | X |  |  |  |  |  |  |  | | |  |
|  |  | Mouth |  |  |  |  |  |  |  |  |  |  | | |  |
|  | Salivary glands | |  |  |  |  |  |  |  |  |  |  | | |  |
|  | Pharynx | |  |  |  |  |  |  |  |  |  |  | | |  |
|  |  | Oropharynx |  |  |  |  |  |  |  |  |  |  | | |  |
|  |  | Nasopharynx |  |  |  |  |  |  |  |  |  |  | | |  |
|  |  | Hypopharynx |  |  |  |  |  |  |  |  |  |  | | |  |
|  | Nasal and paranasal sinuses | | X | X |  |  |  |  |  |  | X |  | | |  |
|  | Larynx | |  |  |  | X |  |  |  |  |  |  | | |  |
|  | Adenoid cystic carcinoma | |  |  |  |  |  |  |  |  |  |  | | |  |
|  | Mucoepidermoid carcinoma | |  |  |  |  |  |  |  |  |  |  | | |  |
|  | Adenocarcinoma | | X | X |  | X |  |  |  |  |  | X | | |  |
|  | Squamous-cell carcinoma | |  |  | X | X |  |  |  |  |  |  | | | X |
| Oesophagus | | |  | X |  | X |  |  |  |  |  |  | | |  |
|  | Squamous-cell carcinoma | |  |  |  |  |  |  |  |  |  |  | | |  |
|  | Adenocarcinoma | | X | X | X |  |  |  | X |  |  |  | | |  |
| Gastric | | |  |  |  |  |  |  |  |  |  |  | | |  |
|  | Cardia | | X | X | X |  |  |  | X |  |  |  | | |  |
|  | Non-cardia | |  |  |  |  |  |  |  |  |  |  | | |  |
|  | Adenocarcinoma | |  |  |  |  | X |  |  |  |  |  | | |  |
|  | Neuroendocrine | |  |  |  |  |  |  |  |  |  |  | | |  |
|  | Gastrointestinal stromal | | X |  |  |  |  |  |  |  | X |  | | |  |
| Small intestine | | | X | X | X |  |  |  |  |  | X |  | | |  |
|  | Duodenum | |  | X |  |  |  |  |  |  |  |  | | |  |
|  | Jejunum | |  |  |  |  |  |  |  |  |  |  | | |  |
|  | Ileum | | X | X | X |  |  |  |  |  |  |  | | |  |
|  | Adenocarcinoma | |  |  |  |  | X |  |  |  |  |  | | |  |
|  | Neuroendocrine | | X | X | X |  |  |  |  |  |  |  | | |  |
|  | Gastrointestinal stromal | |  |  |  |  |  |  |  |  |  |  | | |  |
| Colon | | | X | X | X | X |  |  | X |  |  |  | | |  |
|  | Proximal | | X | X | X | X |  |  |  |  |  |  | | |  |
|  | Distal | | X | X | X | X |  |  |  |  |  |  | | |  |
|  | Adenocarcinoma | | X | X | X | X |  |  |  |  |  |  | | |  |
|  | Neuroendocrine | | X |  | X |  |  |  |  |  |  |  | | |  |
| Rectum/anus | | | X | X | X | X |  |  |  |  |  |  | | |  |
|  | Rectum | | X | X | X | X |  |  | X |  |  |  | | |  |
|  | Anus | |  |  |  | X |  |  |  |  |  |  | | |  |
|  | Adenocarcinoma | | X | X | X |  |  |  |  |  |  |  | | |  |
|  | Neuroendocrine^†^ | |  |  |  |  |  |  |  |  |  |  | | |  |
| Liver/intrahepatic bile ducts | | | X | X | X | X |  |  | X |  |  |  | | |  |
| Biliary tract | | | X | X | X |  |  |  |  |  | X |  | | |  |
|  | Gallbladder | | X | X | X |  |  |  | X |  |  |  | | |  |
|  | Extrahepatic bile ducts | | X | X | X |  |  |  |  |  |  |  | | |  |
|  | Ampulla/papilla of Vater | |  |  |  |  |  |  |  |  |  |  | | |  |
| Pancreas | | | X | X | X |  |  |  | X |  |  |  | | |  |
| Lung/bronchus | | |  |  |  | X |  |  |  |  |  |  | | |  |
|  | Adenocarcinoma | |  |  |  | X |  |  |  |  |  |  | | |  |
|  | Squamous-cell carcinoma | |  |  |  |  |  |  |  |  |  |  | | |  |
|  | Small cell | |  |  |  |  |  |  |  |  |  |  | | |  |
|  | Large cell | |  |  |  |  |  |  |  |  |  |  | | |  |
| Malignant melanoma | | | X | X |  | X |  |  |  |  |  | X | | |  |
|  | Acral lentiginous | |  | X |  |  |  |  |  |  |  |  | | |  |
|  | Superficial spreading | | X | X |  | X |  |  |  |  |  |  | | |  |
|  | Nodular | | X | X |  |  |  |  |  |  | X |  | | |  |
|  | Lentigo maligna | |  |  |  |  |  |  |  |  |  |  | | |  |
| Non-melanoma excluding basalioma | | |  |  |  | X |  |  |  |  |  |  | | |  |
| Breast, male | | |  |  |  |  |  |  |  |  |  |  | | |  |
| Breast, premenopausal | | |  |  |  | NA |  |  |  |  |  |  | | |  |
| Breast, postmenopausal | | |  |  | X | NA |  |  | X |  |  |  | | |  |
| Vulva | | |  |  | X | NA |  |  |  |  |  |  | | | X |
| Vagina | | |  |  |  | NA |  |  |  |  |  |  | | |  |
| Cervix | | |  |  |  | NA |  |  |  |  |  |  | | |  |
|  | Squamous-cell carcinoma | |  |  |  | NA | X |  |  |  |  |  | | |  |
|  | Adenocarcinoma | |  |  | X | NA |  |  |  |  |  |  | | | X |
| Uterus | | |  |  | X | NA |  |  |  |  |  |  | | |  |
|  | Endometrium | |  |  | X | NA |  |  | X |  |  |  | | |  |
|  |  | Type I tumours |  |  | X | NA |  |  |  |  |  |  | | |  |
|  |  | Type II tumours |  |  | X | NA |  |  |  |  |  |  | | |  |
| Ovary | | |  |  | X | NA |  |  | X |  |  |  | | |  |
|  | Serous | |  |  |  | NA |  |  |  |  |  |  | | |  |
|  | Mucinous | |  |  |  | NA |  |  |  |  |  |  | | |  |
|  | Endometrioid | |  |  |  | NA |  |  |  |  |  |  | | |  |
|  | Clear cell | |  |  |  | NA |  |  |  |  |  |  | | |  |
| Penis | | |  | X |  | NA |  |  |  |  |  | X | | |  |
| Prostate | | |  |  |  | NA |  |  |  |  |  |  | | |  |
|  | Non-aggressive | |  |  |  | NA |  |  |  |  |  |  | | |  |
|  | Aggressive | |  |  |  | NA |  |  |  |  |  |  | | |  |
| Testis | | |  |  |  | NA |  |  |  |  |  |  | | |  |
| Renal cell | | | X | X | X |  |  |  | X |  |  |  | | |  |
|  | Clear cell | | X | X | X |  | X |  |  |  |  |  | | |  |
|  | Papillary | | X | X |  |  |  |  |  |  |  |  | | |  |
|  | Chromophobe | | X | X |  |  |  |  |  |  |  |  | | |  |
| Other urinary tract | | |  |  |  |  |  |  |  |  |  |  | | |  |
|  | Pelvis | |  |  |  |  |  |  |  |  |  |  | | |  |
|  | Ureter | |  |  |  |  |  |  |  |  |  |  | | |  |
|  | Bladder | |  |  |  |  |  |  |  |  |  |  | | |  |
|  | Urothelial carcinoma | |  |  |  |  |  |  |  |  |  |  | | |  |
|  | Squamous-cell carcinoma | |  |  |  |  |  |  |  |  |  |  | | |  |
| Brain/central nervous system | | | X | X |  |  |  |  |  |  |  |  | | |  |
|  | Meningioma | | X | X | X |  | X |  | X |  |  |  | | |  |
|  | Low-grade glioma | |  |  |  |  |  |  |  |  |  |  | | |  |
|  | High-grade glioma | |  |  |  |  |  |  |  |  |  |  | | |  |
| Endocrine organs | | | X | X | X | X |  |  |  |  |  |  | | |  |
|  | Pancreatic islets | | X | X |  |  |  |  |  |  | X |  | | |  |
|  | Thyroid | | X | X | X | X |  |  | X |  |  |  | | |  |
|  |  | Papillary | X | X | X | X | X |  |  |  |  |  | | |  |
|  |  | Follicular | X |  |  |  |  |  |  |  |  |  | | |  |
|  | Adrenal glands | | X |  | X |  |  |  |  |  | X |  | | |  |
|  | Parathyroid gland | | X | X | X |  |  |  |  |  | X |  | | |  |
|  | Pituitary gland | | X | X | X |  |  |  |  |  | X |  | | |  |
| Thymus | | |  |  |  |  |  |  |  |  |  |  | | |  |
| Mediastinum | | |  |  |  |  |  |  |  |  |  |  | | |  |
| Pleura | | |  |  |  |  |  |  |  |  |  |  | | |  |
| Bone/articular cartilage | | |  |  |  |  |  |  |  |  |  |  | | |  |
| Retroperitoneum/peritoneum | | |  |  |  |  |  |  |  |  |  |  | | |  |
| Connective tissue | | | X | X | X | X |  |  |  |  | X |  | | |  |
| Eye | | |  |  |  |  |  |  |  |  |  |  | | |  |
| Lymphoid neoplasms | | | X | X | X | X |  |  |  |  | X |  | | |  |
|  | Acute lymphocytic leukaemia | | X | X |  |  | X  (in men) |  |  |  |  |  | | |  |
|  | Chronic lymphocytic leukaemia | | X | X |  |  |  |  |  |  |  |  | | |  |
|  | Diffuse large B-cell | | X | X | X |  |  |  |  |  |  |  | | |  |
|  | Follicular | | X | X |  |  |  |  |  |  |  |  | | |  |
|  | T-cell/natural killer-cell | |  |  |  | X |  |  |  |  |  |  | | |  |
|  | Hodgkin lymphoma^‡^ | | X | X | X |  |  |  |  |  |  |  | | |  |
|  |  | Nodular sclerosis |  |  |  |  | X |  |  |  |  |  | | |  |
|  |  | Mixed cellularity | X | X |  |  |  |  |  |  |  |  | | |  |
|  |  | Nodular lymphocyte | X | X |  |  |  |  |  |  |  |  | | |  |
| Multiple myeloma | | | X | X | X |  |  |  | X |  |  |  | | |  |
| Myeloid neoplasms | | | X | X | X |  |  |  |  |  | X |  | | |  |
|  | Acute myeloid | | X | X | X |  |  |  |  |  |  |  | | |  |
|  | Chronic myeloid | | X | X | X |  |  |  |  |  |  |  | | |  |

Abbreviations: HR, hazard ratio; BMI, body mass index.

† We assumed that the association between higher BMI and rectum/anus-neuroendocrine carcinoma is mostly driven by the association between higher BMI and rectal cancer which is an established obesity-related cancer, and therefore we did not include rectum/anus-neuroendocrine carcinoma as a potential obesity-related cancer.

‡ For lymphoid neoplasms and Hodgkin lymphoma, results for heterogeneity were not taken into account when considering the full cancer as potentially obesity-related because the subgroups made up less than 75% of the cancer.

# The investigation of 122 cancers and cancer subtypes increases the potential of false positive findings. For analyses of men and women combined, we found significant positive associations for 32 out of 78 cancer endpoints (excluding established obesity-related cancers); the expected value of false positives is 3.9, and the likelihood of more than 7 false positives is less than 5%. For men, there were 32 significant positive associations out of 79 cancer endpoints; the expected value of false positives is 3.95, and the likelihood of more than 7 false positives is less than 5%. For women, there were 21 significant positive associations out of 67 cancer endpoints; the expected value of false positives is 3.35, and the likelihood of more than 6 false positives is less than 5%.

**Table S12. Hazard ratios (95% confidence intervals) of smoking-related cancers according to body mass index level with results shown for smoking unadjusted analysis of all individuals, smoking unadjusted and adjusted analysis of individuals with smoking information, and for never smokers^*^**

| **Cancer category** | | | **All individuals (N=4 142 349, smoking not adjusted)** | | |  | **Smoking status available (N=802 843)** | | | | | |  | **Never smoker (N=394 435)** | | | |
| --- | --- | --- | --- | --- | --- | --- | --- | --- | --- | --- | --- | --- | --- | --- | --- | --- | --- |
|  |  |  | **No. at risk/**  **cases^†^** | **Obesity vs. normal weight^*^** | **Per 5 kg/m^2^ higher BMI^*^** |  | **No. at risk/**  **cases^†^** | **Smoking not adjusted** | |  | **Smoking adjusted^‡^** | |  | **No. at risk/**  **cases^†^** | **Obesity vs.**  **normal weight^*^** | | **Per 5 kg/m^2^ higher BMI^*^** |
|  |  |  |  |  |  |  |  | **Obesity vs. normal weight^*^** | **Per 5 kg/m^2^ higher BMI^*^** |  | **Obesity vs. normal weight^*^** | **Per 5 kg/m^2^ higher BMI^*^** |  |  |  |  |  |
| Head and neck | | | 4 142 349/7853 | 0·94 (0·84-1·05) | 0·97 (0·94-1·01) |  | 802 843/3508 | 0·90 (0·77-1·04) | 0·93 (0·88-0·99) |  | 0·93 (0·80-1·09) | 0·96 (0·91-1·02) |  | 394 435/990 | | 0·97 (0·72-1·29) | 1·05 (0·95-1·15) |
|  | Oral cavity | | 4 142 349/2688 | 1·11 (0·94-1·32) | 1·06 (1·00-1·12) |  | 802 843/1284 | 1·07 (0·85-1·35) | 1·06 (0·97-1·15) |  | 1·10 (0·88-1·39) | 1·08 (0·99-1·17) |  | 394 435/432 | | 1·14 (0·76-1·70) | 1·19 (1·03-1·36) |
|  |  | Tongue | 4 142 349/925 | 1·45 (1·09-1·91) | 1·14 (1·04-1·26) |  | 802 843/340 | 1·49 (0·99-2·25) | 1·17 (1·00-1·38) |  | 1·53 (1·02-2·30) | 1·19 (1·02-1·40) |  | 394 435/112 | | NA^§^ | 1·27 (0·97-1·65) |
|  |  | Mouth | 4 142 349/1005 | 0·85 (0·62-1·16) | 0·95 (0·86-1·05) |  | 802 843/479 | 0·77 (0·51-1·17) | 0·90 (0·78-1·04) |  | 0·81 (0·54-1·24) | 0·94 (0·82-1·09) |  | 394 435/147 | | NA^§^ | 1·18 (0·93-1·49) |
|  | Pharynx | | 4 142 349/2982 | 0·71 (0·57-0·90) | 0·88 (0·83-0·94) |  | 802 843/1102 | 0·69 (0·50-0·96) | 0·82 (0·74-0·91) |  | 0·72 (0·52-0·99) | 0·84 (0·76-0·94) |  | 394 435/302 | | 0·89 (0·49-1·62) | 0·90 (0·74-1·09) |
|  |  | Oropharynx | 4 142 349/2386 | 0·90 (0·71-1·15) | 0·95 (0·89-1·02) |  | 802 843/812 | 0·88 (0·61-1·26) | 0·93 (0·82-1·04) |  | 0·90 (0·62-1·29) | 0·94 (0·84-1·06) |  | 394 435/250 | | 0·87 (0·44-1·74) | 0·90 (0·72-1·12) |
|  |  | Nasopharynx | 4 142 349/300 | 0·54 (0·27-1·05) | 0·86 (0·72-1·03) |  | 802 843/107 | NA^§^ | 0·87 (0·63-1·19) |  | NA^§^ | 0·89 (0·65-1·22) |  | 394 435/30 | | NA^§^ | NA^§^ |
|  |  | Hypopharynx | 4 142 349/300 | 0·10 (0·02-0·39) | 0·48 (0·39-0·59) |  | 802 843/184 | NA^§^ | 0·47 (0·36-0·61) |  | NA^§^ | 0·51 (0·40-0·67) |  | 394 435/22 | | NA^§^ | NA^§^ |
|  | Nasal and paranasal sinuses | | 4 142 349/431 | 1·55 (1·06-2·26) | 1·16 (1·01-1·33) |  | 802 843/200 | NA^§^ | 0·99 (0·80-1·23) |  | NA^§^ | 1·02 (0·82-1·26) |  | 394 435/65 | | NA^§^ | NA^§^ |
|  | Larynx | | 4 142 349/1052 | 0·90 (0·68-1·19) | 0·87 (0·79-0·97) |  | 802 843/637 | 0·85 (0·61-1·19) | 0·82 (0·72-0·94) |  | 0·92 (0·66-1·29) | 0·88 (0·77-1·00) |  | 394 435/83 | | NA^§^ | NA^§^ |
|  | Adenocarcinoma | | 4 042 179/208 | NA^§^ | 1·26 (1·04-1·52) |  | 760 772/100 | NA^§^ | 1·35 (1·03-1·78) |  | NA^§^ | 1·38 (1·05-1·80) |  | 380 344/39 | | NA^§^ | NA^§^ |
|  | Squamous-cell carcinoma | | 4 142 349/6410 | 0·96 (0·85-1·08) | 0·96 (0·93-1·00) |  | 802 843/2950 | 0·91 (0·77-1·07) | 0·92 (0·87-0·98) |  | 0·95 (0·80-1·11) | 0·95 (0·90-1·01) |  | 394 435/784 | | 0·91 (0·65-1·28) | 1·02 (0·91-1·15) |
| Oesophagus | | | 4 142 349/2236 | 1·34 (1·12-1·59) | 1·05 (0·98-1·12) |  | 802 843/1308 | 1·32 (1·07-1·64) | 1·07 (0·98-1·16) |  | 1·39 (1·12-1·72) | 1·11 (1·02-1·21) |  | 394 435/313 | | 1·55 (1·00-2·40) | 1·34 (1·14-1·57) |
|  | Squamous-cell carcinoma | | 4 142 349/927 | 0·52 (0·37-0·73) | 0·54 (0·48-0·61) |  | 802 843/557 | 0·56 (0·38-0·83) | 0·53 (0·46-0·62) |  | 0·62 (0·42-0·91) | 0·58 (0·50-0·67) |  | 394 435/114 | | NA^§^ | 0·61 (0·44-0·85) |
|  | Adenocarcinoma | | 4 142 349/1193 | 2·73 (2·20-3·40) | 1·61 (1·49-1·74) |  | 802 843/680 | 2·64 (2·01-3·47) | 1·68 (1·52-1·87) |  | 2·67 (2·03-3·51) | 1·70 (1·53-1·88) |  | 394 435/178 | | NA^§^ | 1·93 (1·61-2·32) |
| Gastric | | | 4 142 349/5399 | 0·99 (0·89-1·11) | 1·01 (0·97-1·05) |  | 802 843/3083 | 0·87 (0·75-1·01) | 0·92 (0·87-0·97) |  | 0·91 (0·78-1·05) | 0·94 (0·89-1·00) |  | 394 435/1048 | | 1·01 (0·79-1·29) | 1·02 (0·93-1·12) |
|  | Cardia | | 4 142 349/1465 | 1·64 (1·32-2·03) | 1·31 (1·21-1·41) |  | 802 843/791 | 1·32 (0·99-1·76) | 1·19 (1·07-1·32) |  | 1·37 (1·03-1·82) | 1·22 (1·09-1·35) |  | 394 435/198 | | NA^§^ | 1·13 (0·91-1·41) |
|  | Adenocarcinoma | | 4 142 349/4743 | 0·93 (0·83-1·05) | 0·98 (0·94-1·03) |  | 802 843/2781 | 0·86 (0·73-1·00) | 0·90 (0·85-0·96) |  | 0·89 (0·76-1·04) | 0·93 (0·88-0·99) |  | 394 435/923 | | 0·90 (0·69-1·17) | 0·98 (0·89-1·08) |
| Liver/intrahepatic bile ducts | | | 4 142 349/3284 | 2·35 (2·08-2·67) | 1·40 (1·33-1·47) |  | 802 843/1619 | 2·64 (2·26-3·09) | 1·53 (1·43-1·63) |  | 2·73 (2·33-3·19) | 1·56 (1·46-1·66) |  | 394 435/479 | | 2·78 (2·10-3·68) | 1·59 (1·42-1·78) |
| Pancreas | | | 4 142 349/5509 | 1·26 (1·13-1·40) | 1·11 (1·07-1·16) |  | 802 843/2974 | 1·35 (1·18-1·54) | 1·11 (1·06-1·18) |  | 1·40 (1·23-1·60) | 1·14 (1·08-1·20) |  | 394 435/1021 | | 1·63 (1·32-2·02) | 1·20 (1·10-1·32) |
| Lung/bronchus | | | 4 142 349/16 856 | 0·72 (0·67-0·78) | 0·78 (0·76-0·79) |  | 802 843/10 021 | 0·70 (0·64-0·76) | 0·76 (0·73-0·78) |  | 0·77 (0·71-0·84) | 0·82 (0·80-0·85) |  | 394 435/1314 | | 0·61 (0·47-0·78) | 0·75 (0·69-0·83) |
|  | Adenocarcinoma | | 4 142 349/6609 | 0·74 (0·65-0·83) | 0·75 (0·72-0·78) |  | 802 843/3556 | 0·74 (0·63-0·86) | 0·76 (0·72-0·81) |  | 0·80 (0·68-0·93) | 0·81 (0·77-0·86) |  | 394 435/575 | | 0·89 (0·63-1·25) | 0·83 (0·73-0·95) |
|  | Squamous-cell carcinoma | | 4 142 349/2692 | 0·84 (0·70-0·99) | 0·90 (0·84-0·96) |  | 802 843/1882 | 0·78 (0·64-0·96) | 0·88 (0·82-0·95) |  | 0·88 (0·72-1·08) | 0·97 (0·90-1·04) |  | 394 435/168 | | NA^§^ | 0·71 (0·54-0·92) |
|  | Small cell | | 4 142 349/2002 | 0·94 (0·77-1·14) | 0·94 (0·88-1·01) |  | 802 843/1155 | 0·93 (0·73-1·17) | 0·92 (0·83-1·00) |  | 1·06 (0·84-1·34) | 1·01 (0·92-1·10) |  | 394 435/85 | | NA^§^ | NA^§^ |
|  | Large cell | | 4 042 179/479 | 0·66 (0·43-1·01) | 0·71 (0·61-0·83) |  | 760 772/290 | 0·66 (0·40-1·10) | 0·71 (0·58-0·86) |  | 0·73 (0·44-1·20) | 0·76 (0·63-0·92) |  | 380 344/35 | | NA^§^ | NA^§^ |
| Cervix | | | 2 013 200/4650 | 0·91 (0·81-1·02) | 0·98 (0·94-1·01) |  | 290 323/717 | 0·87 (0·64-1·19) | 0·94 (0·84-1·04) |  | 0·88 (0·65-1·20) | 0·95 (0·85-1·05) |  | 159 775/333 | | 0·93 (0·60-1·45) | 0·96 (0·82-1·12) |
|  | Squamous-cell carcinoma | | 2 013 200/3336 | 0·79 (0·68-0·91) | 0·94 (0·90-0·98) |  | 290 323/540 | 0·60 (0·40-0·90) | 0·85 (0·75-0·97) |  | 0·61 (0·41-0·92) | 0·87 (0·76-0·98) |  | 159 775/242 | | NA^§^ | 0·93 (0·78-1·12) |
| Other urinary tract | | | 4 142 349/13 135 | 1·02 (0·95-1·10) | 0·98 (0·95-1·01) |  | 802 843/8302 | 1·09 (1·00-1·19) | 0·98 (0·95-1·02) |  | 1·11 (1·02-1·22) | 1·01 (0·97-1·04) |  | 394 435/2089 | | 0·90 (0·74-1·09) | 0·96 (0·89-1·03) |
|  | Pelvis | | 4 142 349/629 | 0·75 (0·51-1·11) | 0·93 (0·82-1·06) |  | 802 843/397 | 0·87 (0·56-1·36) | 0·98 (0·84-1·15) |  | 0·90 (0·57-1·40) | 1·00 (0·85-1·17) |  | 394 435/126 | | NA^§^ | 0·73 (0·54-0·98) |
|  | Ureter | | 4 142 349/250 | 1·28 (0·79-2·08) | 0·98 (0·76-1·25) |  | 802 843/163 | NA^§^ | 0·99 (0·78-1·26) |  | NA^§^ | 1·04 (0·82-1·32) |  | 394 435/38 | | NA^§^ | NA^§^ |
|  | Bladder | | 4 142 349/12 105 | 1·03 (0·95-1·11) | 0·98 (0·95-1·01) |  | 802 843/7653 | 1·09 (1·00-1·20) | 0·99 (0·95-1·02) |  | 1·12 (1·02-1·23) | 1·01 (0·97-1·05) |  | 394 435/1902 | | 0·94 (0·77-1·14) | 0·98 (0·91-1·05) |
|  | Urothelial carcinoma | | 3 837 868/6122 | 1·08 (0·96-1·21) | 1·02 (0·98-1·06) |  | 679 192/3602 | 1·12 (0·98-1·29) | 1·01 (0·96-1·07) |  | 1·14 (1·00-1·31) | 1·03 (0·98-1·08) |  | 349 387/929 | | 0·91 (0·68-1·21) | 1·00 (0·90-1·11) |
| Hodgkin lymphoma | | | 4 142 349/2239 | 1·38 (1·13-1·67) | 1·18 (1·11-1·25) |  | 802 843/469 | 1·36 (0·92-2·02) | 1·08 (0·94-1·25) |  | 1·38 (0·93-2·04) | 1·09 (0·95-1·26) |  | 394 435/187 | | NA^§^ | 0·91 (0·71-1·17) |

Abbreviations: NW, normal weight

* Hazard ratios from Cox regression models with age as time scale, adjusted for baseline age (continuous), weight assessment from the Medical Birth Register (yes/no), mode of weight assessment, mode of height assessment, marital status, education level, and birth country, and stratified all models by sex (when men and women combined) and calendar year of birth. Cancer forms with hazard ratios of cancers for current vs. never smokers above 1·5 (shown in Table S4) were defined as smoking-related cancer in this study.

† For cancers that could only be identified by later international classification of disease (ICD) codes than ICD7, follow-up started at the year of the start of the respective ICD edition or at baseline, whichever came later.

‡ Hazard ratios from Cox regression models with additionally adjusted for smoking status in three categories (never/former/current).

§ The number of cancer cases was considered too low for analysis (<250 cases for categorical body mass index and <100 cases for per 5 kg/m^2^).

**Table S13. Hazard ratios (95% confidence interval) and E-values for point estimates and lower confidence limits of hazard ratios of potential obesity-related cancers associated with an increased risk for obesity vs. normal weight in the study**

| **Cancer category** | | **HR (95% CI)  Obesity vs. Normal weight^*^** | **E-value^#^ for**  **HR estimate** | **E-value^#^ for lower**  **95% confidence limit** |
| --- | --- | --- | --- | --- |
| Head and neck | |  |  |  |
|  | Tongue | 1·45 (1·09-1·91) | 2·26 | 1·40 |
|  | Nasal and paranasal sinuses | 1·55 (1·06-2·26) | 2·47 | 1·31 |
| Small intestine | | 1·55 (1·25-1·93) | 2·47 | 1·81 |
|  | Ileum | 2·34 (1·58-3·46) | 4·11 | 2·54 |
|  | Neuroendocrine | 2·04 (1·56-2·68) | 3·50 | 2·49 |
| Colon | |  |  |  |
|  | Adenocarcinoma | 1·38 (1·30-1·46) | 2·10 | 1·92 |
|  | Neuroendocrine | 1·53 (1·14-2·06) | 2·43 | 1·54 |
| Rectum/anus | |  |  |  |
|  | Adenocarcinoma | 1·20 (1·10-1·30) | 1·69 | 1·43 |
| Biliary tract | | 1·48 (1·26-1·74) | 2·32 | 1·83 |
|  | Extrahepatic bile ducts | 1·49 (1·07-2·08) | 2·34 | 1·34 |
| Vulva | | 2·43 (1·88-3·14) | 2·11 | 3·17 |
| Cervix | |  |  |  |
|  | Adenocarcinoma | 1·34 (1·08-1·65) | 2·01 | 1·37 |
| Penis | | 3·07 (2·28-4·14) | 5·59 | 3·99 |
| Renal cell | |  |  |  |
|  | Clear cell | 2·36 (2·04-2·73) | 4·15 | 3·50 |
|  | Papillary | 1·55 (1·01-2·38) | 2·47 | 1·11 |
|  | Chromophobe | 2·03 (1·29-3·19) | 3·48 | 1·90 |
| Endocrine organs | | 1·34 (1·24-1·45) | 2·01 | 1·79 |
|  | Thyroid-papillary | 1·22 (1·06-1·41) | 1·74 | 1·31 |
|  | Adrenal glands | 1·50 (1·03-2·17) | 2·37 | 1·21 |
|  | Parathyroid gland | 1·41 (1·23-1·62) | 2·17 | 1·76 |
|  | Pituitary gland | 1·57 (1·35-1·83) | 2·52 | 2·04 |
| Connective tissue | | 1·34 (1·11-1·62) | 2·01 | 1·46 |
| Lymphoid neoplasms | | 1·22 (1·14-1·31) | 1·74 | 1·54 |
|  | Hodgkin lymphoma | 1·38 (1·13-1·67) | 2·10 | 1·51 |
|  | Diffuse large B-cell | 1·51 (1·29-1·77) | 2·39 | 1·90 |
|  | Acute lymphocytic | 1·61 (1·13-2·29) | 2·60 | 1·51 |
| Leukaemia | | 1·42 (1·27-1·59) | 2·19 | 1·86 |
|  | Chronic myeloid | 1·47 (1·13-1·92) | 2·30 | 1·51 |

Abbreviations: HR, hazard ratio; CI, confidence interval

* Hazard ratios from Cox regression models with attained age as time scale, adjusted for baseline age (continuous), weight assessment from the Medical Birth Register (yes/no), mode of weight assessment, mode of height assessment, marital status, education level, and birth country, and stratified all models by sex and calendar year of birth. All these results can be found in Table 2.

# To assess the potential of residual confounding explaining away an association between obesity and cancer risk, we applied the E-value method, which estimates the minimum strength of association that a potential unmeasured confounder would need to have on both the exposure and outcome to fully explain away the observed association in terms of hazard ratio (HR). A large E-value implies that strong unmeasured confounding would be needed to explain away the observed association. For example, for tongue cancer, an unmeasured confounder would need to have an HR of at least 2·26 between both the categorical exposure and the outcome to explain the observed HR. Unadjusted known risk factors for tongue cancer include for example smoking and alcoholic drinking. The HR of tongue cancer for current vs. never smoking was 1·88 as shown in Supplementary Table 8. The risk ratio of oral cancer for moderate drinkers compared to non-drinkers was 1·40 (Bagnardi, V. et al, Alcohol consumption and site-specific cancer risk: a comprehensive dose-response meta-analysis. British journal of cancer, 2015, 112(3), 580–593.). This indicates that residual confounding by smoking and alcohol intake are unlikely to explain away the observed association between obesity and tongue cancer.

**Table S1****4. Hazard ratios (95% confidence interval) of established obesity-related cancers and potential obesity-related cancers according to body mass index level, in all individuals and with the exclusion of the Medical Birth Register and Swedish Military Conscription Register^*^**

| **Cancer category**^†^ | **All** | | |  | **Excluding the Medical Birth Register and military conscription** | | |
| --- | --- | --- | --- | --- | --- | --- | --- |
|  | **No. at risk/cases** | **Obesity vs. normal weight^*^** | **Per 5 kg/m^2^ higher BMI^*^** |  | **No. at risk/cases** | **Obesity vs. normal weight^*^** | **Per 5 kg/m^2^ higher BMI^*^** |
| **Men** |  |  |  |  |  |  |  |
| Established obesity-related cancer | 2 129 149/36 462 | 1·60 (1·53-1·67) | 1·24 (1·22-1·26) |  | 390 048/21 197 | 1·53 (1·46-1·61) | 1·24 (1·21-1·26) |
| Potential obesity-related cancer | 2 129 149/35 688 | 1·34 (1·27-1·41) | 1·17 (1·15-1·19) |  | 390 048/14 855 | 1·28 (1·20-1·37) | 1·14 (1·11-1·17) |
|  |  |  |  |  |  |  |  |
| **Women** |  |  |  |  |  |  |  |
| Established obesity-related cancer | 2 013 200/47 922 | 1·27 (1·22-1·31) | 1·12 (1·11-1·13) |  | 269 294/24 518 | 1·27 (1·22-1·32) | 1·13 (1·11-1·15) |
| Potential obesity-related cancer | 2 013 200/16 002 | 1·33 (1·25-1·40) | 1·13 (1·11-1·15) |  | 269 294/5596 | 1·26 (1·16-1·38) | 1·10 (1·07-1·14) |

* Hazard ratios from Cox regression models with attained age as time scale, adjusted for baseline age (continuous), weight assessment from the Medical Birth Register (yes/no, for analyses on all individuals only), mode of weight assessment, mode of height assessment, marital status, education level, and birth country, and stratified all models by sex (when men and women combined) and calendar year of birth.

† Established obesity-related cancers include cancers of the oesophagus (adenocarcinoma), stomach-cardia, colon, rectum, liver/intrahepatic bile ducts, gallbladder, pancreas, meningioma, thyroid, multiple myeloma and renal cell carcinoma for both men and women, and additionally includes cancers of the breast (postmenopausal), endometrium, and ovary for women. Potential obesity-related cancers include cancers of the oral cavity, nasal and paranasal sinuses, gastric-gastrointestinal stromal (excluding gastric-cardia), small intestine, biliary tract (excluding gallbladder), pancreatic islets, adrenal glands, parathyroid gland, pituitary gland, connective tissue, lymphoid neoplasms, and myeloid neoplasms for both men and women, and also includes cancer of the penis, melanoma, and head and neck (adenocarcinoma) for men and cancer of the melanoma (nodular), vulva, head and neck (squamous cell carcinoma), and cervix (adenocarcinoma) for women.

**4 142 349 individuals with 6 956 035 observations**

Men recorded with female cancers and women recorded with male cancers: 1 individual/1 observation

Height missing: 30 793 individuals/33 462 observations

**4 142 349 individuals with one selected observation**

In individuals with ≥ 2 health examinations: selection of the first observation with information on smoking status (if available)

⮩

⮩

⮩

⮩

⮩

⮩

**4 295 859 individuals with 7 733 901 observations**

⮩

Recalled weight: 0 individuals/500 219 observations

Extreme values for weight (<35 or >250 kg), height (<100 or >250 cm),

or BMI (<15 or >60 kg/m^2^): 1446 individuals/3612 observations

Date of health examination after Dec. 31, 2019:

173 individuals/1 633 observations

Non-matching dates: death/emigration ≤ health examination, death < emigration/cancer diagnosis: 99 842 individuals/169 369 observations

Date of cancer diagnosis ≤ date of health examination:
21 252 individuals/69 570 observations

**Figure S1. Flowchart of exclusions and selections of individuals and observations in the study.**

⮩ denotes exclusions


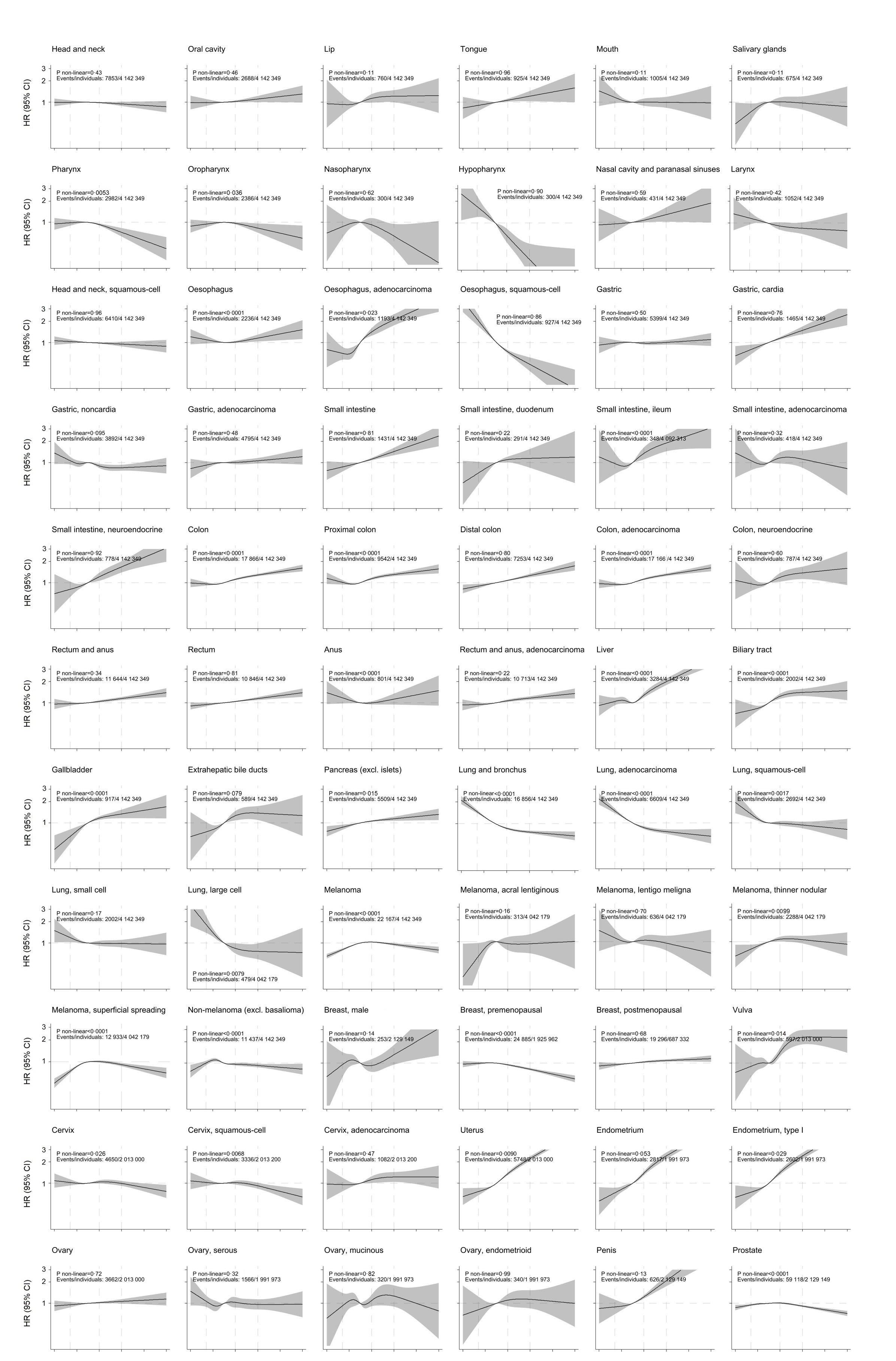


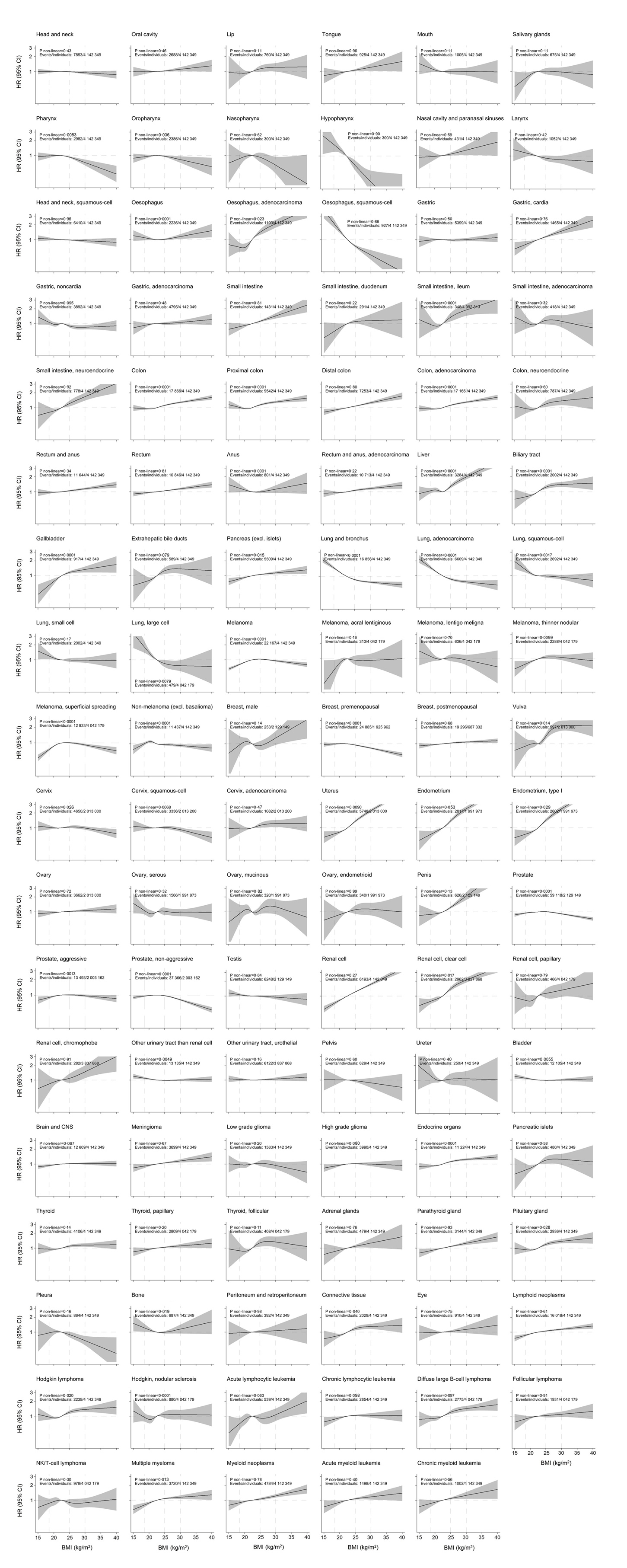


**Figure S2. Hazard ratios of site-specific cancers according to BMI, allowing for non-linear effects, with 95% confidence intervals.** The reference BMI for these plots (with HR fixed as 1**·**0) was 22**·**5 kg/m². For each cancer form, models with three, four, five, six, and seven knots were fitted, and the Akaike Information Criterions (AIC) were calculated; the number of knots resulting in the minimum AIC then are chosen. Person-years at risk were counted from baseline until the diagnosis of a cancer, or until censoring due to another cancer, death, emigration, or until the end of follow-up, whichever came first. For “premenopausal” breast cancer, person-years were counted from baseline until the diagnosis of breast cancer, or until censoring due to another cancer, death, emigration, or until the 55^th^ birthday, whichever came first. For “postmenopausal” breast cancer, person-years were counted from age 55 years onwards or from the date of health examination if this occurred after the 55^th^ birthday. For cancers that could only be defined by later international classification of disease (ICD) codes than ICD-7, follow-up started at the year of the start of the later ICD edition or at baseline, whichever came later. Restricted cubic splines for BMI with knots placed at Harrell's recommended percentiles of BMI were fitted adjusting for baseline age (continuous), weight assessment from the Medical Birth Register (yes/no), mode of weight assessment, mode of height assessment, marital status, education level, and birth country, and stratified by sex and calendar year of birth. Non-linearity was assessed, testing the null hypothesis of equal spline coefficients using the post-estimation Wald test. HR, hazard ratio; CI, confidence interval; BMI, body mass index.


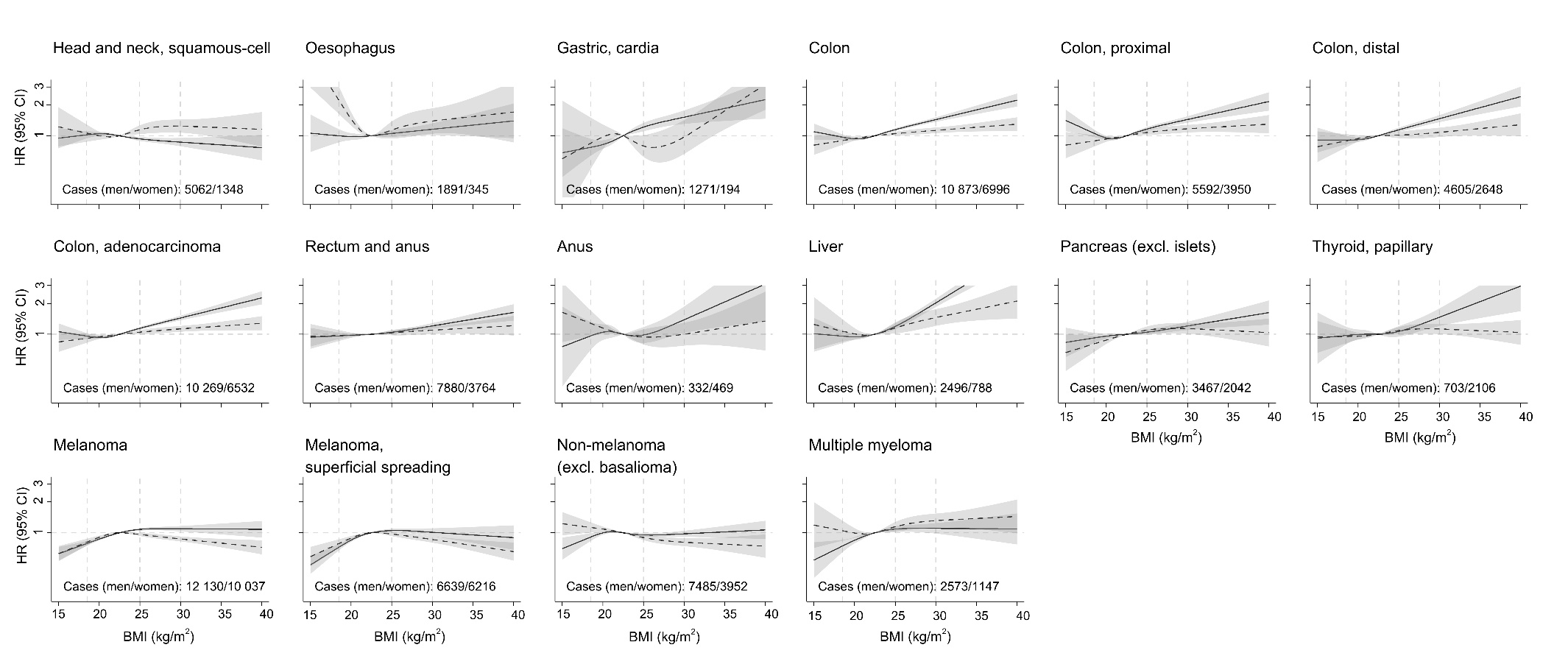


**Figure S3. Hazard ratios of cancers separately for men (solid line) and women (dashed line), for cancers with a differential association by sex according to BMI, allowing for non-linear effects, with 95% confidence intervals.** The reference BMI for these plots (with HR fixed as 1**·**0) was 22**·**5 kg/m². For each cancer form, models with three, four, five, six, and seven knots were fitted, and the Akaike Information Criterions (AIC) were calculated; the number of knots resulting in the minimum AIC then are chosen. Person-years at risk were counted from baseline until the diagnosis of a cancer, or until censoring due to another cancer, death, emigration, or until the end of follow-up, whichever came first. For cancers that could only be defined by later international classification of disease (ICD) codes than ICD-7, follow-up started at the year of the start of the later ICD edition or at baseline, whichever came later. Restricted cubic splines for BMI with knots placed at Harrell's recommended percentiles of BMI were fitted adjusting for baseline age (continuous), weight assessment from the Medical Birth Register (yes/no), mode of weight assessment, mode of height assessment, marital status, education level, and birth country, and stratified by calendar year of birth. HR, hazard ratio; CI, confidence interval; BMI, body mass index.

**
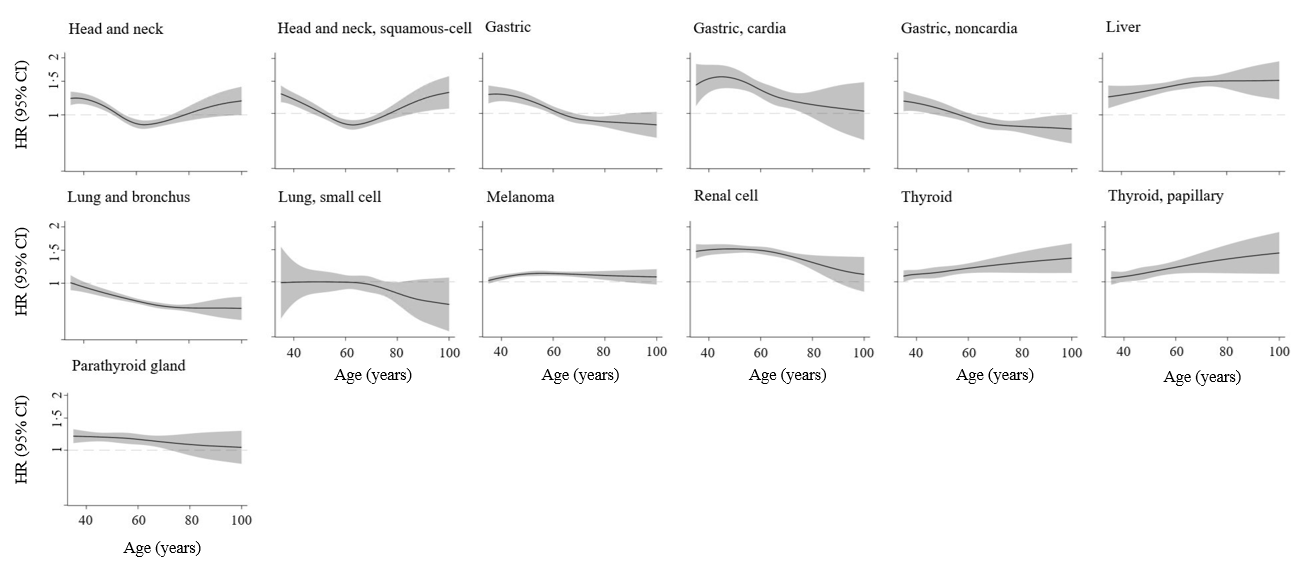
 Figure S4. Age-dependent hazard ratios with 95% confidence intervals for cancers for which the proportional hazards assumption was indicated to be violated per 5 kg/m² higher body mass index.** For cancers that could only be defined by later international classification of disease (ICD) codes than ICD-7, follow-up started at the year of the start of the later ICD edition or at baseline, whichever came later. Calculations are based on flexible parametric survival models with age as time scale, adjusted for baseline age (continuous), weight assessment from the Medical Birth Register (yes/no), mode of weight assessment, mode of height assessment, marital status, education level, birth country, and calendar year of birth, with three degrees of freedom in the baseline hazard and three degrees of freedom in the time dependent effect of body mass index. HR, hazard ratio; CI, confidence interval.
